# Comprehensive Metabolite Profile and Cytotoxic Constituents of *Cryptolepis decidua*

**DOI:** 10.1021/acs.jnatprod.6c00033

**Published:** 2026-04-24

**Authors:** Tobias Blank, Charlie Puth, Annikka Kurz, Moritz Benka, Malik Rakhmanov, Iwanette Du Preez-Bruwer, Davis Mumbengegwi, Dietmar A. Plattner, Bernd Kammerer, Roman Huber, Robin Teufel, Olivier Potterat, Volker M. Lüth

**Affiliations:** † Pharmaceutical Biology, Department of Pharmaceutical Sciences, 27209 University of Basel, Basel 4056, Switzerland; ‡ Centre for Complementary Medicine, Department of Internal Medicine II, Faculty of Medicine, 88751 University of Freiburg, Freiburg 79106, Germany; § Core Competence Metabolomics, Hilde Mangold Haus, 9174 University of Freiburg, Freiburg 79104, Germany; ∥ Institute of Organic Chemistry, 9174 University of Freiburg, Freiburg 79104, Germany; ⊥ Multidisciplinary Research Centre, 99404 University of Namibia, 340 Mandume Ndemufayo Avenue, Pioneers Park, Windhoek 13301, Namibia

## Abstract

Root extracts of *Cryptolepis decidua* are used in the traditional medicine of South-West Africa to treat various conditions. Here, we report the potent cytotoxic activity of a methanol root extract against a human adenocarcinomic cell line, which prompted us to establish a comprehensive metabolite profile. Overall, six previously undescribed compounds were isolated from this extract by a combination of open-column chromatography (CC), centrifugal partition chromatography (CPC), and preparative HPLC. They include two sarmentogenin-type cardenolides (**2** and **3**), an androstane lactone (**5**), two dinormonoterpenoid diglucosides, dinosides A and B (**9** and **10**), and a quinic acid derivative (**12**). In addition, six previously reported constituents were identified (**1**, **4**, **6**–**8**, **11**). The structures of the isolates were elucidated using HPLC-MS^2/3^, HRESIMS, and NMR spectroscopy. Feature-based molecular networking was used to map a comprehensive metabolite profile. The cytotoxic activity of the methanol extract was evaluated by WST-1 cell-viability assays, apoptosis/necrosis tests, and cell-cycle analyses. Cardenolides **1**–**3** were identified as the primary cytotoxic agents, exhibiting IC_50_ values of 0.25 μM (**1**), 2.20 μM (**2**), and 0.28 μM (**3**) in A549 human lung epithelial cancer cells, and 0.41 μM (**1**), 2.31 μM (**2**), and 0.39 μM (**3**) against peripheral blood mononuclear cells (PBMCs).

## Graphical abstract

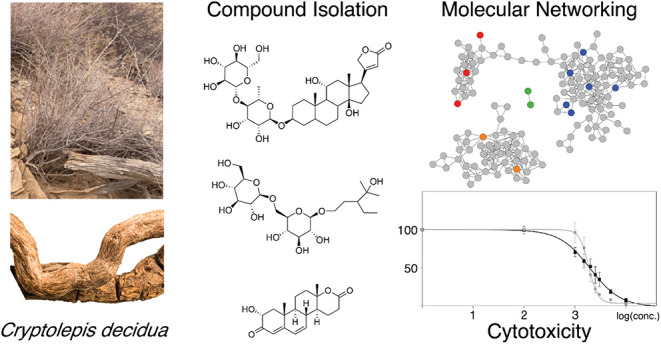

C*ryptolepis decidua* (Planch. ex Benth.) N.E.Br. (Apocynaceae), a shrub native to Southwest Africa, is one of 41 members of the genus *Cryptolepis*.^1^ While the Apocynaceae family is well-known for its cytotoxic constituents, particularly cardenolides and indole alkaloids, which are common across many of its genera,^2,3^ information on the constituents of *Cryptolepis* spp. remains scarce.

Indoloquinoline alkaloids, notably cryptolepines, have been identified in *C. sanguinolenta* (Lindl.) Schltr., a West African species widely used in traditional medicine and marketed as a dietary supplement.^4^ In addition, cardenolides, as well as pregnane and androstane steroids, have been reported in *C. dubia* (Burm.f.) M.R. Almeida (syn. *C. buchananii* Roem. and Schult.) growing in South and Southeast Asia.^5–7^ Phenolic compounds and nonpolar terpenoids and steroids were detected in a series of African species as part of a general phytochemical screening.^8^ However, no data exist on the phytochemical constituents of *C. decidua*. In Namibia and Botswana, root decoctions of *C. decidua* are traditionally used to treat gastrointestinal disorders, stomach aches, and skin rashes.^9^ In a previous study, we showed that root extracts inhibited T-lymphocyte proliferation, suggesting an anti-inflammatory mode of action,^10^ but the constituents of the extracts remained uncharacterized.

In a continuation of our investigations, we now report that the methanol extract of the roots also exhibits strong cytotoxic activity against the human adenocarcinomic alveolar basal epithelial cell line A549. This prompted us to undertake a comprehensive investigation of *C. decidua* in order to unravel its specialized metabolite profile and identify its cytotoxic constituents. For this purpose, an approach combining HPLC-MS^2/3^ analysis, molecular networking, HPLC-based activity profiling, and preparative chromatography was applied, which resulted in the isolation and characterization of 12 structurally diverse compounds, including six previously undescribed metabolites and the identification of the main cytotoxic constituents.

## Results and Discussion

For initial HPLC-MS screening, the crude MeOH extract of the dry roots was chromatographed with a 5–100% MeCN gradient containing 0.1% formic acid (FA), and detection was performed by high-resolution mass spectrometry (Thermo LTQ Orbitrap XL). The chromatogram (LC-MS^1^) in both positive or negative mode showed three comparatively intense peaks (*t*_R_ = 11.28, 12.59, and 13.70 min, Supporting Information Figure S1), whose *m*/*z* and fragmentation patterns (*vide infra*) suggested the presence of cardenolides.

For structural characterization, the MeOH extract was fractionated by Sephadex LH-20 CC, followed by a combination of CPC, preparative and semipreparative HPLC, which yielded 12 compounds. These included the three suspected cardenolides (**1–3**), two androstane steroids (**4** and **5**), three pregnane steroids (**6–8**), two dinormonoterpenoids (**9** and **10**), and two cinnamoyl quinic acid esters (**11** and **12**). By comprehensive spectroscopic analysis and comparison with literature data, compounds **1** and **6–8** were identified as 5β-sarmentogenin-3-*O*-[β-d-glucopyranosyl-(1→4)-α-l-oleandroside] (glucosyl divaricoside) (**1**),^11,12^ 21-hydroxypregna-4,6-diene-3,12,20-trione (**6**),^13^ (+)-2,21-dihydroxypregna-4,6-diene-3,20-dione (**7**),^5^ and 21-hydroxypregna-4,6-diene-3,20-dione (**8**).^14^ Their ^1^H and ^13^C NMR data are reported in the Supporting Information (Tables S3–S6). The structure of compound **4** was previously disclosed as 2-hydroxy-3,17-dioxo-androsta-4,6-diene in a US patent,^15^ but its relative configuration was not described, and its spectroscopic data are reported here for the first time. Compound **11**, identified as 1-*O*-feruloyl-3-*O*-caffeoylquinic acid (atom numbering according to IUPAC 1976),^16^ is listed in SciFinder (Chemical Abstract Service, CAS) in connection with references reporting LC-MS and LC-MS^2^ analyses^17,18^ but, to our knowledge, it had not yet been isolated. Except for compound **7**, which has been recently reported from *C. dubia*,^5^ none of the reported compounds have been previously found in the genus *Cryptolepis* and **2**, **3**, **5**, **9, 10**, and **12** furthermore possess previously undescribed structures.
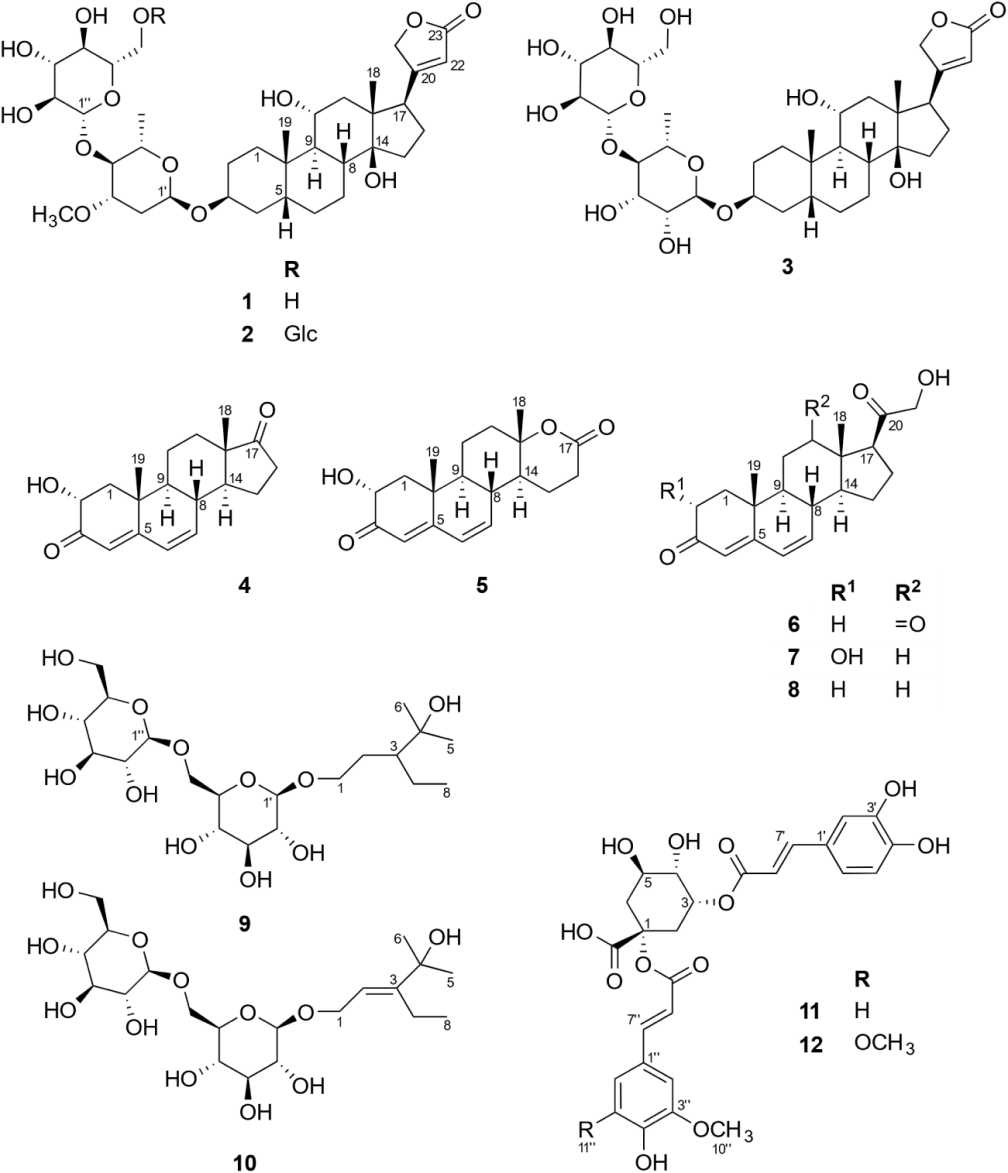


Compound **2** had the molecular formula C_42_H_66_O_18_, which was determined based on the formate adduct ion at *m*/*z* 903.4273 [M + FA – H]^−^ in the HRESIMS spectrum (calcd for C_43_H_67_O_20_, 903.4231) in conjunction with ^13^C and DEPT NMR data (Table 1). The ^1^H NMR spectrum showed characteristic signals for a butenolide ring [δ_H_ 4.93 and 5.01 (m, H_2_-21), and 5.92 (s, H-22)], and two angular methyl groups [δ_H_ 0.90 (3H, s, H-18) and 1.07 (3H, s, H-19)]. The ^13^C NMR spectrum revealed the presence of 42 carbon signals, consisting of four methyl groups, 12 methylenes, 21 methines, and five nonprotonated carbons, which were consistent with a cardenolide glycoside containing three sugar moieties. Comparison with NMR data reported for glucosyl divaricoside (**1**) revealed nearly identical aglycone signals,^11,12^ suggesting a 5β-sarmentogenin-type cardenolide. One oxygen-substituted methine was assigned to C-3 (δ_C_ 74.0), based on the COSY correlations between both H_2_-2 [δ_H_ 1.53 and 1.82 (m)] and H_2_-4 [δ_H_ 1.43 (m) and 1.84 (m)] and Hα-3 (δ_H_ 3.89, m), as well as HMBC correlations between H-1′ (δ_H_ 4.97, d, *J* = 2.8 Hz) and C-3 (Figure 1). A hydroxyl group was located at C-11 on the basis of the HMBC correlations between C-11 and the protons H-8 (δ_H_ 1.66), H-9 (δ_H_ 1.79), and H_2_-12 (δ_H_ 1.56 and 1.68). The *cis* diaxial arrangement of H-5 (δ_H_ 1.63) and Me-19 (δ_H_ 1.07, s) was confirmed by a ROESY correlation between these protons (Figure 2). A ROESY correlation between H-17 (δ_H_ 2.91, m) and Me-18 (δ_H_ 0.90, s) indicated the α-orientation of the butenolide ring. The β-orientation of the hydroxyl group at C-3 was determined based on the chemical shift and multiplicity of H-3 (δ_H_ 3.89, br s)^19^ and was also in line with the absence of a cross-peak between H-3 and Me-19 in the ROESY spectrum. The α-orientation of HO-11 was revealed through ROESY cross-peaks between both Me-18 and Me-19 and H-11. ^13^C NMR signals for the l-oleandrose and d-glucose moieties were comparable to those observed in **1** and the 1,4-glycosidic linkage was supported by the HMBC correlations H-1″/C-4′ [δ_H_ 4.64 (d, *J* = 7.6 Hz)/δ_C_ 83.0] and H-4′/C-1″ [δ_H_ 3.40 (s)/δ_C_ 105.0]. The additional 162 amu compared to **1** together with the ^13^C NMR data indicated the presence of a second β-glucopyranosyl moiety which was located at C-6″ based on the HMBC correlations H-1‴/C-6″ [δ_H_ 4.40 (d, *J* = 7.9 Hz)/δ_C_ 70.6] and H_2_-6″/C-1‴ [δ_H_ 3.78 and 4.14 (dd, *J* = 11.6 and 1.8 Hz)/δ_C_ 105.2]. This was also supported by the downfield shift of C-6″ compared to **1**. The absolute configuration of the d-glucopyranosyl residues was confirmed by GC-MS analysis after derivatization with (+)-2-butanol and acetyl chloride. The structure of **2** was thus established as 5β-sarmentogenin 3-*O*-[β-d-glucopyranosyl-(1→6)-β-d-glucopyranosyl-(1→4)-α-l-oleandroside].

**Figure 1:**
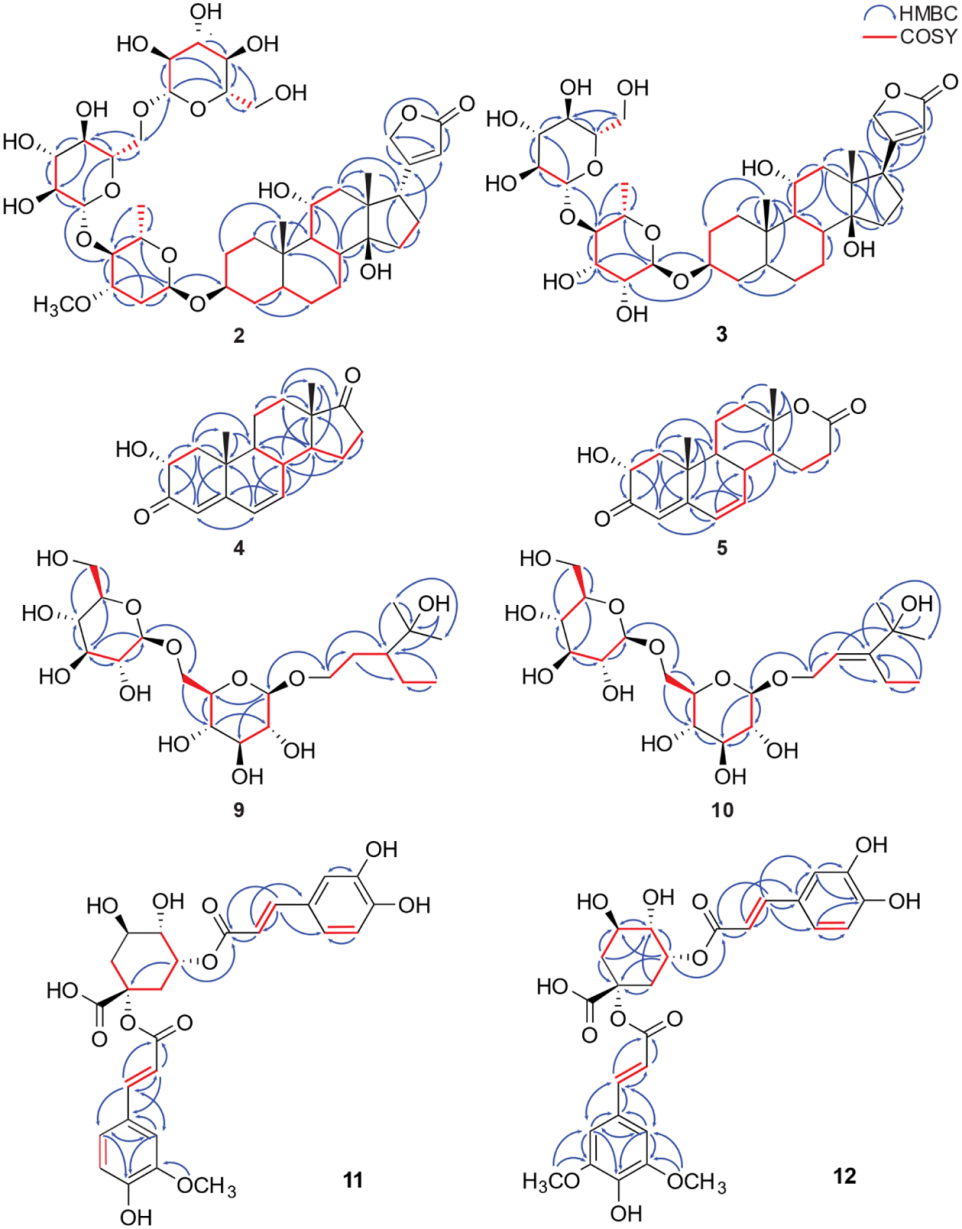
Key ^1^H–^13^C HMBC and ^1^H–^1^H COSY correlations of compounds **2–5** and **9–12**.

**Table 1:** ^13^C (125 MHz) and ^1^H (500 MHz) NMR Data of Compounds **2** and **3** (CD_3_OD)^a^

	2				3			
Position	δ_C_	Type	δ_H_	(*J* Hz)	δ_C_	Type	δ_H_	(*J* Hz)
1	34.3	CH_2_	1.40		34.3	CH_2_	2.36	br d (13.4)
			2.35				1.39	m
2	28.5	CH_2_	1.53		28.5	CH_2_	1.57	m
			1.82	m			1.83	m
3	74.0	CH	3.89	br s	74.5	CH	3.94	m
4	31.8	CH_2_	1.43	m	31.9	CH_2_	1.45	m
			1.84	m			1.86	m
5	39.9	CH	1.63		40.0	CH	1.62	br d (13.7)
6	28.4	CH_2_	1.91	br dd (9.2, 5.2)	28.1	CH_2_	1.90	br d (9.5)
			1.27	m			2.18	m
7	22.8	CH_2_	1.28	m	22.8	CH_2_	1.81	m
			1.79	m			1.28	s
8	42.0	CH	1.66		42.2	CH	1.65	br d (3.1)
9	43.1	CH	1.79		43.3	CH	1.79	m
10	37.7	C			37.8	C		
11	69.0	CH	3.73	m	69.0	CH	3.75	m
12	50.7	CH_2_	1.56		50.7	CH_2_	1.68	m
			1.68				1.56	m
13	51.2	C			51.2	C		
14	85.7	C			85.7	C		
15	33.8	CH_2_	2.22		33.9	CH_2_	1.75	m
			1.76				2.21	m
16	28.1	CH_2_	1.90		28.4	CH_2_	1.25	br d (2.8)
			2.19				1.89	m
17	52.0	CH	2.91	m	52.1	CH	2.90	m
18	17.7	CH_3_	0.90	s	17.7	CH_3_	0.90	s
19	24.7	CH_3_	1.07	s	24.7	CH_3_	1.07	s
20	177.8	C			177.7	C		
21	75.5	CH_2_	4.93	dd (18.6, 1.8)	75.5	CH_2_	4.91	dd (18.6, 1.8)
			5.01	dd (18.6, 1.5)			5.01	dd (18.6, 1.2)
22	118.1	CH	5.92	s	118.1	CH	5.90	s
23	177.3	C			177.2	C		
1′	97.1	CH	4.97	d (2.8)	100.1	CH	4.77	d (1.2)
2′	36.4	CH_2_	2.22		73.1	CH	3.79	m
			1.51					
3′	80.2	CH	3.74		72.8	CH	3.93	m
4′	83.0	CH	3.40	s	84.0	CH	3.61	m
5′	68.4	CH	3.75		68.8	CH	3.75	m
6′	18.7	CH_3_	1.26	d (6.4)	18.2	CH_3_	1.31	d (6.4)
7′	57.1	CH_3_	3.41	m				
1″	105.0	CH	4.64	d (7.6)	106.0	CH	4.58	m
2″	75.9	CH	3.17		76.3	CH	3.23	m
3″	78.1	CH	3.36		78.5	CH	3.38	m
4″	72.0	CH	3.31		71.9	CH	3.33	m
5″	77.2	CH	3.43		78.2	CH	3.28	m
6″	70.6	CH_2_	3.78		63.1	CH_2_	3.85	m
			4.14	dd (11.6, 1.8)			3.70	m
1‴	105.2	CH	4.40	d (7.9)				
2‴	75.3	CH	3.21					
3‴	78.0	CH	3.36					
4‴	71.7	CH	3.28					
5‴	78.1	CH	3.26					
6‴	62.9	CH_2_	3.66	dd (11.9, 5.5)				
			3.86	dd (11.8, 2.0)				

a Overlapped signals are reported without multiplicity.

**Figure 2:**
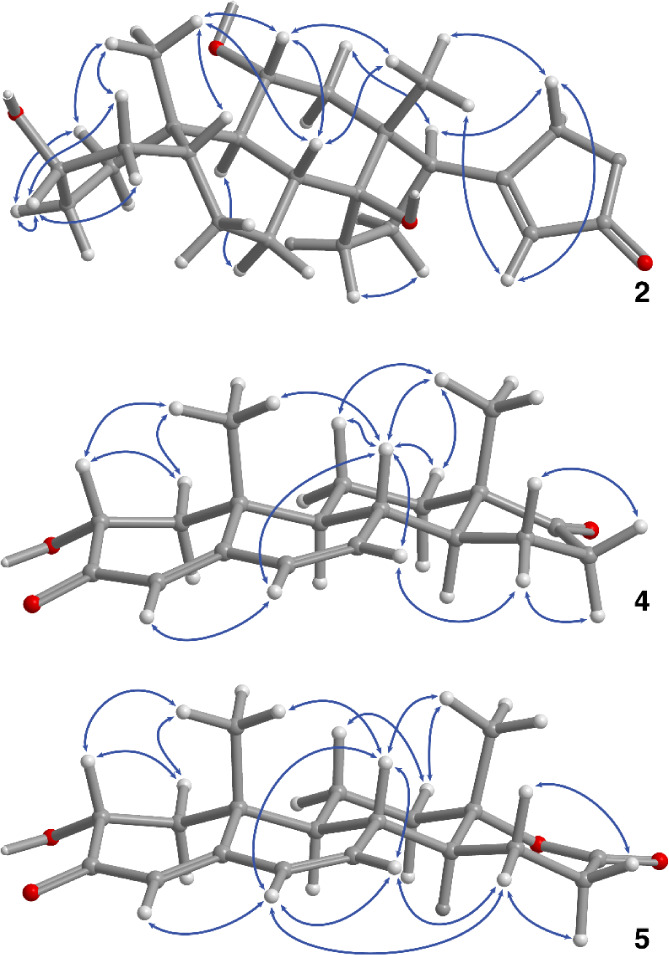
^1^H–^1^H ROESY correlations of the aglycone of compound **2**, as well as compounds **4** and **5**.

Compound **3** was assigned the molecular formula C_35_H_54_O_14_ based on the formate adduct ion at *m*/*z* 743.3531 [M + FA – H]^−^ (calcd for C_36_H_55_O_16_, 743.3496) in combination with ^13^C and DEPT NMR data (Table 1). The ^1^H and ^13^C NMR data of the aglycone moiety were highly similar to those of compounds **1** and **2**, indicating that **3** was also a 5β-sarmentogenin glycoside. Acid hydrolysis afforded d-glucose and l-rhamnose. The NMR data of the glycosyl moiety were comparable to those reported for 5α-sarmentogenin 3-*O*-[β-d-glucopyranosyl-(1→4)-α-l-rhamnopyranoside].^20^ HMBC correlations from H-4′ (δ_H_ 3.61, m) to C-1″ (δ_C_ 106.0) and H-1″ (δ_H_ 4.58, m) to C-4′ (δ_C_ 84.0) confirmed the interglycosidic linkage. The β-orientation of H-5 (δ_H_ 1.62, br d, *J* = 13.7 Hz) was supported by the ROESY correlation between H-5 and Me-19 (δ_H_ 1.07, 3H, s) (comparable to **2**, Figure 2). Thus, compound **3** was identified as 5β-sarmentogenin 3-*O*-[β-d-glucopyranosyl-(1→4)-α-l-rhamnopyranoside], the 5-epimer of the previously reported glycoside mentioned above.^20^

The nature of cardenolides **1**–**3** was further interrogated and confirmed by LC-MS and MS^2^ experiments in positive mode, where all three peaks exhibited lower [M + H]^+^ and higher [M + NH_4_]^+^ signal intensities (Supporting Information Table S1). In addition, in-source fragmentation occurred (data not shown). The signal corresponding to protonated sarmentogenin was observed in each case in addition to further signals corresponding to single, double, or triple water elimination from sarmentogenin. In this respect, the LC-MS experiment in positive mode can be considered a pseudo-MS^2^ experiment indicating the aglycone sarmentogenin. Signals corresponding to the elimination of MeOH were observed for oleandrosides **1** and **2**.

In the LC-MS^2^ experiments in positive mode, only protonated sarmentogenin (as in-source fragment) could be fragmented by collision-induced dissociation (CID) (data not shown). The fragmentation of protonated sarmentogenin yielded only signals that could be assigned to water eliminations, which is why LC-MS^2^ experiments in positive mode did not provide any added value. The LC-MS experiment in negative mode showed the unfragmented di- or trisaccharide of sarmentogenin (in decreasing order of intensity) as [M + FA – H]^−^, [M + NO_3_]^−^, [M + Cl]^−^ or as [M – H]^−^ for peaks #1–3 (Supporting Information Figure S1 and Table S1). In the LC-MS^2/3^ experiments in negative mode (also using CID), for **1** only [M + FA – H]^−^, and for **2** and **3** only [M – H]^−^ yielded meaningful fragmentation data (Supporting Information Table S2). In order to obtain [M – H]^−^ in satisfactory intensities, ion source fragmentation (ISF) was applied. In addition to glucose and rhamnose cleavage, the first fragmentation of the parent ion (MS^2^) yielded characteristic fragments due to C–C bond breaks for glucorhamnoside **3** (Figure 3). This includes the elimination of CO_2_, cleaved from the butenolide substituent, which can theoretically lead to several products. As remaining substituent, the cyclopropenyl ring is shown but the open-chain diradical or carbene is also conceivable. Another significant fragmentation is the elimination of C_2_H_2_. The precondition of this elimination is a water elimination of the OH group at C-11 with a proton at C-12. The resulting olefin function shows only one C–C single bond at C-11 and one at C-12, which enables the subsequent elimination of C_2_H_2_. Again, this elimination can theoretically lead to several products like the shown conjugated diene, the open-chain diradical or a cyclobut­(en)­yl substructure. Since the signal for the C_2_H_2_ elimination was only seen in combination with a second water elimination it was assumed that this water elimination was derived from the OH function at C-14 and the proton at C-8.

**Figure 3:**
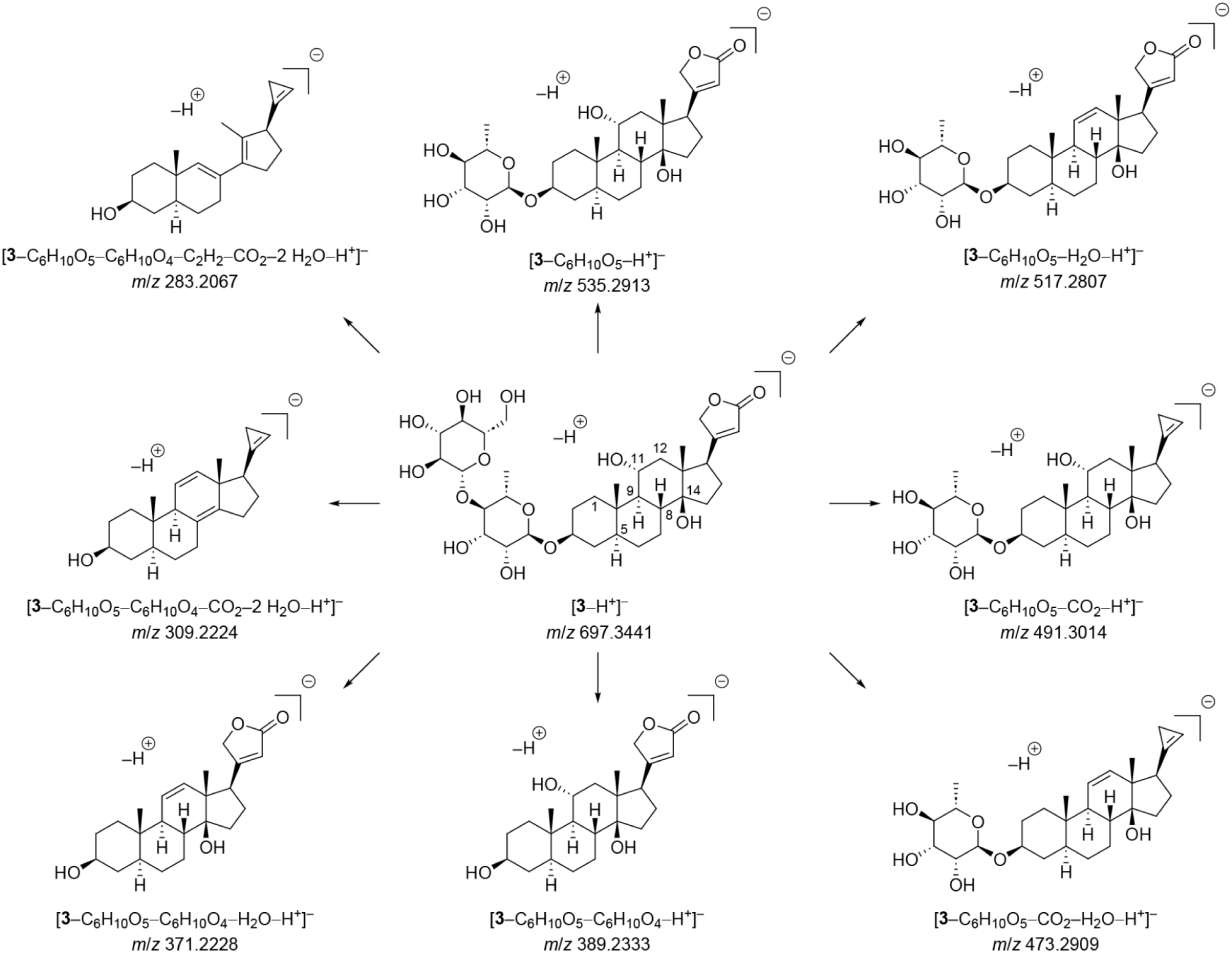
Putative MS^2^ fragment ions of sarmentogenin-3-*O*-glucorhamnoside (**3**) with their theoretical *m*/*z* value.

The resulting olefin function enables the above-described elimination of C_2_H_2_ as a part of a cycloreversion, which forms the shown conjugated diene. It is also plausible that the water elimination at C-14 and C-8 occurs prior to that at C-11 and C-12. The further fragmentation of the monosaccharide obtained from the MS^2^ experiment (MS^3^) yielded the same fragments. In the cases of **1** and **2**, fewer fragments were obtained from the first fragmentation of the respective parent ion (MS^2^), one of which was the monosaccharide. Although its further fragmentation (MS^3^) yielded fewer fragments than for the monosaccharide starting from **3**, in both cases the characteristic C_2_H_2_ and CO_2_ eliminations were observed (in addition to oleandrose cleavage and water elimination). Taking all NMR and MS data into account, the cardenolides were assigned as sarmentogenin-3-*O*-glucooleandroside **1**, sarmentogenin-3-*O*-glucoglucooleandroside **2** and sarmentogenin-3-*O*-glucorhamnoside **3**.

The molecular formula C_19_H_24_O_3_ was assigned to **4** based on the protonated molecular ion at *m*/*z* 301.1793 [M + H]^+^ in the HRESIMS spectrum (calcd for C_19_H_25_O_3_, 301.1798) in combination with ^13^C and DEPT NMR data. A UV absorption maximum at 282 nm was consistent with the presence of a dienone chromophore as in compounds **6**–**8**. The ^1^H NMR data revealed two methyl groups [δ_H_ 0.99 (3H, s, H-18) and 1.28 (3H, s, H-19)], an oxygenated methine (δ_H_ 4.44, dd, *J* = 13.1 and 5.5 Hz, H-2β), and three sp^2^ methines [δ_H_ 5.71 (s, H-4), 6.29 (dd, *J* = 10.7 and 2.8 Hz, H-6) and 6.31 (dd, *J* = 9.8 and 1.5 Hz, H-7)]. The ^13^C NMR spectrum showed signals for two methyl groups, five methylenes, seven methines, and five nonprotonated carbons, including two carbonyls (Table 2). These data were in line with an androstane skeleton, which was further supported by comparison with literature data. The diene was located at C-4(5) (δ_C_ 122.8 and 166.3) and C-6(7) (δ_C_ 129.0 and 141.1) by the COSY correlation between H-8 (δ_H_ 2.48) and H-7 (δ_H_ 6.31) and key HMBC correlations including H-6/C-8, H-5/C-5, H-6/C-4 and H-4/C-6 (Figure 1). The presence of a conjugated carbonyl group at C-3 was confirmed by the HMBC cross-peak between H-1 and C-3 (δ_C_ 201.6). The position of the carbonyl group at C-17 was supported by the HMBC correlation between H-12 (δ_H_ 1.32) and C-17 (δ_C_ 214.1). The methyl groups C-18 (δ_C_ 14.2) and C-19 (δ_C_ 17.6) were assigned to C-13 (δ_C_ 49.8) and C-10 (δ_C_ 39.5), respectively, based on HMBC correlations of H-18 with C-12 (δ_C_ 32.6) and C-13, and of H-19 with C-1 (δ_C_ 44.4), C-9 (δ_C_ 52.6), and C-10. The presence of a hydroxyl group at C-2 (δ_C_ 70.9) was supported by HMBC correlations from both H_2_-1­[δ_H_ 1.65 (t, *J* = 12.8 and 12.8 Hz) and 2.31 (dd, *J* = 12.5 and 5.5 Hz)] and H-4 to C-2. Finally, the α-orientation of the hydroxyl group at C-2 was established by a ROESY cross-peak between Hβ-2 and Me-19 (Figure 2). In conclusion, compound **4** was identified as 2α-hydroxy-3,17-dioxo-androsta-4,6-diene. While the planar structure of **4** has been mentioned in a patent,^15^ its configuration and spectroscopic data are reported here for the first time.

**Table 2:** ^13^C (125 MHz) and ^1^H (500 MHz) NMR Data of Compounds **4** and **5** (CD_3_OD)^a^

	4				5			
Position	δ_C_	Type	δ_H_	(*J* Hz)	δ_C_	Type	δ_H_	(*J* Hz)
1	44.4	CH_2_	1.65	pseudo t (12.8)	44.1	CH_2_	1.68	
			2.31	dd (12.5, 5.5)			2.32	dd (12.4, 5.7)
2	70.9	CH	4.44	dd (13.1, 5.5)	70.9	CH	4.44	dd (13.4, 5.5)
3	201.6	C			201.5	C		
4	122.8	CH	5.71	s	122.8	CH	5.73	s
5	166.3	C			165.5	C		
6	129.0	CH	6.29	dd (10.1, 2.8)	129.3	CH	6.29	dd (10.1, 2.8)
7	141.1	CH	6.31	dd (10.1, 1.5)	139.6	CH	6.35	dd (10.1, 1.5)
8	38.1	CH	2.49		40.6	CH	2.15	br t (10.7, 10.7)
9	52.6	CH	1.34		51.1	CH	1.48	m
10	39.5	C			39.4	C		
11	21.2	CH_2_	1.55		22.6	CH_2_	1.44	
			1.71				1.83	
12	32.6	CH_2_	1.32	br d (4.6)	40.3	CH_2_	1.71	
			1.85	m			2.02	dt (12.7, 3.1, 3.1)
13	49.8	C			84.9	C		
14	50.0	CH	1.55		44.9	CH	1.67	
15	22.4	CH_2_	2.18		20.4	CH_2_	1.78	
			1.79				2.25	
16	36.6	CH_2_	2.52		29.2	CH_2_	2.64	m
			2.14				2.79	m
17	214.1	C			174.3	C		
18	14.2	CH_3_	0.99	s	20.3	CH_3_	1.43	s
19	17.6	CH_3_	1.28	s	17.5	CH_3_	1.24	s

a Overlapped signals are reported without multiplicity.

Compound **5** had the molecular formula C_19_H_24_O_4_, which was determined based on the protonated molecular ion at *m*/*z* 317.1744 [M + H]^+^ (calcd for C_19_H_25_O_4_, 317.1747) alongside ^13^C and DEPT NMR data (Table 2). The ^1^H NMR data displayed signals for two methyl groups [δ_H_ 1.43 (3H, s, H-18) and 1.24 (3H, s, H-19)], an oxygenated methine (δ_H_ 4.44, dd, *J* = 13.4 and 5.5 Hz, H-2β), and three sp^2^ methines [δ_H_ 5.73 (s, H-4), 6.29 (dd, *J* = 10.1 and 2.8 Hz, H-6) and 6.35 (dd, *J* = 10.1 and 1.5 Hz, H-7)]. The ^13^C NMR spectrum showed signals for two methyl carbons, five methylenes, seven methines, and five nonprotonated carbons, including two carbonyls. 1D and 2D NMR data were comparable to those of compound **4**, suggesting a similar steroid core with identical rings A-C. However, a nonprotonated carbon was downfield shifted to δ_C_ 84.9 (C-13) while one of the two carbonyl groups was observed at a higher field (δ_C_ 174.3, C-17), suggesting the presence of a δ-lactone ring, which was confirmed by HMBC correlations (Figure 1). Altogether, these data support the structure of **5** to be 2α-hydroxy-d-homo-17a-oxaandrosta-4,6-diene-3,17-dione, representing a scaffold that is reported for the first time from a plant source.

Compound **9** exhibited the molecular formula C_20_H_38_O_12_, which was determined based on the formate adduct ion *m*/*z* 515.2362 [M + FA – H]^−^ (calcd for C_21_H_39_O_14_, 515.2345) and further supported by ^13^C and DEPT NMR data (Table 3). The NMR signals were consistent with the presence of two β-glucopyranosyl moieties. In addition, the ^1^H NMR spectrum of compound **9** showed signals for three methyl groups [δ_H_ 1.13 (3H, s, H_3_-5), 1.18 (3H, s, H_3_-6), and 0.97 (3H, t, *J* = 7.6 Hz, H_3_-8)], and one oxygenated methylene [δ_H_ 3.59 (m) and 3.96 (td, *J* = 9.2, 5.5 Hz), H_2_-1]. The ^13^C NMR spectrum revealed the presence of three methyls, three methylenes, one methine, and one nonprotonated carbon. These spectral features, together with the observed HMBC and COSY correlations (Figure 1), suggested a 3-(2-hydroxyisopropyl)­pentan-1-ol glycosidic structure and closely resembled those of a dinormonoterpenoid previously described in *Cerbera manghas* L.,^21^ with the exception that **9** contains an additional glucose moiety, in β-pyranosyl configuration, which was established by the coupling constant of the anomeric proton (δ_H_ 4.37, d, *J* = 7.6 Hz). The D-configuration of the glucosyl residues was confirmed by GC-MS analysis after acid hydrolysis. The linkage of the first glucose moiety to the aglycone was confirmed through HMBC correlations between Hb-1 and C-1′ (δ_C_ 104.7), as well as H-1′ (δ_H_ 4.27, d, *J* = 7.9 Hz) and C-1 (δ_C_ 71.4). The 1,6-interglycosidic linkage was revealed by the downfield shift of C-6′ (δ_C_ 69.9) and further supported by the HMBC correlations from Ha-6′ (δ_H_ 3.78, dd, *J* = 11.6 and 5.5 Hz) to C-1″ (δ_C_ 105.0), as well as H-1″ (δ_H_ 4.37) to C-6′. Thus, compound **9** was identified as 3-(2-hydroxyisopropyl)­pentan-1-ol-1-*O*-[β-d-glucopyranosyl-(1→6)-β-d-glucopyranoside], a previously undescribed dinormonoterpenoid diglucoside that was named dinoside A. The configuration at C-3 was not established.

**Table 3:** ^13^C (125 MHz) and ^1^H (500 MHz) NMR Data of Compounds **9** and **10** (CD_3_OD)^a^

	9				10			
Position	δ_C_	Type	δ_H_	(*J* Hz)	δ_C_	Type	δ_H_	(*J* Hz)
1	71.4	CH_2_	3.59	m	67.4	CH_2_	4.29	m
			3.96	ddd (9.2, 9.2, 5.5)			4.43	dd (12.2, 6.1)
2	31.8	CH_2_	1.46	m	120.4	CH	5.71	
			1.93	dddd (9.1, 6.8, 4.5, 2.1)				
3	49.5	CH	1.26	m	153.1	C		
4	74.6	C			74.4	C		
5	26.4	CH_3_	1.13	s	29.7	CH_3_	1.33	s
6	28.2	CH_3_	1.18	s	29.7	CH_3_	1.33	s
7	25.3	CH_2_	1.15	m	21.9	CH_2_	2.18	dd (7.6, 2.1)
			1.60	ddd (13.4, 7.6, 3.4)			2.20	dd (7.6, 2.44)
8	14.2	CH_3_	0.97	t (7.5, 7.5)	16.0	CH_3_	1.05	t (7.6, 7.6)
1′	104.7	CH	4.27	d (7.9)	103.7	CH	4.32	s
2′	75.2	CH	3.19		75.3	CH	3.21	
3′	78.2	CH	3.49		78.3	CH	3.36	
4′	71.6	CH	3.35		71.9	CH	3.36	
5′	77.2	CH	3.44	m	77.2	CH	3.45	m
6′	69.9	CH_2_	3.78	dd (11.6, 5.5)	70.2	CH_2_	3.81	dd (11.4, 5.7)
			4.15	dd (11.4, 2.0)			4.14	dd (11.4, 2.0)
1″	105.0	CH	4.37	d (7.6)	105.1	CH	4.38	d (7.6)
2″	75.3	CH	3.19	m	75.4	CH	3.21	m
3″	78.1	CH	3.35		78.3	CH	3.36	
4″	71.7	CH	3.28		72.0	CH	3.30	
5″	78.1	CH	3.27		78.2	CH	3.28	
6″	62.9	CH_2_	3.66	dd (11.9, 5.2)	63.1	CH_2_	3.67	dd (11.9, 5.2)
			3.87	dd (11.9, 1.8)			3.88	dd (12.1, 2.0)

a Overlapped signals are reported without multiplicity.

Compound **10** showed the molecular formula C_20_H_36_O_12_, which was determined based on the formate adduct ion at *m*/*z* 513.2200 [M + FA – H]^−^ (calcd for C_21_H_37_O_14_, 513.2189), and further supported by ^13^C and DEPT NMR data (Table 3). While the NMR spectra were very similar to those of **9**, a methine was replaced by a nonprotonated olefinic carbon (δ_C_ 153.1), and a methylene by an olefinic methine (δ_C_ 120.4). This suggested, together with the mass difference of 2 amu compared to **9**, the existence of a double bond between C-2 and C-3. The geometry of the double bond was assigned as *E* based on a ROESY cross peaks between H_2_-1 [δ_H_ 4.29 (m) and 4.43 (dd, *J* = 12.2 and 6.1 Hz)] and Me-8 (δ_H_ 1.05, 3H, t, *J* = 7.6 and 7.6 Hz) as well as the absence of a cross-peak between H-2 (δ_H_ 5.71) and Me-5(6) (δ_H_ 1.33, 6H, s). Therefore, compound **10** is *(E)* -3-(2-hydroxyisopropyl)­pent-2-en-1-ol-1-*O*-[β-d-glucopyranosyl-(1→6)-β-d-glucopyranoside, an unsaturated analogue of **9**, named dinoside B.

Compound **11** had the molecular formula C_26_H_26_O_12_, which was determined based on the protonated molecular ion at *m*/*z* 531.1496 [M + H]^+^ (calcd for C_26_H_27_O_12_, 531.1497) in the HRESIMS spectrum in agreement with the^13^C and DEPT NMR data (Table 4). The ^1^H and^13^ C NMR data suggested a quinic acid derivative substituted by a caffeoyl and a feruloyl residue. The 1,3-disubstitution pattern of the quinic acid residue was inferred from HMBC and COSY correlations (Figure 1) and further confirmed by the excellent agreement of the ^1^H and ^13^C NMR data of the quinic acid moiety with the values reported for cynarine (1,3-dicaffeoylquinic acid).^22^ The caffeoyl residue was located at C-3 based on the HMBC correlation from H-3 (δ_H_ 5.33, m) to C-9′ (δ_C_ 169.0) which in turn indicated the position of the feruloyl group at C-1 (δ_C_ 81.4). In conclusion, compound **11** was identified as 1-*O*-feruloyl-3-*O*-caffeoylquinic acid. This compound (CAS 865095–58–5) is indexed in SciFinder. However, no NMR data are available in the literature, and references (e.g.,^17,18^ ) associated with this compound in the database apparently do not describe 1-*O*-feruloyl-3-*O*-caffeoylquinic acid, but rather the 1,5-disubstituted isomer. This discrepancy may arise from confusion caused by the existence of two different numbering systems for quinic acid derivatives.^16^

**Table 4:** ^13^C (125 MHz) and ^1^H (500 MHz) NMR Data of Compounds **11** and **12** (CD_3_OD)^a^

	11				12			
Position	δ_C_	Type	δ_H_	(*J* Hz)	δ_C_	Type	δ_H_	(*J* Hz)
1	81.4	C			81.3	C		
2	33.1	CH_2_	2.28	dd (16.1, 3.2)	32.8	CH_2_	2.31	dd (16.0, 3.2)
			2.91	dt (16.1, 3.1, 3.1)			2.95	dt (16.0, 3.3, 3.3)
3	73.3	CH	5.33	m	73.3	CH	5.34	m
4	75.5	CH	3.61	m	75.5	CH	3.62	m
5	68.0	CH	4.23	ddd (11.0, 9.6, 4.4)	67.9	CH	4.25	ddd (11.1, 9.7, 4.6)
6	41.4	CH_2_	1.84	dd (13.7, 11.3)	41.7	CH_2_	1.82	dd (13.4, 11.3)
			2.53	m			2.51	m
7	174.9	C			174.7	C		
1′	127.6	C			127.6	C		
2′	116.6	CH	6.47	d (7.9)	116.5	CH	6.42	d (8.2)
3′	146.7	C			146.6	C		
4′	149.4	C			149.4	C		
5′	115.9	CH	6.78	d (2.1)	116.1	CH	6.75	d (1.8)
6′	122.3	CH	6.57	dd (8.2, 1.8)	122.0	CH	6.52	dd (8.4, 2.0)
7′	147.2	CH	7.46	d (16.2)	147.2	CH	7.44	d (15.9)
8′	115.6	CH	6.11	d (15.9)	115.6	CH	6.09	d (15.9)
9′	168.9	C			169.0	C		
1″	127.5	C			126.5	C		
2″	124.4	CH	6.87	dd (8.2, 1.8)	107.0	CH	6.72	s
3″	149.4	C			149.5	C		
4″	150.8	C			139.8	C		
5″	116.5	CH	6.67	d (7.9)	149.5	C		
6″	111.6	CH	6.94	d (1.8)	107.0	CH	6.72	s
7″	147.7	CH	7.51	d (15.9)	148.0	CH	7.52	d (15.9)
8″	115.6	CH	6.23	d (15.9)	116.0	CH	6.28	d (15.9)
9″	168.0	C			167.9	C		
10″	56.4	CH_3_	3.71	s	56.8	CH_3_	3.73	s
11″					56.8	CH_3_	3.73	s

a Overlapped signals are reported without multiplicity.

Compound **12** was assigned the molecular formula C_27_H_28_O_13_, which was determined based on the protonated molecular ion at *m*/*z* 561.1586 [M + H]^+^ (calcd for C_27_H_29_O_13_, 561.1603) in the HRESIMS spectrum and confirmed by ^13^C and DEPT NMR data (Table 4). The NMR spectra showed high similarity to those of compound **11** but revealed the presence of a tetrasubstituted symmetric aromatic ring and a singlet at δ_H_ 3.73 integrating for 6H. This was indicative of the presence of a sinapic acid moiety in place of a feruloyl unit in **11**, in addition to the caffeoyl unit found in both compounds. The location of the caffeoyl moiety at C-3 (δ_C_ 73.3) was established from the HMBC correlation between H-3 (δ_H_ 5.34, m) and C-9′ (δ_C_ 169.0). Consequently, the downfield chemical shift of C-1 (δ_C_ 81.3) revealed the position of attachment of the sinapoyl residue. Thus, compound **12** was identified as 1-*O*-sinapoyl-3-*O*-caffeoylquinic acid.

To complement the analysis of specialized metabolites in the *C. decidua* MeOH extract, feature-based molecular network (FBMN) was generated using HRESIMS data, with the isolated compounds serving as reference standards. Data were further processed with the results of MS2LDA, and SIRIUS analysis (Figure 4). The most prominent cluster consisted predominantly of androstane and pregnane steroids, with a few annotated estrane and ergostane steroids. Compounds **4–8** were located within this cluster, supporting the notion of a steroid cluster. The second most abundant compound class comprised cardenolides, with compounds **1–3** being located in a distinct cluster of related aglycones. Notably, the steroid and cardenolide clusters were connected by a single edge, consistent with the structural similarity between pregnane steroids and cardenolides. Compounds **9** and **10** clustered separately among glycosylated metabolites, reflecting structural similarity to the sugar moieties associated with cardenolides. Compounds **11** and **12**, both cinnamoylquinic ester derivatives, were connected by a single edge but did not cluster with other cinnamic acid derivatives, as their *m*/*z* values suggest they are monomeric phenolic acids. In addition to the isolated compound classes, a cluster of fatty acyls and various amino acid derivatives were annotated, alongside a range of less abundant structurally diverse compounds found predominantly in inactive fractions of the extract (e.g., benzenoids, coumarin derivatives, or prenol lipids that were not investigated further and are grayed out in the network).

**Figure 4:**
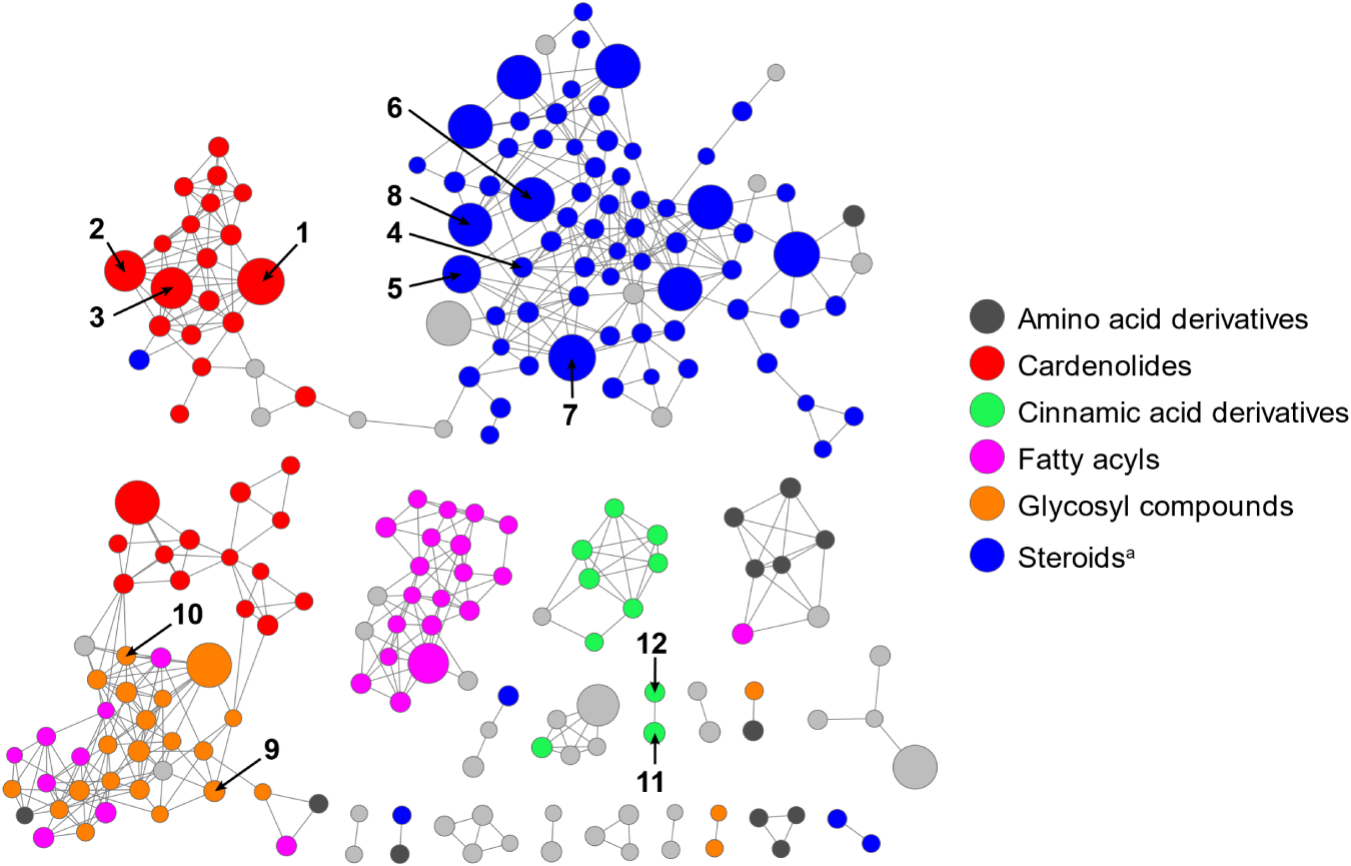
Feature-based molecular network of the crude *C. decidua* MeOH extract with isolated compounds assigned. Node size indicates log_10_-transformed peak area in the extract. Colors indicate manually curated annotations from CANOPUS. Annotations of gray nodes were highly diverse or unreliable. ^a^Including androstane, ergostane, estrane, and pregnane steroids.

Summed up, three cardenolides **1**–**3** featuring a 5β-sarmentogenin-type aglycone were isolated from the roots of *C. decidua*. Notably, compounds **1** and **2** possess an l-oleandrose moiety, which is also present in cryptosin. However, in all cardenolides identified in this study, at least one additional d-glucose unit is present. Given the well-established structural relationship between pregnane steroids and cardenolides,^3^ and the previous report of steroidal compounds in *C. dubia*,^5^ the isolation of sterane derivatives could be expected. Pregnane-type steroids (compounds **6–8**) have previously been reported from other members of the Apocynaceae family, which is consistent with their presence in *C. decidua*.^13,14^ In contrast, androstane steroids such as compounds **4** and **5** were not previously found in this genus. Although animal hormones such as androstenedione are known to occur in plants,^23^ **4** had so-far only been reported as synthetic compound.^15^ The androstane lactone **5** is structurally related to testolactone, a synthetic anticancer agent and a nonselective irreversible aromatase inhibitor.^24^ Such compounds are known as fungal biotransformation products, and have not been previously reported from natural sources. It must be noted that the compound was isolated in very low yield (<0.002% of extract), and attempts to reisolate it from a new batch of plant material were unsuccessful. Future research involving larger amounts of plant material will be required to further explore the biological relevance of this compound. Dinormonoterpenoids structurally related to **9** and **10** have so far only been described in *Cerbera manghas*, another Apocynaceae species.^21^ In contrast, no alkaloids such as cryptolepines, characteristic of *C. sanguinolenta*,^4,25^ were detected even after selective alkaloid extraction and targeted MS detection (data not shown), thereby suggesting a closer chemotaxonomic relationship to *C. dubia*.

To determine the cytotoxic activity of the crude MeOH extract, first the WST-1 assay was used to analyze cell metabolic activity in A549 cells, which was significantly reduced at concentrations greater than 7.5 μg/mL (Figure 5B). To further characterize the cytotoxic effects of *C. decidua* on A549 cells, an apoptosis/necrosis assay was performed with a series of dilutions of the full extract, using Annexin V-FITC as a marker for apoptotic cells and propidium iodide (PI) as a marker for necrotic cells. The DMSO solvent control had no effect, while controls for apoptosis (staurosporine) and necrosis (Triton X) were effective (Figure 5A). A significant increase in apoptosis events could be seen at concentrations 15 μg/mL (190 ± 52% of UT) and 7.5 μg/mL (229 ± 48% of UT) compared to the untreated control (Figure 5A). The effect peaked at 7.5 μg/mL and declined at higher concentrations. At 30 μg/mL, apoptosis rates showed no significant change compared to the untreated control. No significant changes in necrosis rates were observed in any of the samples.

**Figure 5:**
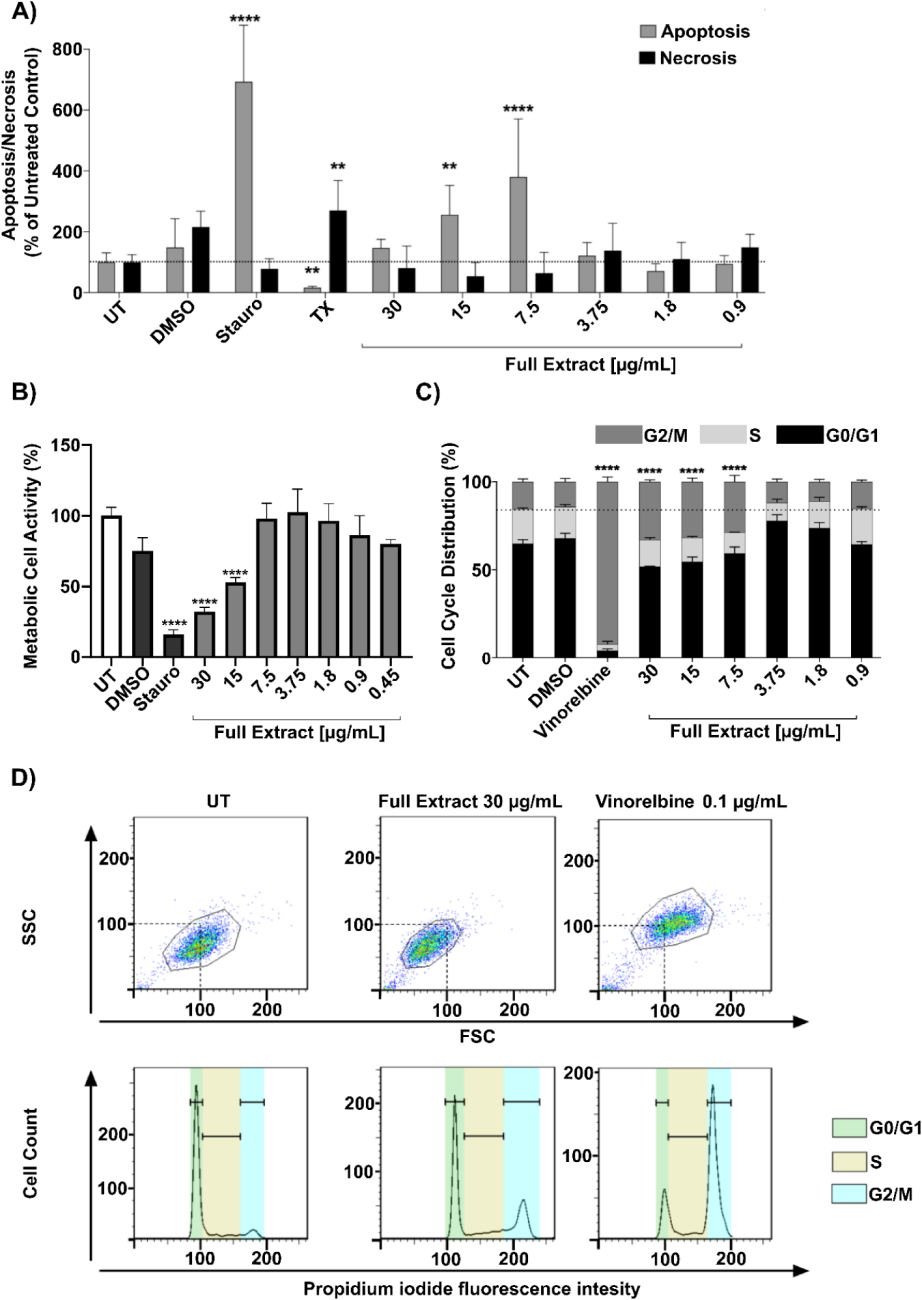
Cytotoxic effects of *C. decidua* whole extract on human adenocarcinoma A549 cell line and human PBMC cells. A) Induction of apoptosis and necrosis in A549 cells treated with full extract, normalized to untreated control; B) Metabolic activity of A549 cells treated with full extract, normalized to untreated control; C) Cell cycle distribution in A549 cells treated with full extract, G2 = G2/M phase, S = S phase, G1 = G0/G1 phase; D) Cell population shift in flow cytometry measurement in A549 cells treated with full extract and vinorelbine, FSC/SSC plot showing shifts in cell size and complexity, SSC = flow cytometer side scatter (cell granularity), FSC = flow cytometer forward scatter (proportional to cell size); UT = untreated control, DMSO = DMSO solvent control, Stauro = staurosporine, TX = Triton X.

Next, the effect of the full extract on the cell cycle of A549 cells was investigated, staining them with PI for DNA content analysis by flow cytometry. A significant increase in the G2/M phase population for concentrations of 30 μg/mL (33.1 ± 1.1% of total cells), 15 μg/mL (31.8 ± 2.1%) and 7.5 μg/mL (28.9 ± 3.6%) was observed compared to an untreated control (15.7 ± 1.6%). Furthermore, the G1 phase was significantly increased at concentrations of 3.75 μg/mL (77.8 ± 3.5%) and 1.875 μg/mL (73.6 ± 3.3%) compared to the untreated control (64.9 ± 2.3%) (Figure 5C). Vinorelbine (0.1 μg/mL), a microtubule-destabilizing agent, served as a control for mitotic cell cycle arrest and could successfully increase the G2/M phase population, while the DMSO solvent control showed no significant impact on cell cycle distribution. Exemplary gating plots of the cell populations indicate a decrease in SSC-H and FSC-H values in the group treated with the full extract compared to the untreated control, while the vinorelbine control shows an increase in cell size and cell granularity. A similar shift is visible for the increase of the propidium iodide fluorescence intensity indicative of an increase in the G2-phase of cells treated with the full extract or vinorelbine (Figure 5D).

For the identification of active constituents, an HPLC-based activity profiling approach was then employed, which combines online spectroscopic data with bioactivity information obtained in parallel by collecting HPLC microfractions.^26^ The crude MeOH extract was separated by HPLC into 35 1-min fractions, which were subsequently tested for their activity. Cell metabolic activity was significantly decreased compared to the untreated control in the main active time window between 8 and 14 min (Figure 6). Two main peaks (*t*_R_ = 10.7 and 11.8 min) were detected in the active window, which were identified as compounds **3** and **2**, respectively. After the preparative isolation, the pure compounds **1–3** were tested for their cytotoxic potential in A549 and PBMC cells, again using the WST-1 metabolic activity assay. Compound **2** with three sugar residues had the lowest cytotoxic activity, with an IC_50_ of 1,860 ng/mL (2.20 μM) in A549 cells and 1,980 ng/mL (2.31 μM) in PBMC cells (Figure 7B). For compound **3** (IC_50_ A549 = 196 ng/mL (0.28 μM); PBMC = 271 ng/mL (0.39 μM)) (Figure 7C) and compound **1** (IC_50_ A549 = 172 ng/mL (0.25 μM); PBMC = 283 ng/mL (0.41 μM)) (Figure 7A) containing both two sugar moieties, inhibitory concentrations were approximately 10-fold lower.

**Figure 6:**
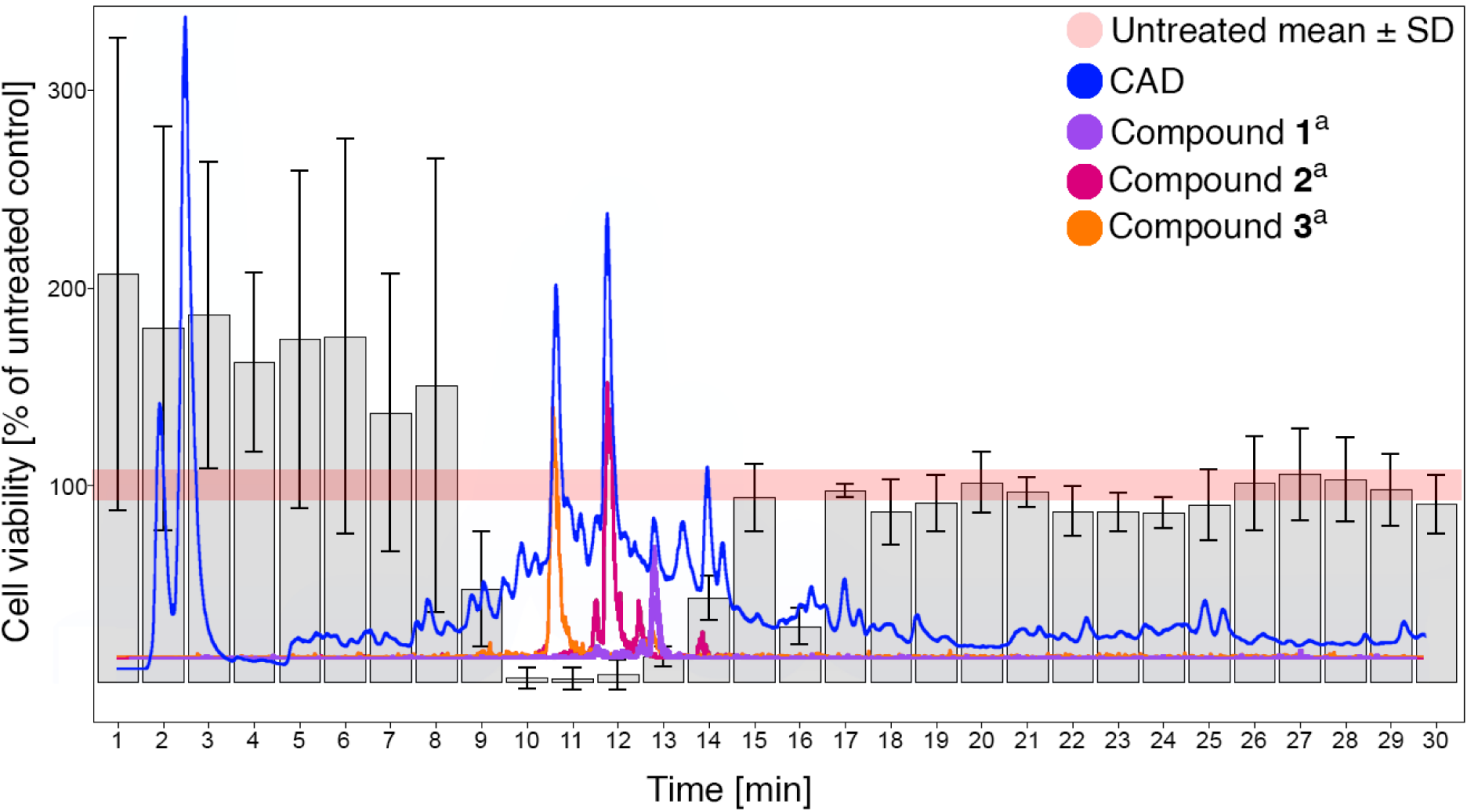
HPLC-based activity profiling of the crude *C. decidua* extract. Microfractions were collected in 1 min steps using a 5–100% B gradient over 30 min. Cytotoxic activity on human adenocarcinoma A549 cells is shown as a bar plot in comparison to the charged aerosol detector (CAD) chromatographic trace. Error bars indicate standard deviation (SD, *n* = 3). ^a^Extracted ion chromatograms of compound **1** (*m*/*z* 741 [M + FA – H]^−^, *t*_R_ = 12.9 min), compound **2** (*m*/*z* 903 [M + FA – H]^−^, *t*_R_ = 11.8 min) and compound **3** (*m*/*z* 743 [M + FA – H]^−^, *t*_R_ = 10.7 min) are shown.

**Figure 7:**
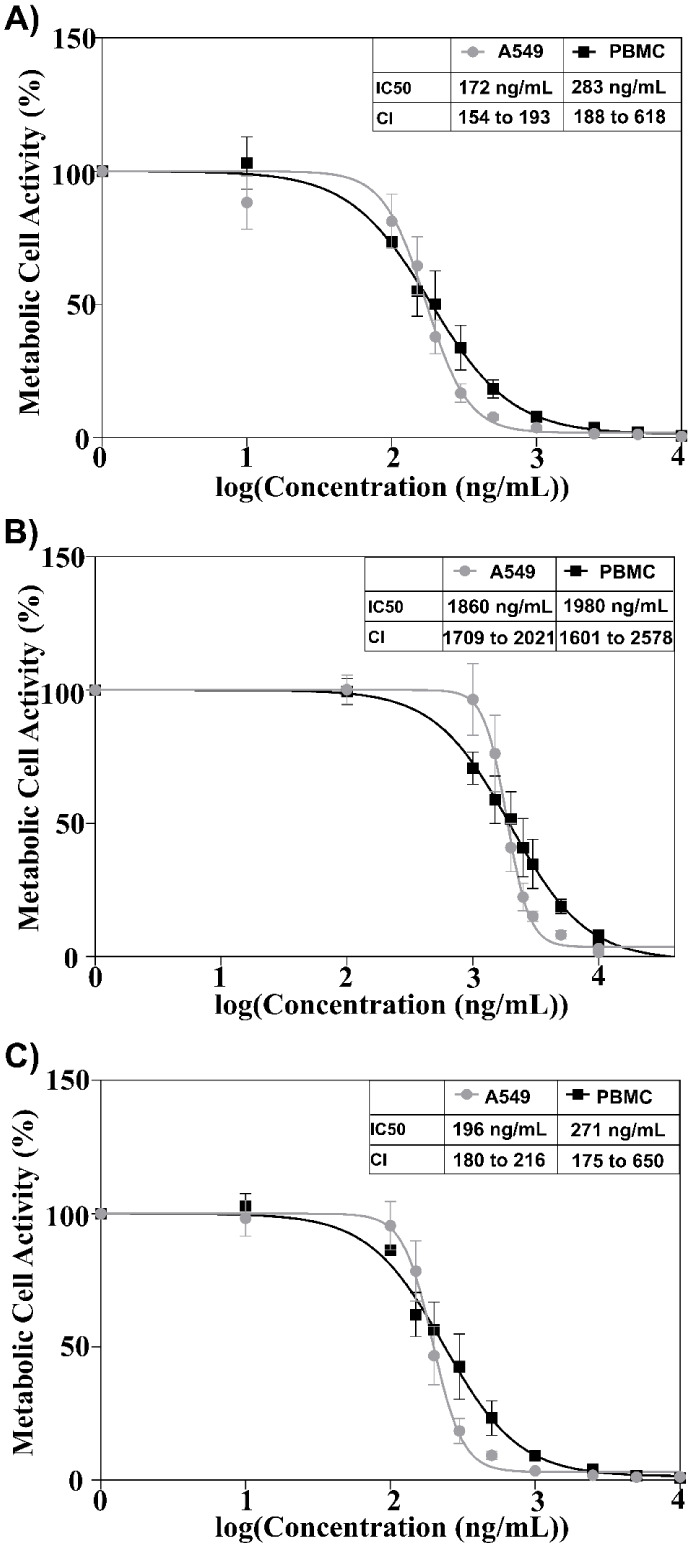
Half-maximal inhibitory concentration (IC_50_) determination in human A549 and PBMC cells for A) compound **1**; B) compound **2**; C) compound **3**; CI = 95% confidence interval in ng/mL.

Cardenolides are known to inhibit the function of the Na^+^/K^+^-ATPase, resulting in cell cycle arrest and apoptosis induction. Although the inhibition of the Na^+^/K^+^-ATPase by the isolated compounds was not tested, the cytotoxic effects reported here for the whole extract are similar to the effects reported for different cardenolides.^27–30^ Cardenolides which are primarily used as cardiac glycosides and have a narrow therapeutic window due to their high toxicity are also being discussed as potential anticancer treatments. The IC_50_ values for primary monocytes in our study were slightly higher than in the A549 cancer cell line, which is a good starting point for the development of new anticancer drugs, especially for radiation-resistant cancers.

In conclusion, a structurally diverse array of phytochemicals was found in *C. decidua*. The comprehensive molecular network highlights the high structural diversity of cardenolides and steroidal compounds in this species. Chemotaxonomically, *C. decidua* aligns closely with *C. dubia*, as cardenolides have been found in both species but not in most other *Cryptolepis* members.^6,8^ Moreover, these species share sterane-type steroids, whereas the indoloquinoline alkaloids of *C. sanguinolenta* appear to be absent.^5^ The observed cytotoxic activity for the methanolic root extract could at least partially, be attributed to the identified cardenolide glycosides. Further studies are warranted to explore the therapeutic potential of this traditional medicinal plant.

## Experimental Section

### General Experimental Procedures

Open column chromatography was conducted using a Sephadex LH-20 column (88 × 5 cm i.d., GE Healthcare, Fairfield, CT, USA), connected to an SP-22 pump (FLOM, Tokyo, Japan), a ProStar 320 UV/vis detector (Agilent Technologies, Santa Clara, CA, USA), and a C660 fraction collector (BÜCHI Labortechnik AG, Flawil, Switzerland). Thin-layer chromatography (TLC) was performed on precoated ALUGRAM Xtra silica gel 60 plates (Merck KGaA, Darmstadt, Germany). Centrifugal partition chromatography (CPC) was carried out on an ARMEN Spot CPC 100 mL (AlphaCrom, Switzerland) equipped with a ProStar 325 UV/vis detector, a ProStar 210 solvent delivery module, and a ProStar 704 fraction collector (Agilent Technologies).

High-resolution electrospray ionization mass spectra (HRESIMS) were recorded on a Q-Exactive HF Orbitrap mass spectrometer (Thermo Fisher Scientific, Waltham, MA, USA), equipped with a HESI-II source, connected to UHPLC system consisting of a 1290 Binary Pump G4220A, 1290 Infinity Autosampler G4226A, 1290 Infinity Thermostat G1130B, 1290 Thermostatted Column Compartment G1316C and 1290 Infinity Diode Array Detector G4212A (all Agilent Technologies). Separation was done using an ACQUITY UPLC BEH C18 column (130Å, 1.7 μm, 150 × 2.1 mm i.d., Waters, Milford, MA, USA), connected to a precolumn of the same phase. Water (A) and MeCN (B), both with 0.1% formic acid (FA), were used as mobile phase with a 0.35 mL/min flow rate.

HPLC-PDA-CAD-ESIMS analyses were performed using an LCMS-8030 chromatographic system, consisting of a triple quadrupole mass spectrometer, two quaternary pumps (LC-20AD), a column oven (CTO-20AC), a diode array detector (SPD-M20A), a degasser (all Shimadzu, Kyoto, Japan), and a Corona Veo RS CAD detector (Thermo Fisher Scientific). Separations were performed on a SunFire C18 column (3.5 μm, 150 × 3 mm i.d., Waters), equipped with a guard column (10 × 3.0 mm i.d.). Solvents A and B were used as mobile phase with a 0.4 mL/min flow rate. LabSolutions software (Shimadzu) was used for data acquisition and processing.

Semipreparative HPLC separations were conducted using an Agilent HP 1100 Series system equipped with a binary pump (G1312A BinPump), an autosampler (G1367A WPALS), a column oven (G1316A COLCOM), and a diode array detector (G1315A DAD, all Agilent Technologies) (System 1) or, for compounds devoid of a UV chromophore, an Agilent Technologies system comprising a binary pump (1260 Prep Bin Pump), a diode array detector (G1315B DAD), and an ESIMS with a single quadrupole MS detector (6120, all Agilent Technologies) (System 2). In System 2, a Quicksplit adjustable nano flow splitter (Analytical Scientific Instruments Inc., Richmond, CA, USA) provided a 1:100 flow split, and a makeup flow of 0.4 mL/min (100% MeCN with 0.1% FA) was added after the split using a quaternary pump (Agilent 1290 Infinity II). Chromatographic separations on both systems were performed either on a SunFire C18 column (3.5 μm, 150 × 10 mm i.d., Waters), a ReproSIL-Pur 120 C18-AQ column (3 μm, 150 × 10 mm i.d., Dr. Maisch GmbH, Ammerbuch-Entringen, Germany), or a Kinetex Biphenyl column (5 μm, 250 × 10 mm i.d., Phenomenex Ltd., Aschaffenburg, Germany), each equipped with a corresponding guard column (10 × 10 mm i.d.). A flow rate of 4 mL/min was used if not otherwise indicated. Preparative HPLC was performed on System 2 using a SunFire Prep C18 OBD column (5 μm, 150 × 30 mm i.d., Waters) combined with a C18 Prep Guard Cartridge (10 × 30 mm i.d.). A 20 mL/min flow rate was applied. ChemStation software (Agilent Technologies) was used for data acquisition and processing.

NMR data were recorded on a Bruker Avance III spectrometer with a 5 mm BBO probe at 23 °C (Bruker, Fällanden, Switzerland) at 500 MHz (^1^H) and 125 MHz (^13^C), respectively. Spectra were measured in CD_3_OD (ARMAR Chemicals, Döttingen, Switzerland). Chemical shifts are reported in parts per million (δ) using the solvent signal (δ_H_ 3.31; δ_C_ 49.15) as internal reference; coupling constants (*J*) are given in Hz. Bruker Topspin 3.5 software was used for data acquisition and ACD/Laboratories NMR Workbook suites (Advanced Chemistry Development, Inc., Toronto, ON, Canada) for data processing.

Optical rotations were recorded on a JASCO P-2000 polarimeter (Portmann Instruments AG, Biel-Benken, Switzerland) combined with a 10 cm temperature-controlled microcell at 25 °C and a wavelength of 589 nm (Na-D line). Samples were dissolved in HPLC-grade MeOH at a concentration of 1 mg/mL and 30 measurements were averaged. Specific optical rotation values are given in deg mL g^–1^ dm^–1^. UV spectra (220–450 nm, scan speed 400 nm/min) were recorded in 1 mL quartz microcuvettes with 1 cm path length (Hellma GmbH, Müllheim, Germany) at 0.5 nm band on a JASCO V-650 spectrophotometer (Portmann Instruments) at room temperature. IR spectra were recorded on a PerkinElmer Spectrum Two FT-IR spectrometer (PerkinElmer, Shelton, USA).

Water was from a Milli-Q water purification system (Merck Millipore, Billerica, USA). HPLC-grade MeCN (VWR International GmbH, Darmstadt, Germany), and formic acid (Biosolve BV, Valkenswaard, Netherlands) were used for HPLC. Chloroform, DMSO, and methanol were from Reuss-Chemie AG (Tägerig, Switzerland).

### Plant Material and Extraction

The roots of *Cryptolepis decidua* (Planch. ex Benth.) N. E. Br. were collected by one of the authors (RH) in April 2023 and February 2025 on semiarid mountain slopes, in the Kunene region, Namibia (Voucher: CID55, National Botanical Research Institute of Namibia (NBRI)). The collection process involved interviewing local residents of the Himba group with the assistance of a translator. The root material was identified by one of the authors (RH) and local residents, harvested, chopped up with an axe, and then bundled up by local residents. The material was dried in shade at the collection site. Upon arrival in Germany, around 10 days after collection, the dry roots were stored at −20 °C until further processing.

For extraction, dried *C. decidua* roots (297 g) were milled with liquid nitrogen using a ZM1 centrifugation mill (Retsch, Haan, Germany). The powder was extracted with MeOH (24, 24, and 72 h, 1′000 mL each) under stirring at room temperature. After filtration the extracts were combined, evaporated under reduced pressure and finally freeze-dried to yield the crude extract (26.4 g).

### HPLC-MS and MS^2/3^ Analyses of Cardenolides **1–3**

The crude MeOH extract (1.0 mg) was dissolved in MeOH (1.0 mL). For analysis, this solution (100 μL) was centrifuged (10 min, 20000 × *g*, 4 °C). The supernatant (80.0 μL) was collected for analysis, and the precipitate was discarded. Chromatographic analyses were performed on an Agilent 1100 HPLC system (Agilent Technologies, Waldbronn, Germany) equipped with a SunFire C18 column (3.5 μm, 150 × 3 mm i.d., Waters), equipped with a guard column (10 × 3.0 mm i.d.). Water (A) and MeCN (B), both containing 0.1% FA were used as mobile phase with a 0.4 mL/min flow rate. The following gradient was applied: 0 min, 5% B; 0–30 min, 5–100% B; 30–35 min, 100% B; 35–36 min, 100–5% B; 36–45 min, 5% B. The column temperature was kept at 40 °C. The sample volume was 2 μL in each experiment.

The MS and MS^2/3^ experiments were performed using an LTQ Orbitrap XL (Thermo Fisher Scientific, Bremen, Germany) in positive or negative electrospray ionization mode with a mass resolution of 60000. The full-scan data were collected between *m*/*z* 100 and 1200 in profile mode. The following MS parameters in positive mode were applied: capillary temperature, 275 °C; sheath gas flow, 12 L/min; aux gas flow, 3 L/min; sweep gas flow, 0 L/min; source voltage, 4 kV; capillary voltage, 43 V; tube lens, 125 V. The following MS parameters in negative mode were applied: capillary temperature, 275 °C; sheath gas flow, 25 L/min; aux gas flow, 7 L/min; sweep gas flow, 0 L/min; source voltage, 3.5 kV; capillary voltage, −45 V; tube lens, −110 V. The following MS^2/3^ parameters were applied: activation type, CID; isolation width, *m*/*z* 1.0; normalized collision energy, 40 V; activation Q, 0.25; activation time, 30 ms. Helium was used as collision gas. Thermo Tune Plus was used for data acquisition, Thermo Xcalibur for processing, MZmine 3.8.0 ^31^ and Inkscape 1.2.1 for data presentation.

### Compound Isolation

A portion (11.5 g) of the MeOH extract was dissolved in 40 mL MeOH. After centrifugation (18 × *g*, 7 min), the supernatant was loaded onto a Sephadex LH-20 column eluted with MeOH (2 mL/min). A total of 205 fractions were collected in 5 min-steps, and combined based on their TLC profiles into 14 main fractions (A1-N1). This procedure was repeated with the remaining extract (14.5 g), yielding another 156 fractions which were combined into 13 main fractions (A2-M2).

Fractions D1 (300 mg), F1 (635 mg), and G1 (2′000 mg) were subjected to CPC using a methanol/chloroform/water 9:12:8 (v/v/v) biphasic solvent system, adapted from Butler et al.^32^ The separation was performed in ascending mode with the upper aqueous phase as mobile phase at 3 mL/min and 1′600 rpm. Each sample was dissolved in 10 mL of a 1:1 (v/v) mixture of both phases before injection. Fractions were collected at 2 min intervals. Once the effluent appeared analyte-free, the flow direction was switched to the descending mode. From Fraction D1, 20 subfractions were collected in ascending mode and 16 in descending mode which were pooled into 10 fractions based on TLC analysis. HPLC-MS analysis indicated overlapping major peaks in Fractions 2–4, which were recombined (108 mg) and purified by semipreparative HPLC (System 1) on a SunFire C18 column with 25–43% B (see 1.2) over 20 min, yielding compound **2** (18.5 mg *t*_R_ = 5.4 min). From Fraction F1, 16 subfractions were collected in both modes and combined into 8 pooled fractions based on TLC. Fraction 1 (100 mg) was subjected to semipreparative HPLC-ESIMS (System 2, SunFire C18 column, 10–18% B over 20 min) to yield compound **10** (1.8 mg, detection at *m*/*z* 513.2 [M + FA – H]^−^, *t*_R_ = 9.1 min). Fractions 2 and 3 were combined (178 mg) and separated by semipreparative HPLC (System 1, SunFire C18 column, 20–27% B over 20 min) to afford crude compound **3** (42 mg). Fraction G1 was similarly processed by CPC, yielding 11 fractions. Pooled Fractions 2 and 3 (82 mg) were further purified using by semipreparative HPLC (System 2, SunFire C18, 18–25% B over 20 min), yielding an additional amount of **3** (1.9 mg, detection at *m*/*z* 743 [M + FA – H]^−^, *t*_R_ = 9.6 min). Both samples of compound **3** were pooled and finally purified (System 1, SunFire C18, 18–25% B over 15 min) to give pure compound **3** (37 mg, *t*_R_ = 8.6 min). Fraction C2 (600 mg) was separated by preparative HPLC (SunFire C18, 45–58% MeOH (+0.1% FA) over 15 min). Two peaks were collected (*t*_R_ = 6.08 and 7.47 min) with detection at *m*/*z* 513.3 [M + FA – H]^−^ and *m*/*z* 515.3 [M + FA – H]^−^, respectively. The previously isolated peaks were recombined and purified by semipreparative HPLC (System 2, ReproSIL-Pur 120 C18-AQ, 11–14% B over 15 min), yielding compound **9** (4.3 mg, detection at *m*/*z* 515.3 [M + FA – H]^−^, *t*_R_ = 9.49 min). Fraction D2 (1.1 g) was separated via preparative HPLC (20–32% B over 20 min) followed by semipreparative HPLC (System 2, ReproSIL-Pur 120 C18-AQ column, 60–75% MeOH (+0.1% FA) over 20 min, 3 mL/min) to afford compound **1** (7.5 mg, *t*_R_ = 7.6 min). Fraction H (1.2 g) was separated by preparative HPLC (36–43% B, 20 min) to give compound **7** (21 mg, *t*_R_ = 12.3 min) and further peaks which were finally purified by semipreparative HPLC (System 1, ReproSIL-Pur 120 C18-AQ column). Compound **4** (3.0 mg, *t*_R_ = 6.6 min) was isolated using 65–71% MeOH (+0.1% FA) over 15 min; compound **5** (0.44 mg, *t*_R_ = 8.3 min) using 34–35% B over 20 min; compound **6** (1.0 mg, *t*_R_ = 15.4 min) using 57–70% MeOH (+0.1% FA) over 20 min; and compound **8** (4.5 mg, *t*_R_ = 18.0 min) using 39–40% B over 20 min. Fraction K2 (300 mg) was separated by semipreparative HPLC (System 2, Kinetex Biphenyl column, 19% B isocratic over 15 min) to yield compounds **11** (2.5 mg, detection at *m*/*z* 529 [M – H]^−^, *t*_R_ = 9.0 min) and **12** (10.6 mg, detection at *m*/*z* 559 [M – H]^−^, *t*_R_ = 8.2 min).

#### 5β-Sarmentogenin 3-*O*-[β-d-Glucopyranosyl-(1→6)-β-d-glucopyranosyl-(1→4)-α-l-oleandroside] (**2**)

White amorphous powder; [α]^25^ d −47.5 (c 0.1, MeOH); UV (MeOH) λ_max_ (log ε) 218 (4.10) nm; ^1^H NMR (500 MHz, CD_3_OD) and ^13^C NMR (125 MHz, CD_3_OD), see Table 1; HRESIMS *m*/*z* 903.4273 [M + FA – H]^−^ (calcd for C_43_H_67_O_20_, 903.4231).

#### 5β-Sarmentogenin 3-*O*-[β-d-Glucopyranosyl-(1→4)-α-l-rhamnopyranoside] (**3**)

White amorphous powder; [α]^25^ d −30.7 (c 0.1, MeOH); UV (MeOH) λ_max_ (log ε) 218 (4.09) nm; IR (KBr) 3416, 2934, 1737, 1625, 1066 cm^–1^; ^1^H NMR (500 MHz, CD_3_OD) and ^13^C NMR (125 MHz, CD_3_OD), see Table 1; HRESIMS *m*/*z* 743.3531 [M + FA – H]^−^ (calcd for C_36_H_55_O_16_, 743.3496).

#### 2α-Hydroxy-3,17-dioxo-androsta-4,6-diene (**4**)

White amorphous powder; [α]^25^ d +136 (c 0.1, MeOH); UV (MeOH) λ_max_ (log ε) 202 (3.65), 282 (4.27) nm; ^1^H NMR (500 MHz, CD_3_OD) and ^13^C NMR (125 MHz, CD_3_OD), see Table 2; HRESIMS *m*/*z* 301.1793 [M + H]^+^ (calcd for C_19_H_25_O_3_, 301.1798).

#### 2α-Hydroxy-d-homo-17a-oxaandrosta-4,6-diene-3,17-dione (**5**)

White amorphous powder; UV (MeOH) λ_max_ (log ε) 202 (3.42), 281 (3.86) nm; ^1^H NMR (500 MHz, CD_3_OD) and ^13^C NMR (125 MHz, CD_3_OD), see Table 2; HRESIMS *m*/*z* 317.1744 [M + H]^+^ (calcd for C_19_H_25_O_4_, 317.1747).

#### Dinoside A (3-(Hydroxyisopropyl)­pentan-1-ol-1-*O*-[β-d-glucopyranosyl-(1→6)-d-glucopyranoside]) (**9**)

White amorphous powder; [α]^25^ d −41.9 (c 0.1, MeOH); UV (MeOH) λ_max_ (log ε) 203 (3.41) nm; ^1^H NMR (500 MHz, CD_3_OD) and ^13^C NMR (125 MHz, CD_3_OD), see Table 3; HRESIMS *m*/*z* 515.2362 [M + FA – H]^−^ (calcd for C_21_H_39_O_14_, 515.2345).

#### Dinoside B (*E*)-3-(Hydroxyisopropyl)­pent-2-en-1-ol-1-*O*-[β-d-glucopyranosyl-(1→6)-d-glucopyranoside] (**10**)

White amorphous powder; [α]^25^ d −29.8 (c 0.1, MeOH); UV (MeOH) λ_max_ (log ε) 204 (3.67), 278 (2.71) nm; ^1^H NMR (500 MHz, CD_3_OD) and ^13^C NMR (125 MHz, CD_3_OD), see Table 3; HRESIMS *m*/*z* 513.2200 [M + FA – H]^−^ (calcd for C_21_H_37_O_14_, 513.2189).

#### 1-*O*-Feruloyl-3-*O*-caffeoylquinic Acid (**11**)

Yellow amorphous powder; ^1^H NMR (500 MHz, CD_3_OD) and ^13^C NMR (125 MHz, CD_3_OD), see Table 4; HRESIMS *m*/*z* 531.1496 [M + H]^+^ (calcd for C_26_H_27_O_12_, 531.1497).

#### 1-*O*-Sinapoyl-3-*O*-caffeoylquinic Acid (**12**)

Yellow amorphous powder; [α]^25^ d −28.5 (c 0.1, MeOH); UV (MeOH) λ_max_ (log ε) 202 (4.43), 221 (4.38), 241 (4.38), 322 (4.48) nm; IR (KBr) 3424, 2941, 1696, 1605, 1516, 1284, 1108 cm^–1^; ^1^H NMR (500 MHz, CD_3_OD) and ^13^C NMR (125 MHz, CD_3_OD), see Table 4; HRESIMS *m*/*z* 561.1586 [M + H]^+^ (calcd for C_27_H_29_O_13_, 561.1603).

### Acid Hydrolysis and Sugar Analysis

Each of the compounds **1**–**3**, **9**, and **10** (0.5–2.6 mg) were hydrolyzed with 2 M HCl (0.5 mL) for 15 h at 100 °C. The hydrolyzate was extracted with ethyl acetate (EtOAc, 2 × 0.5 mL). The aqueous phase was dried under nitrogen and then under high vacuum. The residue was treated with 250 μL of a mixture of (+)-2-butanol and acetyl chloride 10:0.5 (v/v) at 55 °C for 15 h. After drying, the residue was further derivatized with 250 μL of a mixture of trifluoroacetic anhydride (TFAA) and EtOAc 3:4 (v/v) at 55 °C for 1 h. d-Glucose, and l-rhamnose were treated following the same procedure. l-Oleandrose is not commercially available and attempts to obtain it by hydrolysis of a commercial sample of oleandrin (MCE MedChemExpress, Monmouth Junction, NJ, USA) remained unsuccessful. GC-MS analysis was performed as previously described^33^ on a J&W DB-225 ms GC column (30 m; 0.25 mm i.d.; film thickness 0.25 μm; Agilent Technologies) with a slightly modified temperature program: 60 °C hold for 1 min, increase to 240 °C at 7 °C/min followed by 5 min at 240 °C. d-Glucose (*t*_R_ = 15.04/17.04 min) was identified in the hydrolyzate of compounds **1**-**3**, **9**, and **10** while l-rhamnose (*t*_R_ = 10.39 min) was additionally detected in the hydrolyzate of **3**. The two peaks observed for d-glucose are due to the anomeric forms after derivatization.

### Feature-Based Molecular Networking

An aliquot of the crude MeOH extract of *C. decidua* and compounds **1**–**12** were dissolved in MeOH to a concentration of 3 mg/mL and 100 μg/mL, respectively. The gradient was optimized to a 5% B hold for 0.5 min, followed by 5–100% B over 15 min, a wash at 100% B for 2.5 min, and a final equilibration at 5% B for 3 min. Between 1 and 3 μL of sample were injected into the system for HRESIMS measurements. The ion source of the Orbitrap was set to 3.5 kV with the sheath gas set to 55 (arb), auxiliary gas set to 15 (arb) and sweep gas set to 0 (arb). Temperatures of the auxiliary gas was set to 250 °C and the ion transfer capillary was set to 350 °C. Resolution of the mass spectrometer was set to 45,000 with a scan range of 100–1500 *m*/*z*. Maximum injection time was 50 ms and the AGC target 2e5. For MS/MS data acquisition, the data dependent acquisition mode was used. Resolution was set to 15,000. The AGC target was 5e4, loop count was three and maximum injection time was 50 ms. The MS/MS isolation window was set to 1 *m*/*z* and fragmentation of the top three most intense signals was triggered at an intensity threshold of 2e1. HCD fragmentation occurred with a stepped normalized collision energy consisting of 15, 30, and 45 units. Isotopes were excluded from fragmentation and signals subjected to fragmentation were put on an exclusion list for 5 s.

Feature-based molecular networking was conducted using the GNPS1 platform.^34^ Raw MS data files were converted to the mzML format using MSConvert (v3.0.25030–393db81).^35^ Feature detection and alignment were carried out in MZmine (v4.5.20).^36^ Parameters included a maximum of 12 peaks per chromatogram, a minimum of 4 consecutive scans, and an approximate feature width at half maximum (FWHM) of 0.05 min. The noise threshold was set with the factor of lowest signal being 18.00 (MS1) and 2.00 (MS2). Minimum feature height was set to 2 × 10^6^ units. All other settings were kept at default, as declared in the mzwizard module. Feature lists corresponding to the isolated compounds were manually curated to retain only one representative feature per compound. These curated lists were aligned with the extract data to generate an .mgf file, a quantification table, and network edge data. The resulting files were submitted to the GNPS FBMN workflow (v28.2) with a precursor/fragment ion mass tolerance of 0.02 Da and a minimum cosine score of 0.6. To enhance the molecular network, MS2LDA analysis was performed via the respective GNPS workflow (v31.1), using the MGF file, quantification table, and GNPS-derived network pairs as input. MS2LDA detects recurrent fragment and neutral loss patterns (Mass2Motifs), providing insights into shared substructures.^37^ For computation of molecular formulas, MS/MS based structural predictions, as well as compound classification SIRIUS (v6.1.1) – utilizing ZODIAC and CANOPUS – was used.^38–40^ The SIRIUS-derived annotations were integrated with the MS2LDA substructure network and the MZmine quantification data in Cytoscape (v3.10.3).^41^ Annotations were selectively chosen among the different classifications computed by CANOPUS, based on their consistency with the structural information obtained from the isolated compounds.

### Extraction for Cytotoxicity Screening and HPLC-Based Activity Profiling

Dried roots of *C. decidua* (3 g) were extracted with MeOH by pressurized liquid extraction (Dionex ASE 350, Thermo Fisher Scientific) to yield, after evaporation to dryness, 426 mg of extract.

Microfractionations were performed with the HPLC-PDA-CAD-ESIMS system described above on a SunFire C18 column. An aliquot of the extract dissolved in DMSO at a concentration of 10 mg/mL. H_2_O (A) and MeCN (B) both containing 0.1% FA were used as mobile phase and a gradient of 5–100% B in 30 min was applied. The HPLC flow was diverted after the UV detector into a microfraction collector (FC 204 Gilson, USA). One-minute fractions were collected into a 96-deep well plate. Three runs with each 30 μL injection volume were performed and the microfractions from the three runs were collected in the same wells. One additional run with 10 μL was performed without collection but with the HPLC additionally connected to the CAD and MS detectors. After the collection, the plates were dried in a centrifuge vacuum evaporator (EZ-2 Plus Genevac LTD, UK). For cytotoxicity testing, the microfractions were redissolved in 20 μL DMSO.

### Cytotoxic Assays and Cell Cultivation

A549 human epithelial lung cancer cells were cultivated in Dulbecco’s Modified Essential Medium (DMEM, with 4.5 g/L glucose, 1% l-glutamine, without sodium pyruvate; Gibco by Thermo Fisher Scientific) supplemented with 10% FCS, 1% penicillin and 1% streptomycin. PBMCs were isolated from blood donations of healthy adults at the University Medical Center Freiburg, as previously described.^42,43^ Briefly, buffy coats, were processed using Ficoll density gradient centrifugation to separate PBMCs from other blood components. Cells were then cultured in Roswell Park Memorial Institute 1640 medium (RPMI; Gibco by Thermo Fischer Scientific) supplemented with 10% fetal calf serum (FCS) (anprotec, Bruckberg, Germany), 2 mM l-glutamine (Gibco), 100 U/mL penicillin, 100 U/mL streptomycin (Sigma-Aldrich, St. Louis, USA) and 0.025% 2-mercaptoethanol (Sigma-Aldrich). Cells were simultaneously seeded, treated with different concentrations of extracts and stimulated to ensure T-cell functionality. Stimulation was achieved using human Immunocult CD3/CD28 T cell activator (25 μL/mL) (STEMCELL Technologies, Cologne, Germany) alongside a nonstimulated control. All cells were maintained at 37 °C in a humidified incubator with a 5% CO_2_/95% air atmosphere.

### Cell Viability (WST-1) Assay of A549 Cells and PBMCs

WST-1 assay was conducted to assess the cytotoxic effects of *C. decidua*. A549 cells were seeded at a density of 2.5 × 10^5^ cells/mL for a 24-h treatment and 1 x10^5^/mL for a 72-h treatment. PBMCs were seeded at a density of 2 × 10^6^ cells/mL for a 72-h treatment. The WST assay was performed using 5% WST-1 solution (Roche, Indianapolis, USA) in culture medium w/o phenol red. A549 cells were incubated for 10 min and PBMCs for 2–3 h before absorbance was measured with a plate reader (Tecan iControl) at 440 nm, using 690 nm as a reference wavelength. Controls included staurosporine (10 μM; Sigma-Aldrich) as a proliferation inhibitor and DMSO (1%) as a solvent control.

### Analysis of Apoptosis and Necrosis in A549 Cells Using Annexin V and Propidium Iodide Staining

Apoptosis/necrosis assay was performed with A549 cells which were seeded at a density of 2.5 × 10^5^ cells/mL and treated with plant extracts after 24 h. The assay was conducted 24 h post-treatment using the Annexin V FITC apoptosis detection kit (eBioscience, Frankfurt, Germany) according to the manufacturer’s instructions. Staurosporine (5 μg/mL) served as a positive control for apoptosis, Triton X-100 (0.47%; Sigma-Aldrich) as a positive control for necrosis, and DMSO (1%) as a solvent control. Apoptotic and necrotic events were immediately analyzed by flow cytometry after staining.

### Cell Cycle Analysis in A549 Cells Using Propidium Iodide Staining

Cell cycle analysis was performed using propidium iodide (PI) DNA content staining to determine the distribution of A549 cells in different cell cycle phases, G0/G1, S, and G2/M after treatment with the extracts. A549 cells were seeded at a density of 2.5 × 10^5^ cells/mL and treated with extracts after 24 h. Twenty-4 h after treatment, the cells were fixed in 70% ethanol at −20 °C for at least 1 h to allow membrane permeabilization. Cells were then stained using a solution containing 40 μg/mL PI (Invitrogen by Thermo Fisher Scientific) and 10 μg/mL RNase A (Thermo Fisher Scientific) and incubated for 30 min in the dark at room temperature. Vinorelbine (100 ng/mL) served as a control for cell cycle arrest in the G2/M phase, DMSO 1% as a solvent control. Fluorescence was measured using flow cytometry, where PI was excited with a 488 nm laser and detected using a 575/26 bandpass filter. The collected data were analyzed with FlowJo (version 7.6.5) software, applying a consistent gating strategy across all experiments. The Watson-Pragmatic model was used to define the G1 and G2 peaks, ensuring reproducible results.

### Data Analysis

Biological assays were conducted in at least three independent experiments with three different generations of cells. Data analysis was performed using GraphPad Prism (version 10.2.3). Descriptive statistics were applied, and outliers were removed using the ROUT method. Samples were normalized to their respective controls before comparison. Statistical significance was determined using one-way ANOVA, followed by Dunnett’s post hoc pairwise comparisons. If disturbing variance persisted after removing outliers, the data were transformed to (Y = √Y) prior to conducting the one-way ANOVA. IC_50_ values were determined by fitting the dose–response data to a variable slope (four parameters) model using GraphPad Prism, where inhibitor concentration (log scale) was plotted against the biological response to calculate the half-maximal inhibitory concentration (IC_50_). Results are presented as mean ± SD for the specified number of independent experiments. Asterisks indicate significant differences from controls (*p < 0.05; **p < 0.01; ***p < 0.001; ****p < 0.0001).

## Supplementary Material



## Data Availability

The raw NMR data for compounds **1–12** have been deposited in the Natural Products Magnetic Resonance Database (https://np-mrd.org/) under accession numbers NP0352086 (**1**), NP0352087 (**2**), NP0352088 (**3**), NP0352089 (**4**), NP0352090 (**5**), NP0352096 (**6**), NP0352085 (**7**), NP0352091 (**8**), NP0352092 (**9**), NP0352093 (**10**), NP0352094 (**11**), and NP0352095 (**12**).

## Notes

LC-MS^1/2/3^ data and IC_50_ analysis (Hill coefficient h and R^2^ values) of compounds **1**–**3**, NMR data as well as 1D/2D NMR spectra of **1**–**12**, IR spectra of **3** and **12**, and GC-MS analysis of sugars in **1**–**3**, **9**, and **10** (PDF)
