# Supplementary material for: Comprehensive Metabolite Profile and Cytotoxic Constituents of Cryptolepis decidua
Source: J Nat Prod. 2026 Apr 24;89(5):1462–77. doi: 10.1021/acs.jnatprod.6c00033 (PMC13200256; doi:10.1021/acs.jnatprod.6c00033)
Supplement: Supplementary file 1 [file np6c00033_si_001.pdf]

Supporting Information

Comprehensive Metabolite Profile and Cytotoxic Constituents of *Cryptolepis decidua*

Tobias Blank[1]‡, Charlie Puth[2]‡, Annikka Kurz[2], Moritz Benka[3,4], Malik Rakhmanov[1], Iwanette Du Preez-Bruwer[5], Davis Mumbengegwi[5], Dietmar A. Plattner[4], Bernd Kammerer[3,4], Roman Huber[2], Robin Teufel[1], Olivier Potterat[1*], Volker M. Lüth[2*]

‡T.B. and C.P. contributed equally.

[1]Pharmaceutical Biology, Department of Pharmaceutical Sciences, University of Basel, 4056 Basel, Switzerland

[2]Centre for Complementary Medicine, Department of Internal Medicine II, Faculty of Medicine, University of Freiburg, 79106 Freiburg, Germany

[3]Core Competence Metabolomics, Hilde Mangold Haus, University of Freiburg, 79104 Freiburg, Germany

[4]Institute of Organic Chemistry, University of Freiburg, 79104 Freiburg, Germany

[5]Multidisciplinary Research Centre, University of Namibia, 340 Mandume Ndemufayo Avenue, Pioneers Park, Windhoek, Namibia

Corresponding authors:

Olivier Potterat - Email: Olivier.Potterat@unibas.ch

Volker M. Lüth - Email: Volker.Lueth@uniklinik-freiburg.de

Table S1: LC-MS[1] data in positive and negative mode... 4
Table S2: LC-MS[2/3] data in negative mode...5
Table S3: 13C (125 MHz) and 1H (500 MHz) NMR data of compound **1** (CD_3OD). 6
Table S4: ^{13}C (125 MHz), ^{1}H, ^{1}H-^{1}H COSY, ^{1}H-^{13}C HMBC, and ^{1}H-^{1}H ROESY (500 MHz) NMR data of compound **2** (CD_3OD)..7
Table S5: ^{13}C (125 MHz), ^{1}H, ^{1}H-^{1}H COSY, ^{1}H-^{13}C HMBC, and ^{1}H-^{1}H ROESY (500 MHz) NMR data of compound **3** (CD_3OD)..8
Table S6: ^{13}C (125 MHz), ^{1}H, ^{1}H-^{1}H COSY, ^{1}H-^{13}C HMBC, and ^{1}H-^{1}H ROESY (500 MHz) NMR data of compound **4** (CD_3OD)..9
Table S7: ^{13}C (125 MHz), ^{1}H, ^{1}H-^{1}H COSY, ^{1}H-^{13}C HMBC, and ^{1}H-^{1}H ROESY (500 MHz) NMR data of compound **5** (CD_3OD)..10
Table S8: ^{1}H (500 MHz) NMR data of compounds **6-8** (CD_3OD)..11
Table S9: ^{13}C (125 MHz) NMR data of compounds **6-8** (CD_3OD). 12
Table S10: ^{13}C (125 MHz), ^{1}H, ^{1}H-^{1}H COSY, and ^{1}H-^{13}C HMBC (500 MHz) NMR data of compound **9** (CD_3OD). 13
Table S11: ^{13}C (125 MHz), ^{1}H, ^{1}H-^{1}H COSY, ^{1}H-^{13}C HMBC, and ^{1}H-^{1}H ROESY (500 MHz) NMR data of compound **10** (CD_3OD)..14
Table S12: ^{13}C (125 MHz), ^{1}H, ^{1}H-^{1}H COSY, and ^{1}H-^{13}C HMBC (500 MHz) NMR data of compound **11** (CD_3OD). 15
Table S13: ^{13}C (125 MHz), ^{1}H, ^{1}H-^{1}H COSY, ^{1}H-^{13}C HMBC, and ^{1}H-^{1}H ROESY (500 MHz) NMR data of compound **12** (CD_3OD)..16
Table S13: Hill coefficient (h) and goodness of fit (R^2) for human A549 and PBMC cells treated with compound 1,2 and 3 to analyze half-maximal inhibitory concentrations (IC_{50})...17
Figure S1. Excerpt of the TIC of the MeOH extract in negative mode 17
Figure S2. ^{1}H NMR spectrum of compound **1** (500 MHz, CD_3OD)..18
Figure S3. ^{13}C NMR spectrum of compound **1** (125 MHz, CD_3OD). 18
Figure S4. ^{1}H-^{1}H COSY spectrum of compound **1** (500 MHz, CD_3OD)...19
Figure S5. ^{1}H-^{13}C HMBC spectrum of compound **1** (500 MHz, CD_3OD). 19
Figure S6. ^{1}H-^{13}C HSQC spectrum of compound **1** (500 MHz, CD_3OD). 20
Figure S7. ^{1}H-^{1}H ROESY spectrum of compound **1** (500 MHz, CD_3OD). 20
Figure S8. 1H NMR spectrum of compound **2** (500 MHz, CD_3OD)...21
Figure S9. 13C NMR spectrum of compound **2** (125 MHz, CD_3OD). 21
Figure S10. ^{1}H-^{1}H COSY spectrum of compound **2** (500 MHz, CD_3OD)...22
Figure S11. ^{1}H-^{13}C HMBC spectrum of compound **2** (500 MHz, CD_3OD). 22
Figure S12. ^{1}H-^{13}C HSQC spectrum of compound **2** (500 MHz, CD_3OD). 23
Figure S13. ^{1}H-^{1}H ROESY spectrum of compound **2** (500 MHz, CD_3OD). 23
Figure S14. ^{1}H NMR spectrum of compound **3** (500 MHz, CD_3OD)..24
Figure S15. ^{13}C NMR spectrum of compound **3** (125 MHz, CD_3OD). 24
Figure S16. ^{1}H-^{1}H COSY spectrum of compound **3** (500 MHz, CD_3OD)...25
Figure S17. ^{1}H-^{13}C HMBC spectrum of compound **3** (500 MHz, CD_3OD). 25
Figure S18. ^{1}H-^{13}C HSQC spectrum of compound **3** (500 MHz, CD_3OD). 26
Figure S19. ^{1}H NMR spectrum of compound **4** (500 MHz, CD_3OD)..26
Figure S20. ^{13}C NMR spectrum of compound **4** (125 MHz, CD_3OD). 27
Figure S21. ^{1}H-^{1}H COSY spectrum of compound **4** (500 MHz, CD_3OD)...27
Figure S22. ^{1}H-^{13}C HMBC spectrum of compound **4** (500 MHz, CD_3OD). 28
Figure S23. ^{1}H-^{13}C HSQC spectrum of compound **4** (500 MHz, CD_3OD). 28
Figure S24. ^{1}H-^{1}H ROESY spectrum of compound **4** (500 MHz, CD_3OD). 29
Figure S25. ^{1}H NMR spectrum of compound **5** (500 MHz, CD_3OD)..29
Figure S26. ^{13}C NMR spectrum of compound **5** (125 MHz, CD_3OD). 30
Figure S27. ^{1}H-^{1}H COSY spectrum of compound **5** (500 MHz, CD_3OD)...30
Figure S28. ^{1}H-^{13}C HMBC spectrum of compound **5** (500 MHz, CD_3OD). 31
Figure S29. ^{1}H-^{13}C HSQC spectrum of compound **5** (500 MHz, CD_3OD). 31

Figure S30. ^1H-^1H ROESY spectrum of compound **5** (500 MHz, CD_3OD). 32
Figure S31. ^1H NMR spectrum of compound **6** (500 MHz, CD_3OD)....32
Figure S32. ^{13}C NMR spectrum of compound **6** (125 MHz, CD_3OD). 33
Figure S33. ^1H-^1H COSY spectrum of compound **6** (500 MHz, CD_3OD)....33
Figure S34. ^1H-^{13}C HMBC spectrum of compound **6** (500 MHz, CD_3OD). 34
Figure S35. ^1H-^{13}C HSQC spectrum of compound **6** (500 MHz, CD_3OD). 34
Figure S36. ^1H-^1H ROESY spectrum of compound **6** (500 MHz, CD_3OD). 35
Figure S37. ^1H NMR spectrum of compound **7** (500 MHz, CD_3OD)....35
Figure S38. ^{13}C NMR spectrum of compound **7** (125 MHz, CD_3OD). 36
Figure S39. ^1H-^1H COSY spectrum of compound **7** (500 MHz, CD_3OD)....36
Figure S40. ^1H-^{13}C HMBC spectrum of compound **7** (500 MHz, CD_3OD). 37
Figure S41. ^1H-^{13}C HSQC spectrum of compound **7** (500 MHz, CD_3OD). 37
Figure S42. ^1H-^1H ROESY spectrum of compound **7** (500 MHz, CD_3OD). 38
Figure S43. ^1H NMR spectrum of compound **8** (500 MHz, CD_3OD)....38
Figure S44. ^{13}C NMR spectrum of compound **8** (125 MHz, CD_3OD). 39
Figure S45. ^1H-^1H COSY spectrum of compound **8** (500 MHz, CD_3OD)....39
Figure S46. ^1H-^{13}C HMBC spectrum of compound **8** (500 MHz, CD_3OD). 40
Figure S47. ^1H-^{13}C HSQC spectrum of compound **8** (500 MHz, CD_3OD). 40
Figure S48. ^1H-^1H ROESY spectrum of compound **8** (500 MHz, CD_3OD). 41
Figure S49. ^1H NMR spectrum of compound **9** (500 MHz, CD_3OD)....41
Figure S50. ^{13}C NMR spectrum of compound **9** (125 MHz, CD_3OD). 42
Figure S51. ^1H-^1H COSY spectrum of compound **9** (500 MHz, CD_3OD)....42
Figure S52. ^1H-^{13}C HMBC spectrum of compound **9** (500 MHz, CD_3OD). 43
Figure S53. ^1H-^{13}C HSQC spectrum of compound **9** (500 MHz, CD_3OD). 43
Figure S54. ^1H NMR spectrum of compound **10** (500 MHz, CD_3OD)....44
Figure S55. ^{13}C NMR spectrum of compound **10** (125 MHz, CD_3OD)....44
Figure S56. ^1H-^1H COSY spectrum of compound **10** (500 MHz, CD_3OD)....45
Figure S57. ^1H-^{13}C HMBC spectrum of compound **10** (500 MHz, CD_3OD). 45
Figure S58. ^1H-^{13}C HSQC spectrum of compound **10** (500 MHz, CD_3OD). 46
Figure S59. ^1H-^1H ROESY spectrum of compound **10** (500 MHz, CD_3OD). 46
Figure S60. ^1H NMR spectrum of compound **11** (500 MHz, CD_3OD)....47
Figure S61. ^{13}C NMR spectrum of compound **11** (125 MHz, CD_3OD)....47
Figure S62. ^1H-^1H COSY spectrum of compound **11** (500 MHz, CD_3OD)....48
Figure S63. ^1H-^{13}C HMBC spectrum of compound **11** (500 MHz, CD_3OD). 48
Figure S64. ^1H-^{13}C HSQC spectrum of compound **11** (500 MHz, CD_3OD). 49
Figure S65. ^1H NMR spectrum of compound **12** (500 MHz, CD_3OD)....49
Figure S66. ^{13}C NMR spectrum of compound **12** (125 MHz, CD_3OD)....50
Figure S67. ^1H-^1H COSY spectrum of compound **12** (500 MHz, CD_3OD)....50
Figure S68. ^1H-^{13}C HMBC spectrum of compound **12** (500 MHz, CD_3OD). 51
Figure S69. ^1H-^{13}C HSQC spectrum of compound **12** (500 MHz, CD_3OD). 51
Figure S70. ^1H-^1H ROESY spectrum of compound **12** (500 MHz, CD_3OD). 52
Figure S71. IR spectrum of compound **3**. 52
Figure S72. IR spectrum of compound **12**. 53
Figure S73. GC-MS Sugar analysis after hydrolysis of compound **1**. 53
Figure S74. GC-MS Sugar analysis after hydrolysis of compound **2**. 54
Figure S75. GC-MS Sugar analysis after hydrolysis of compound **3**. 54
Figure S76. GC-MS Sugar analysis after hydrolysis of compound **9**. 55
Figure S77. GC-MS Sugar analysis after hydrolysis of compound **10**. 55

Table S1: LC-MS^1 data in positive and negative modes.

#	t_R (min)	*m*/*z*	(Adduct) Ion	Mass deviation (ppm)	Formula M	Name
**1**	11.28	716.3846	$[M+NH_4]^+$	−0.8	$C_{35}H_{54}O_{14}$	5β-sarmentogenin 3-O-[β-D-glucopyranosyl-(1→4)-α-L-rhamnopyranoside] (**3**)
		699.3580	$[M+H]^+$	−0.9		
		743.3469	$[M+FA−H]^−$	−2.1		
		760.3363	$[M+NO_3]^−$	−3.0		
		733.3187	$[M+Cl]^−$	−1.4		
		697.3424	$[M−H]^−$	−0.8		
**2**	12.59	876.4585	$[M+NH_4]^+$	−0.2	$C_{42}H_{66}O_{18}$	5β-sarmentogenin 3-*O*-[β-D-glucopyranosyl-(1→6)-β-D-glucopyranosyl-(1→4)-α-L-oleandroside] (**2**)
		859.4320	$[M+H]^+$	−0.3		
		903.4200	$[M+FA−H]^−$	−2.3		
		920.4089	$[M+NO_3]^−$	−3.6		
		893.3923	$[M+Cl]^−$	−1.0		
		857.4154	$[M−H]^−$	−1.4		
**3**	13.70	714.4050	$[M+NH_4]^+$	−1.3	$C_{36}H_{56}O_{13}$	5β-sarmentogenin-3-O-[β-D-glucopyranosyl-(1→4)-α-L-oleandroside] (**1**)
		697.3758	$[M+H]^+$	−5.1		
		741.3677	$[M+FA−H]^−$	−2.0		
		758.3571	$[M+NO_3]^−$	−2.9		
		731.3392	$[M+Cl]^−$	−1.6		
		695.3619	$[M−H]^−$	−2.6		

Table S2: LC-MS$^{2/3}$ data in negative ion mode.

#	Formula M	ISF energy (V)	MS^2 parent ion	*m/z* MS^2 parent ion	CID energy MS^2 (%)	*m/z* MS^2 fragments (−neutral loss derived from MS^2 parent ion)	*m/z* MS^3 parent Ion	CID energy MS^3 (%)	*m/z* MS^3 fragments (−neutral loss derived from MS^3 parent ion)
**1**	$C_{35}H_{54}O_{14}$	40	$[M-H]^-$	697.3	40	535.2896 ($-C_6H_{10}O_5$), 517.2792 ($-C_6H_{10}O_5$, $-H_2O$), 491.2998 ($-CO_2$), 473.2893 ($-CO_2$, $-H_2O$), 455.2787 ($-CO_2$, $-2 \times H_2O$), 429.2632 ($-C_2H_2$, $-CO_2$, $-2 \times H_2O$), 389.2320 ($-C_6H_{10}O_5$, $-C_6H_{10}O_4$), 371.2215 ($-C_6H_{10}O_5$, $-C_6H_{10}O_4$, $-H_2O$), 345.2423 ($-C_6H_{10}O_5$, $-C_6H_{10}O_4$, $-CO_2$), 327.2318 ($-C_6H_{10}O_5$, $-C_6H_{10}O_4$, $-CO_2$, $-H_2O$), 309.2213 ($-C_6H_{10}O_5$, $-C_6H_{10}O_4$, $-CO_2$, $-2 \times H_2O$), 283.2058 ($-C_6H_{10}O_5$, $-C_6H_{10}O_4$, $-C_2H_2$, $-CO_2$, $-2 \times H_2O$).	535.3	40	517.2789 ($-H_2O$), 491.2997 ($-CO_2$), 473.2896 ($-CO_2$, $-H_2O$), 455.2788 ($-CO_2$, $-2 \times H_2O$), 429.2630 ($-C_2H_2$, $-CO_2$, $-2 \times H_2O$), 389.2319 ($-C_6H_{10}O_4$), 371.2215 ($-C_6H_{10}O_4$, $-H_2O$), 345.2423 ($-C_6H_{10}O_4$, $-CO_2$), 327.2318 ($-C_6H_{10}O_4$, $-CO_2$, $-H_2O$), 309.2213 ($-C_6H_{10}O_4$, $-CO_2$, $-2 \times H_2O$), 283.2059 ($-C_6H_{10}O4$, $-C_2H_2$, $-CO_2$, $-2 \times H_2O$).
**2**	$C_{42}H_{66}O_{18}$	60	$[M-H]^-$	857.4	40	695.3616 ($-C_6H_{10}O_5$), 533.3094 ($-2 \times C_6H_{10}O_5$), 427.2836 ($-2 \times C_6H_{10}O_5$, $-C_2H_2$, $-CO_2$, $-2 \times H_2O$), 371.2206 ($-2 \times C_6H_{10}O_5$, $-C_7H_{12}O_3$, $-H_2O$),	533.3	40	471.3083 ($-CO_2$, $-H_2O$), 427.2830 ($-C_2H_2$, $-CO_2$, $-2 \times H_2O$), 371.2207 ($-C_7H_{12}O_3$, $-H_2O$), 327.2318 ($-C_7H_{12}O_3$, $-CO_2$, $-H_2O$), 309.2210 ($-C_7H_{12}O_3$, $-CO_2$, $-2 \times H_2O$).
**3**	$C_{36}H_{56}O_{13}$	0	$[M+FA-H]^-$	741.3	40	695.3630 (−FA), 533.3104 (−FA, $-C_6H_{10}O_5$).	533.3	40	471.3096 ($-CO_2$, $-H_2O$), 427.2842 ($-C_2H_2$, $-CO_2$, $-2 \times H_2O$), 371.2212 ($-C_7H_{12}O_3$, $-H_2O$).

Table S3: ^{13}C (125 MHz) and ^{1}H (500 MHz) NMR data of compound **1** (CD_3OD). [a]

Position	δ_C	type	δ_H	(J Hz)
1	34.4	CH_2	1.41	
			2.36	m
2	28.5	CH_2	1.54	
			1.81	
3α	74.0	CH	3.89	br s
4	31.8	CH_2	1.84	
			1.44	
5β	39.9	CH	1.63	
6	28.4	CH_2	1.26	
			1.86	
7	22.8	CH_2	1.28	
			1.81	
8	42.0	CH	1.65	
9	43.1	CH	1.79	
10	37.7	C		
11β	69.0	CH	3.74	
12	50.7	CH_2	1.57	
			1.68	
13	51.2	C		
14	85.8	C		
15	33.8	CH_2	2.21	
			1.76	
16	28.1	CH_2	1.89	dt (9.2, 4.6, 4.6)
			2.19	
17α	52.0	CH	2.91	m
18	17.7	CH_3	0.90	s
19	24.7	CH_3	1.08	s
20	177.8	C		
21	75.5	CH_2	4.93	dd (18.6, 1.8)
			5.01	dd (18.3, 1.2)
22	118.1	CH	5.92	s
23	177.3	C		
1'	97.1	CH	4.97	d (2.8)
2'	36.5	CH_2	2.25	
			1.49	
3'	80.1	CH	3.74	
4'	83.4	CH	3.35	
5'	68.4	CH	3.75	
6'	18.6	CH_3	1.27	
7'	57.1	CH_3	3.41	s
1"	105.2	CH	4.62	d (7.9)
2"	76.0	CH	3.17	dd (9.2, 7.9)
3"	78.1	CH	3.36	
4"	72.0	CH	3.28	
5"	78.1	CH	3.23	m
6"	63.1	CH_2	3.86	dd (11.8, 2.3)
			3.67	dd (11.9, 5.5)

[a] Overlapped signals are reported without multiplicity.

Table S4: ^{13}C (125 MHz), ^{1}H, ^{1}H-^{1}H COSY, ^{1}H-^{13}C HMBC, and ^{1}H-^{1}H ROESY (500 MHz) NMR data of compound **2** (CD_3OD).[a]

Position	δ_C	type	δ_H	(*J* Hz)	COSY	HMBC	ROESY
1	34.3	CH_2	1.40		H2a, H2b		
			2.35		H2a, H2b		H19
2	28.5	CH_2	1.53		H1a, H1b		H3, H1’a
			1.82	m	H1a, H1b		H3
3	74.0	CH	3.89	br s	H4a		H2a, H2b, H4a, H4b, H1’
4	31.8	CH_2	1.43	m	H3a, H5	C-6	H3, H19, H1’a
			1.84	m	H5	C-5	H3
5	39.9	CH	1.63		H3a, H3b, H6b		H19
6	28.4	CH_2	1.91	br dd (9.2, 5.2)			
			1.27	m	H5, H7b		
7	22.8	CH_2	1.28	m	H8		H9
			1.79	m	H5	C-8, C-10, C-11	
8	42.0	CH	1.66		H7a, H9	C-7, C-11, C-13, C-14	H11, H18, H19
9	43.1	CH	1.79		H8, H11	C-11, C-19	H7a
10	37.7	C					
11	69.0	CH	3.73	m	H9, H12a, H12b	C-10	H8, H18, H19
12	50.7	CH_2	1.56		H9, H18	C-9, C-11, C-13, C-17, C-18	H17
			1.68		H9	C-9, C-11, C-13, C-14	H17
13	51.2	C					
14	85.7	C					
15	33.8	CH_2	2.22		H16a	C-16	
			1.76		H16a, H16b	C-13, C-14, C-17	H16b
16	28.1	CH_2	1.90		H15a, H17	C-17	H22
			2.19		H15a, H15b, H17	C-14	H15b, H17
17	52.0	CH	2.91	m		C-12, C-14, C-20, C-21, C-22	H12a, H12b, H16b, H18, H21
18	17.7	CH_3	0.90	s			H8, H11, H17, H21b, H22
19	24.7	CH_3	1.07	s			H1b, H4b, H5, H8, H11
20	177.8	C					
21	75.5	CH_2	4.93	dd (18.6, 1.8)		C-20, C-22, C-23	
			5.01	dd (18.6, 1.5)		C-20, C-22, C-23	H17, H18
22	118.1	CH	5.92	s		C-17, C-20, C-21, C- 23	H16a, H17, H18
23	177.3	C					
1'	97.1	CH	4.97	d (2.8)	H2’a, H2’b	C-3, C-3’, C-5’	H2a, H3a, H4b, H2’a, H6’
2'	36.4	CH_2	2.22		H1’, H3’	C-1’, C-3’, C-4’	H1’a, H3’, H7’
			1.51		H1’, H3’	C-3’	
3'	80.2	CH	3.74		H2’a, H2’b, H4’	C-6’, C-7’	H2’a, H5’, H6’’’b
4'	83.0	CH	3.40	s	H3’, H5’	C-3’, C-5’, C-6’, C-1’’	H6’
5'	68.4	CH	3.75		H4’, H6’	C-3’, C-4’	H3’a, H6’
6'	18.7	CH_3	1.26	d (6.4)		C-4’, C-5’	H1’, H4’, H5’, H1’’
7'	57.1	CH_3	3.41	m			H2’a, H1’’
1"	105.0	CH	4.64	d (7.6)	H2’’	C-4’, C-5’’	
2"	75.9	CH	3.17		H1’’,H3’’	C-1’’, C-3’’, C-4’’	
3"	78.1	CH	3.36		H2’’	C-2’’	
4"	72.0	CH	3.31		H5’’	C-3’’, C-5’’, C-6’’	
5"	77.2	CH	3.43		H4’’, H6’’a, H6’’b	C-4’’, C-6’’	H6’’b
6"	70.6	CH_2	3.78		H5’’	C-5’’, C-1’’’	H1’’’
			4.14	dd (11.6, 1.8)	H5’’	C-4’’, C-1’’’	H5’’
1"'	105.2	CH	4.40	d (7.9)	H2’’’	C-6’’, C-2’’’, C-5’’’	H6’, H6’’, H5’’’
2"'	75.3	CH	3.21		H1’’’, H3’’’	C-1’’’, C-5’’’	
3"'	78.0	CH	3.36		H2’’’, H4’’’	C-4’’’	
4"'	71.7	CH	3.28		H3’’’	C-6’’’	
5"'	78.1	CH	3.26		H6’’’a, H6’’’b		H1’’’
6"'	62.9	CH_2	3.66	dd (11.9, 5.5)	H5’’’	C-4’’’, C-6’’’	
			3.86	dd (11.75, 2.0)	H5’’’	C-4’’’	

[a] Overlapped signals are reported without multiplicity.

Table S5: ^{13}C (125 MHz), ^{1}H, ^{1}H-^{1}H COSY, ^{1}H-^{13}C HMBC, and ^{1}H-^{1}H ROESY (500 MHz) NMR data of compound **3** (CD_3OD).[a]

Position	δ_C	type	δ_H	(J Hz)	COSY	HMBC	ROESY
1	34.3	CH_2	2.36	br d (13.4)	H2b	C-9	H19, H2a
			1.39	m			
2	28.5	CH_2	1.57	m		C-9	H1a, H3, H1’
			1.83	m	H1b, H3		H3
3	74.5	CH	3.94	m	H2b, H4b	C-2’	H2a, H2b, H4a, H4b, H1’
4	31.9	CH_2	1.45	m			H3, H1’
			1.86	m		C-5	H3, H6b
5	40.0	CH	1.62	br d (13.7)			H19
6	28.1	CH_2	1.90	br d (9.5)		C-4	
			2.18	m			H4a
7	22.8	CH_2	1.81	m	H8		H16a
			1.28	s		C-8	
8	42.2	CH	1.65	br d (3.1)	H7a, H9		H17, H18, H19, H6’’b
9	43.3	CH	1.79	m	H8, H11	C-8, C-10, C-11, C-19	H16a
10	37.8	C					
11	69.0	CH	3.75	m	H9, H12a, H12b	C-10	
12	50.7	CH_2	1.68	m	H11	C-9, C-11, C-18	H17
			1.56	m	H11	C-10	H17
13	51.2	C					
14	85.7	C					
15	33.9	CH_2	1.75	m		C-13, C-17	
			2.21	m	H17	C-15, C-16	H17
16	28.4	CH_2	1.25	br d (2.8)			H7a, H9
			1.89	m		C-17	H21a, H21b
17	52.1	CH	2.90	m	H15b		H8, H12a, H12b, H18, H21a, H21b, H22
18	17.7	CH_3	0.90	s		C-12, C-14, C-17	H8, H17, H21a, H21b, H6’’b
19	24.7	CH_3	1.07	s		C-1, C-2, C-5, C-9, C-10	H1a, H5, H8, H6’’b
20	177.7	C					
21	75.5	CH_2	4.91	dd (18.6, 1.8)		C-20, C-22	H16b, H17, H18, H22
			5.01	dd (18.6, 1.2)		C-22	H16b, H17, H18
22	118.1	CH	5.90	s		C-17, C-21, C-23	H17, H21a
23	177.2	C					
1'	100.1	CH	4.77	d (1.2)	H2’	C-3, C-3’, C-5’	H2a, H3, H4a, H2’, H3’, H6’
2'	73.1	CH	3.79	m	H1’, H3’	C-3’	H1’
3'	72.8	CH	3.93	m	H2’, H4’	C-4’	H1’, H4’, H5’, H6’’b
4'	84.0	CH	3.61	m	H3’, H5’	C-3’, C-5’, C-6’, C-1’’	H6’, H1’’
5'	68.8	CH	3.75	m	H4’, H6’	C-4’	H3’
6'	18.2	CH_3	1.31	d (6.4)		C-4’, C-5’	H1’, H4’, H1’’, H6’’b
1"	106.0	CH	4.58	m	H2’’	C-3’’	H4’, H6’
2"	76.3	CH	3.23	m	H1’’	C-1’’, C-3’’	
3"	78.5	CH	3.38	m		C-2’’, C-4’’	
4"	71.9	CH	3.33	m		C-5’’, C-6’’	
5"	78.2	CH	3.28	m	H6’’a, H6’’b		
6"	63.1	CH_2	3.85	m	H5’’	C-4’’	
			3.70	m	H5’’	C-4’’, C-5’’	H8, H18, H19, H3’, H6’

[a] Overlapped signals are reported without multiplicity.

Table S6: ^{13}C (125 MHz), ^{1}H, ^{1}H-^{1}H COSY, ^{1}H-^{13}C HMBC, and ^{1}H-^{1}H ROESY (500 MHz) NMR data of compound **4** (CD_3OD).[a]

Position	δ_C	type	δ_H	(J Hz)	COSY	HMBC	ROESY
1	44.4	CH_2	1.65	t (12.8, 12.8)	H2	C-2, C-3, C-9, C-10, C-19	H2
			2.31	dd (12.5, 5.5)	H2	C-2, C-3, C-5, C-6, C-10, C-19	H2, H11a, H19
2	70.9	CH	4.44	dd (13.1, 5.5)	H1a, H1b	C-1, C-3	H1a, H1b, H19
3	201.6	C					
4	122.8	CH	5.71	s		C-2, C-6, C-10	H6
5	166.3	C					
6	129.0	CH	6.29	dd (10.1, 2.8)		C-8, C-10	H4, H8
7	141.1	CH	6.31	dd (10.1, 1.5)	H8	C-5, C-9	H8, H15a
8	38.1	CH	2.49		H9, H14	C-9, C-13, C-15	H6, H7, H11a, H12a, H18
9	52.6	CH	1.34		H8		
10	39.5	C					
11	21.2	CH_2	1.55		H12b		H1b, H8, H18
			1.71		H12a, H12b		
12	32.6	CH_2	1.32	br d (4.6)	H11b	C-11, C-13, C-17, C-18	H8
			1.85	m	H11a, H11b	C-13	H18
13	49.8	C					
14	50.0	CH	1.55		H8, H15a	C-8, C-9, C-12, C-13, C-15, C-18	H15a, H15b
15	22.4	CH_2	2.18		H14, H16a	C-13, C-16	H7, H14, H16a
			1.79		H16a, H16b		H14, H16a ,H16b
16	36.6	CH_2	2.52		H15a, H15b		H15b, H15b
			2.14		H15b	C-15	H15b
17	214.1	C					
18	14.2	CH_3	0.99	s		C-12, C-13	H8, H11a, H12b
19	17.6	CH_3	1.28	s		C-1, C-9, C-10	H1b, H2, H8

[a] Overlapped signals are reported without multiplicity.

Table S7: ^{13}C (125 MHz), ^{1}H, ^{1}H-^{1}H COSY, ^{1}H-^{13}C HMBC, and ^{1}H-^{1}H ROESY (500 MHz) NMR data of compound **5** (CD_3OD).[a]

Position	δ_C	type	δ_H	(*J* Hz)	COSY	HMBC	ROESY
1	44.1	CH_2	1.68		H2a	C-2, C-3, C-9, C-10, C-19	H2
			2.32	dd (12.4, 5.7)	H2a	C-2, C-3, C-19	H2, H11b, H19
2	70.9	CH	4.44	dd (13.4, 5.5)	H1a, H1b	C-1, C-3	H1a, H1b
3	201.5	C					
4	122.8	CH	5.73	s		C-2, C-6, C-10	H6, H7
5	165.5	C					
6	129.3	CH	6.29	dd (10.1, 2.8)	H7	C-5, C-8	H4, H7, H8, H15b
7	139.6	CH	6.35	dd (10.1, 1.5)	H6	C-5, C-8, C-9, C-13	H4, H8, H15b
8	40.6	CH	2.15	br t (10.7, 10.7)	H14		H6, H7, H18, H19
9	51.1	CH	1.48	m	H11b		
10	39.4	C					
11	22.6	CH_2	1.44		H12b	C-13	H12a
			1.83		H9, H12a, H12b		H1b, H12b, H18
12	40.3	CH_2	1.71		H11a	C-11, C-13	H11a
			2.02	dt (12.7, 3.1, 3.1)	H11a, H11b		H11b, H18
13	84.9	C					
14	44.9	CH	1.67		H8	C-9, C-13, C-15	
15	20.4	CH_2	1.78		H16a, H16b	C-16	H16a, H16b
			2.25		H16a, H16b		H6, H7, H14, H16a, H16b
16	29.2	CH_2	2.64	m	H15a, H15b	C-17	H15a, H15b
			2.79	m		C-17	H15a, H15b
17	174.3	C					
18	20.3	CH_3	1.43	s		C-12, C-13, C-14	H8, H11b, H12b
19	17.5	CH_3	1.24	s		C-1, C-5, C-9, C-10	H1b, H2, H8

[a] Overlapped signals are reported without multiplicity.

Table S8: ^{1}H (500 MHz) NMR data of compounds **6-8** (CD_3OD). [a]

	**6**		**7**		**8**	
Position	δ_H	(J Hz)	δ_H	(J Hz)	δ_H	(J Hz)
1	1.75		2.29		2.08	
	1.97	m	1.64		1.74	br d (4.9)
2	2.40	m	4.43	dd (13.1, 5.5)	2.39	m
	2.65				2.64	
4	5.72	s	5.69	s	5.67	s
6	6.27	s	6.22		6.2	dd (10.1, 2.8)
7	6.27	s	6.22		6.24	dd (9.8, 1.2)
8	2.77		2.31		2.29	
9	1.66	ddd (13.9, 10.2, 4.6)	1.33	m	1.29	m
11	2.27	dd (13.9, 4.4)	1.52		1.50	
	2.74		1.65		1.68	
12			1.99		1.48	
			1.47		1.99	
14	1.74		1.41		1.42	
15	1.74		1.95		1.49	
	2.06	m	1.49		1.96	
16	1.85	m	1.79		1.79	
	2.23	m	2.24		2.26	
17α	3.23	t (9.3, 9.3)	2.65	m	2.65	
18	1.09	s	0.74	s	0.75	s
19	1.25	s	1.24	s	1.16	s
21	4.25	d (19.2)	4.16	d (18.6)	4.17	d (18.6)
	4.64	d (19.2)	4.21	d (18.9)	4.22	d (18.6)

[a] Overlapped signals are reported without multiplicity.

Table S9: ^{13}C (125 MHz) NMR data of compounds **6-8** (CD_3OD). [a]

	**6**		**7**		**8**	
Position	δ_C	type	δ_C	type	δ_C	type
1	34.7	CH_2	44.0	CH_2	35.1	CH_2
2	34.7	CH_2	70.5	CH	34.9	CH_2
3	202.0	C	201.3	C	202.5	C
4	124.9	CH	122.1	CH	124.1	CH
5	165.5	C	166.3	C	167.2	C
6	129.8	CH	128.1	CH	129.1	CH
7	141.0	CH	142.5	CH	143.0	CH
8	38.1	CH	38.3	CH	39.2	CH
9	53.1	CH	52.0	CH	52.2	CH
10	37.9	C	39.0	C	37.6	C
11	38.4	CH_2	21.4	CH_2	21.8	CH_2
12	213.7	C	39.1	CH_2	39.5	CH_2
13	60.4	C	46.1	C	46.5	C
14	55.5	CH	54.5	CH	55.1	CH
15	24.5	CH_2	24.7	CH_2	25.1	CH_2
16	24.3	CH_2	23.7	CH_2	24.1	CH_2
17	51.0	CH	59.2	CH	59.6	CH
18	13.9	CH_3	13.5	CH_3	13.9	CH_3
19	16.2	CH_3	17.2	CH_3	16.7	CH_3
20	212.2	C	211.6	C	211.9	C
21	70.1	CH_2	69.9	CH_2	70.3	CH_2

[a] Overlapped signals are reported without multiplicity.

Table S10: ^{13}C (125 MHz), ^{1}H, ^{1}H-^{1}H COSY, and ^{1}H-^{13}C HMBC (500 MHz) NMR data of compound **9** (CD_3OD).[a]

Position	δ_C	type	δ_H	(J Hz)	COSY	HMBC
1	71.4	CH_2	3.59	m	H2a, H2b	
			3.96	td (9.2, 9.2, 5.5)	H2a, H2b	C-2, C-3, C-1’
2	31.8	CH_2	1.46	m	H1a, H1b, H3	C-1, C-7
			1.93	dddd (9.1, 6.8, 4.5, 2.1)	H1a, H1b, H3	
3	49.5	CH	1.26	m	H2a, H2b, H7a	
4	74.6	C				
5	26.4	CH_3	1.13	m		C-3, C-4, C-6
6	28.2	CH_3	1.18	s		C-3, C-4, C-5
7	25.3	CH_2	1.15	m	H3	
			1.60	ddd (13.4, 7.6, 3.4)	H8	
8	14.2	CH_3	0.97	t (7.5, 7.5)		C-3, C-7
1'	104.7	CH	4.27	d (7.9)	H2’	C-1, C-3’, C-5’
2'	75.2	CH	3.19		H1’, H3’	
3'	78.2	CH	3.49		H2’	
4'	71.6	CH	3.35		H5’	C-2’, C-5’
5'	77.2	CH	3.44	m	H4’, H6’a, H6’b	
6'	69.9	CH_2	3.78	dd (11.6, 5.5)	H5’	C-5’
			4.15	dd (11.4, 2.0)	H5’	C-4’
1"	105.0	CH	4.37	d (7.6)	H2’’	C-6’, C-2’’, C-3’’
2"	75.3	CH	3.19	m	H1’’, H3’’	C-1’’
3"	78.1	CH	3.35		H2’’	C-2’’, C-4’’, C-5’’
4"	71.7	CH	3.28			C-3’’
5"	78.1	CH	3.27		H6’’a, H6’’b	
6"	62.9	CH_2	3.66	dd (11.9, 5.2)	H5’’	C-3’’
			3.87	dd (11.9, 1.8)	H5’’	C-4’’

[a] Overlapped signals are reported without multiplicity.

Table S11: ^{13}C (125 MHz), ^{1}H, ^{1}H-^{1}H COSY, ^{1}H-^{13}C HMBC, and ^{1}H-^{1}H ROESY (500 MHz) NMR data of compound **10** (CD_3OD).[a]

Position	δ_C	type	δ_H	(J Hz)	COSY	HMBC	ROESY
1	67.4	CH_2	4.29	m		C-2, C-3, C-1’	H2, H7a, H7b, H8
			4.43	dd (12.2, 6.1)	H2	C-2, C-3, C-1’	H2, H8
2	120.4	CH	5.71			C-4, C-7	H1b, H2b, H5, H6
3	153.1	C					
4	74.4	C					
5	29.7	CH_3	1.33	s		C-4, C-6	H2, H7a
6	29.7	CH_3	1.33	s		C-4, C-5	H2, H7a
7	21.9	CH_2	2.18	dd (7.6, 2.1)	H8	C-2, C-3, C-4, C-8	H1a, H5, H6, H8
			2.20	dd (7.6, 2.4)	H8		H1a
8	16.0	CH_3	1.05	t (7.6, 7.6)	H7a, H7b	C-3, C-7	H1a, H1b, H7a
1'	103.7	CH	4.32	s	H2’	C-1, C-2’, C-3’	H2’, H3’, H5’
2'	75.3	CH	3.21		H1’, H3’	C-1’, C-3’	H1’
3'	78.3	CH	3.36				H1’
4'	71.9	CH	3.36				
5'	77.2	CH	3.45	m	H6’a, H6’b	C-4’	H1’, H6’a, H6’b
6'	70.2	CH_2	3.81	dd (11.4, 5.7)	H5’	C-5’, C-1’’	H1’’, H5’
			4.14	dd (11.4, 2.0)	H5’	C-4’	H1’’, H5’
1"	105.1	CH	4.38	d (7.6)	H2’’	C-6’	
2"	75.4	CH	3.21	m	H1’’, H3’’	C-1’’, C-3’’	H6’a, H6’b
3"	78.3	CH	3.36		H2’’		
4"	72.0	CH	3.30			C-5’’	
5"	78.2	CH	3.28		H6’’a, H6’’b	C-4’’	H6’’b
6"	63.1	CH_2	3.67	dd (11.9, 5.2)	H5’’	C-5’’	H5’’
			3.88	dd (12.1, 2.0)	H5’’		

[a] Overlapped signals are reported without multiplicity.

Table S12: ^{13}C (125 MHz), ^{1}H, ^{1}H-^{1}H COSY, and ^{1}H-^{13}C HMBC (500 MHz) NMR data of compound **11** (CD_3OD).[a]

Position	δ_C	type	δ_H	(J Hz)	COSY	HMBC
1	81.4	C				
2	33.1	CH_2	2.28	dd (16.0, 3.2)	H3	
			2.91	dt (16.2, 3.1, 3.1)	H3	
3	73.3	CH	5.33	q (3.4, 3.4, 3.4)	H2a, H2b, H4	C-1, C-9’
4	75.5	CH	3.61	m	H3, H5	
5	68.0	CH	4.23	m	H4, H6a, H6b	
6	41.4	CH_2	1.84	dd (13.7, 11.3)	H5	
			2.53	m	H5	
7	174.9	C				
1'	127.6	C				
2'	116.6	CH	6.47	d (7.9)		C-1’, C-3’
3'	146.7	C				
4'	149.4	C				
5'	115.9	CH	6.78	d (2.1)	H6’	C-6’
6'	122.3	CH	6.57	dd (8.2, 1.8)	H5’	C-5’
7'	147.2	CH	7.46	d (16.2)	H8’	C-6’, C-8’, C-9’
8'	115.6	CH	6.11	d (15.9)	H7’	C-1’, C-9’
9'	168.9	C				
1"	127.5	C				
2"	124.4	CH	6.87	dd (8.2, 1.8)		C-4’’, C-6’’
3"	149.4	C				
4"	150.8	C				
5"	116.5	CH	6.67	d (7.9)	H6’’	
6"	111.6	CH	6.94	d (1.8)	H5’’	C-2’’, C-4’’, C-7’’
7"	147.7	CH	7.51	d (15.9)	H8’’	C-2’’, C-6’’, C-8’’, C-9’’
8"	115.6	CH	6.23	d (15.9)	H7’’	C-1’’, C-9’’
9"	168.0	C				
10"	56.4	CH_3	3.71	s		C-3’’

[a] Overlapped signals are reported without multiplicity.

Table S13: ^{13}C (125 MHz), ^{1}H, ^{1}H-^{1}H COSY, ^{1}H-^{13}C HMBC, and ^{1}H-^{1}H ROESY (500 MHz) NMR data of compound **12** (CD_3OD).[a]

Position	δ_C	type	δ_H	(J Hz)	COSY	HMBC	ROESY
1	81.3	C					
2	32.8	CH_2	2.31	dd (16.0, 3.2)	H3	C-1	H6a, H3, H4
			2.95	dt (16.0, 3.3, 3.3)	H3	C-1, C-4	H6b, H3
3	73.3	CH	5.34	m	H2a, H2b, H4	C-1, C-4, C-5, C-9’	H2a, H2b, H4
4	75.5	CH	3.62	m	H3, H5	C-5	H2a, H3, H5, H6a
5	67.9	CH	4.25	ddd (11.1, 9.7, 4.6)	H6a, H6b	C-4	H4, H6a, H6b, H7’, H7’’
6	41.7	CH_2	1.82	dd (13.4, 11.3)		C-5, C-6	H2a, H4, H5
			2.51	m		C-1	H2b, H5
7	174.7	C					
1'	127.6	C					
2'	116.5	CH	6.42	d (8.2)		C-1’, C-3’, C-4’	H6’, H10’’, H11’’
3'	146.6	C					
4'	149.4	C					
5'	116.1	CH	6.75	d (1.8)	H6’	C-6’, C-7’	H7’, H8’
6'	122.0	CH	6.52	dd (8.4, 2.0)	H5’	C-4’, C-5’, C-7’	H2’, H7’, H8’, H10’’, H11’’
7'	147.2	CH	7.44	d (15.9)	H8’	C-1’, C-2’, C-6’, C-8’, C-9’	H5, H5’, H6’, H8’
8'	115.6	CH	6.09	d (15.9)	H7’	C-1’, C-9’	H7’, H5’, H6’
9'	169.0	C					
1"	126.5	C					
2"	107.0	CH	6.72	s		C-1’’, C-3’’, C-4’’, C-6’’, C-7’’	H7’’, H8’’,
3"	149.5	C					
4"	139.8	C					
5"	149.5	C					
6"	107.0	CH	6.72	s		C-1’’, C-2’’, C-4’’, C-5’’	H7’’, H8’’
7"	148.0	CH	7.52	d (15.9)	H8’’	C-1’’, C-2’’, C-6’’, C-8’’, C-9’’	H5, H2’’, H6’’, H8’’
8"	116.0	CH	6.28	d (15.9)	H7’’	C-1’’, C-7’’, C-9’’	H2’’, H6’’, H7’’
9"	167.9	C					
10"	56.8	CH_3	3.73	s		C-5’’, C-6’’	H2’, H6’
11"	56.8	CH_3	3.73	s		C-2’’, C-3’’, C-7’’	H2’, H6’

[a] Overlapped signals are reported without multiplicity.

Table S14: Hill coefficient (h) and goodness of fit (R^2) for human A549 and PBMC cells treated with compound 1,2 and 3 to analyze half-maximal inhibitory concentrations (IC_{50}).

	**Compound 1**		**Compound 2**		**Compound 3**	
	A549	PBMC	A549	PBMC	A549	PBMC
**h**	-2.961	-1.549	-4.806	-1.449	-4.193	-1.673
**R²**	0.943	0.943	0.942	0.931	0.956	0.947

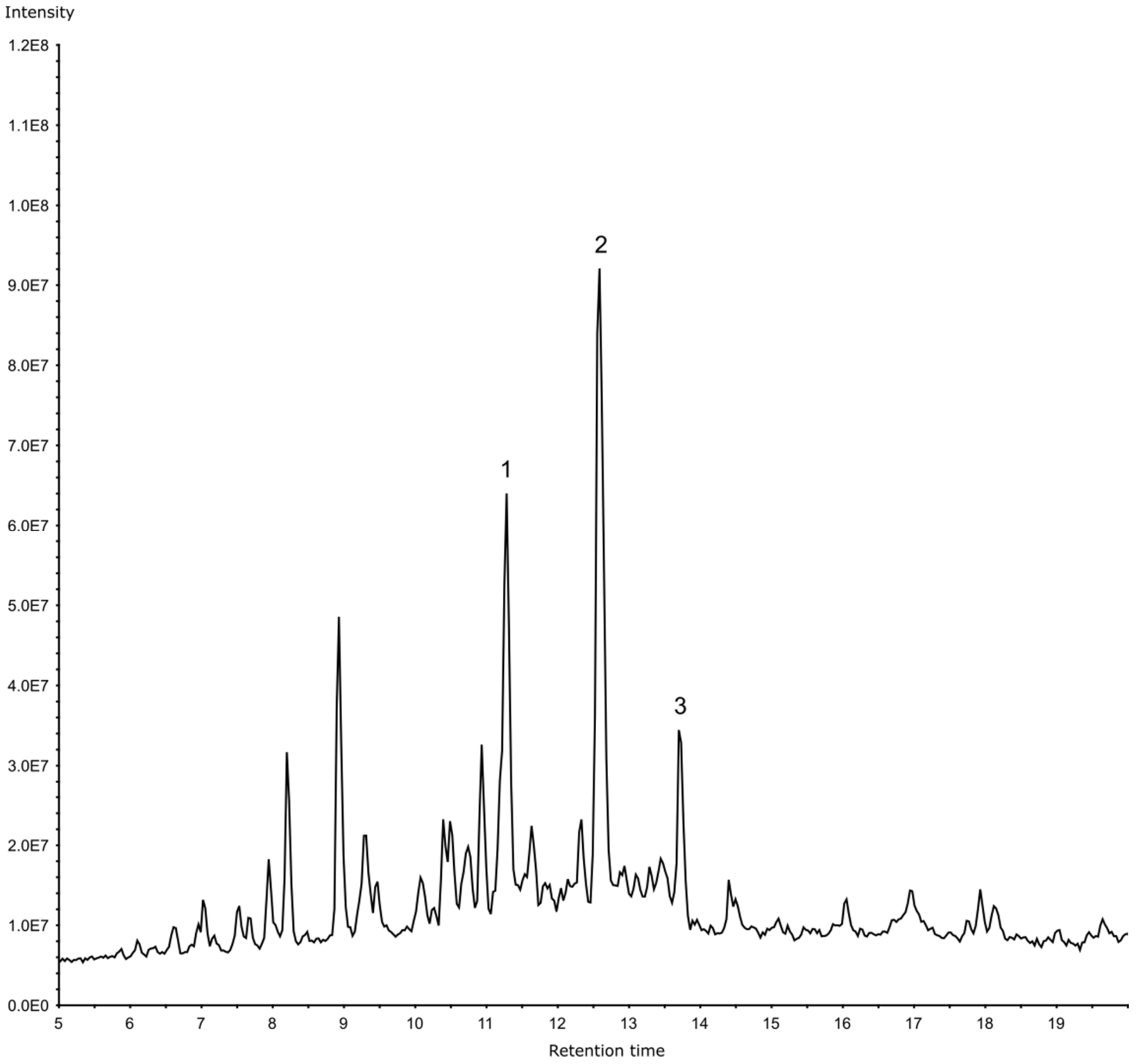

Figure S1: Excerpt of the Total Ion Chromatogram (TIC) of the MeOH extract in negative ion mode.

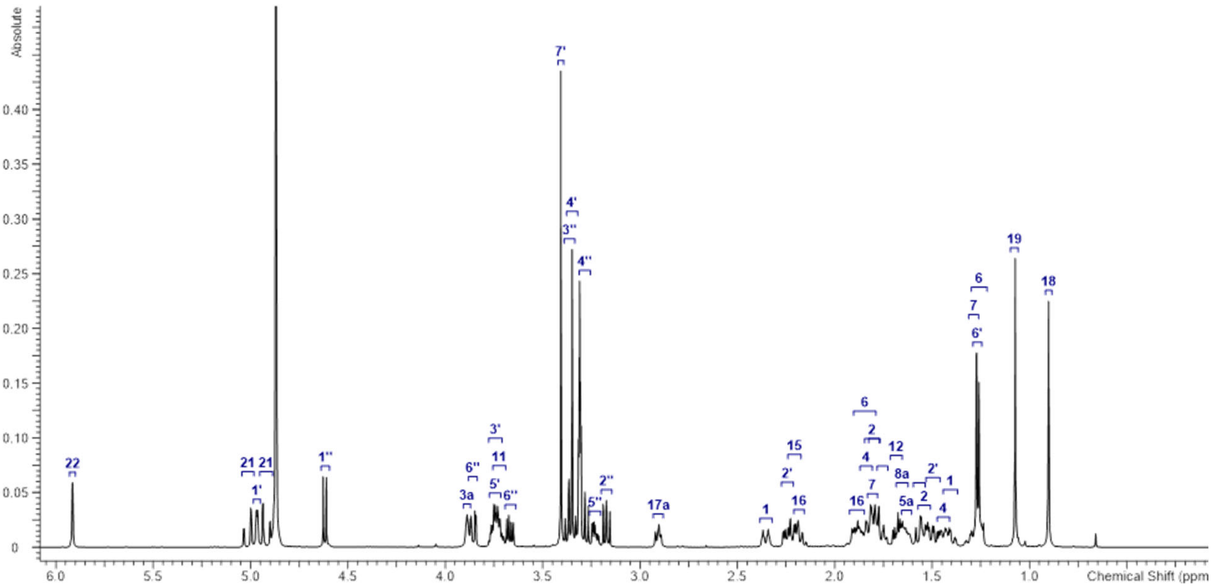

Figure S2. ^{1}H NMR spectrum of compound **1** (500 MHz, CD_3OD).

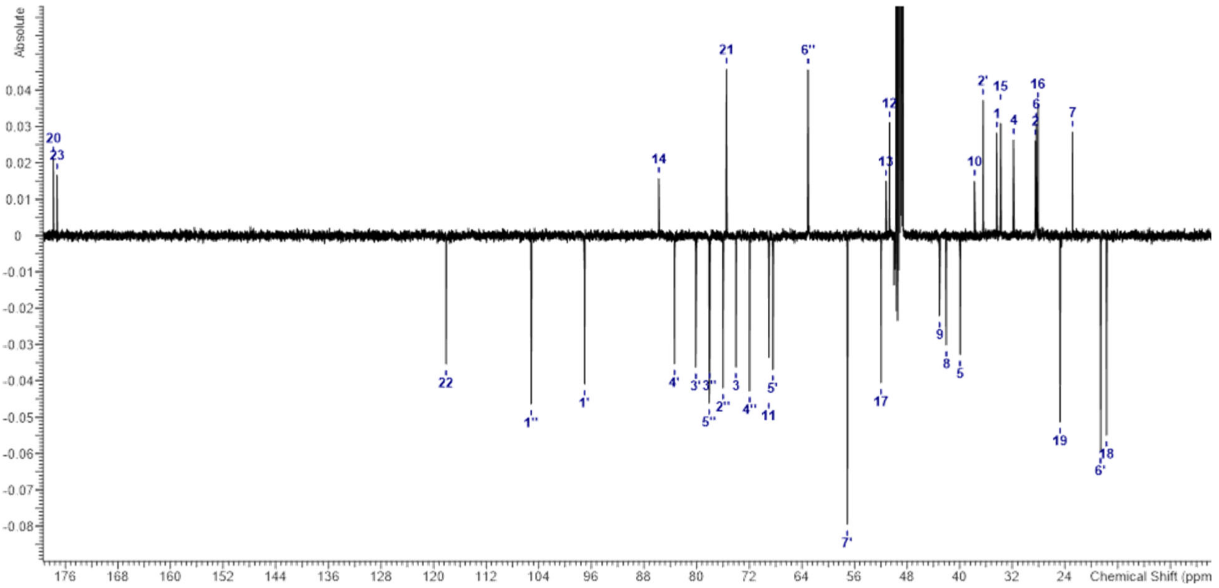

Figure S3. ^{13}C NMR spectrum of compound **1** (125 MHz, CD_3OD).

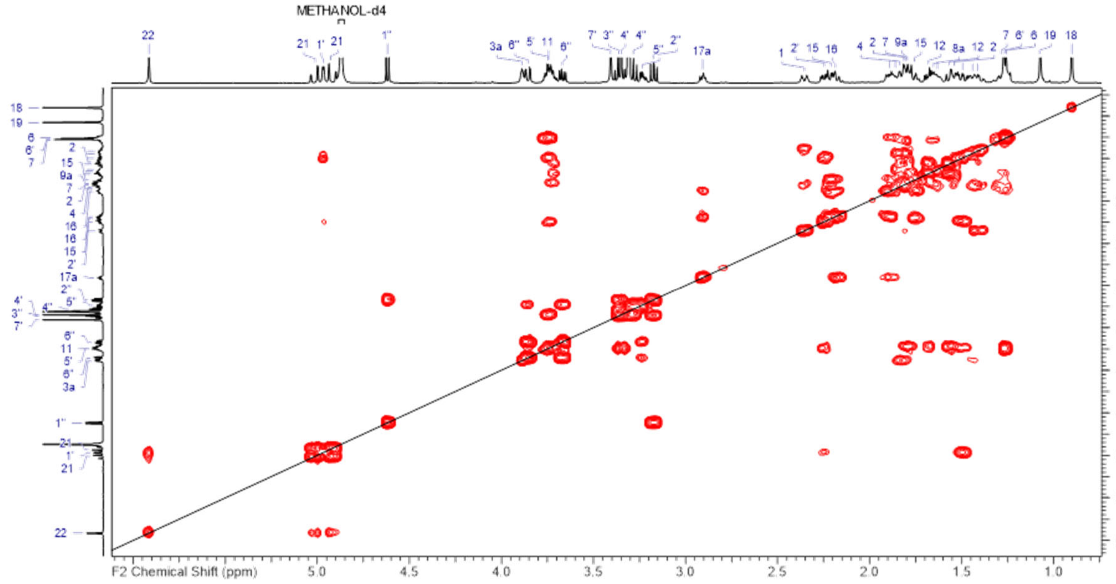

Figure S4. ^{1}H-^{1}H COSY spectrum of compound **1** (500 MHz, CD_3OD).

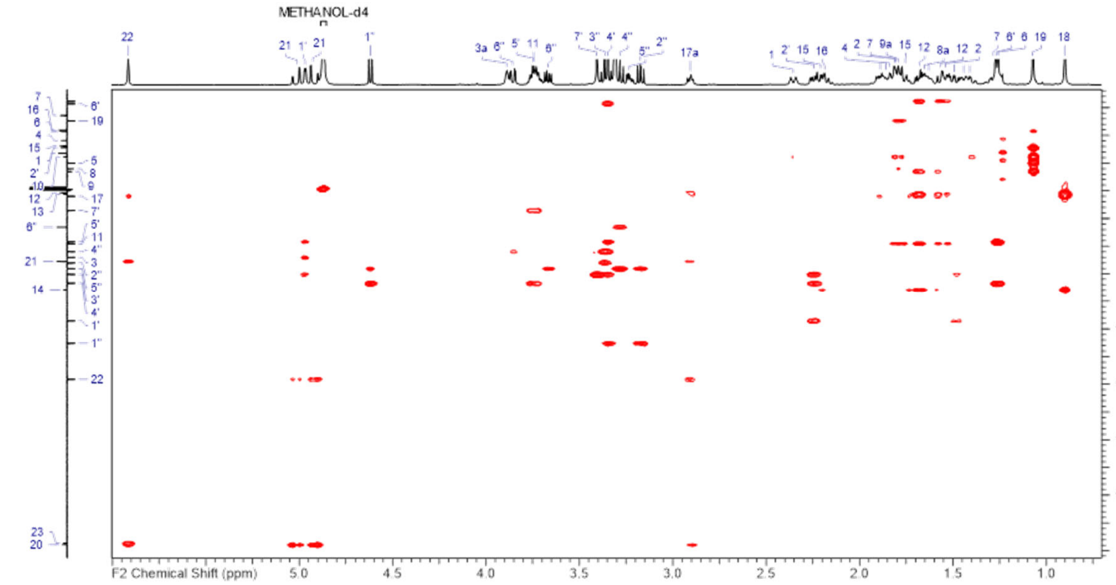

Figure S5. ^{1}H-^{13}C HMBC spectrum of compound **1** (500 MHz, CD_3OD).

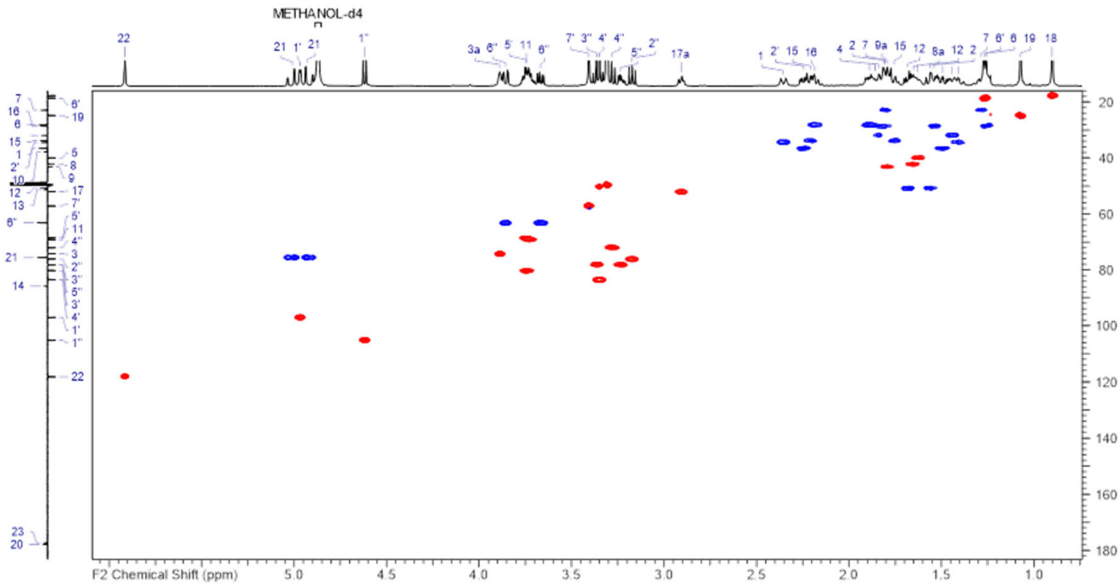

Figure S6. ^{1}H-^{13}C HSQC spectrum of compound **1** (500 MHz, CD_3OD).

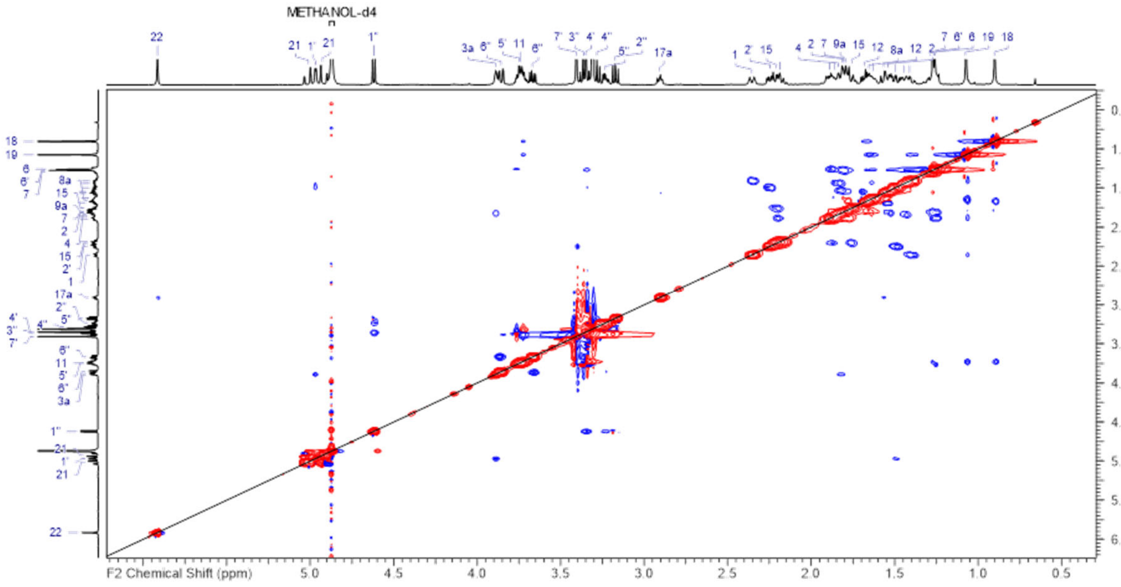

Figure S7. ^{1}H-^{1}H ROESY spectrum of compound **1** (500 MHz, CD_3OD).

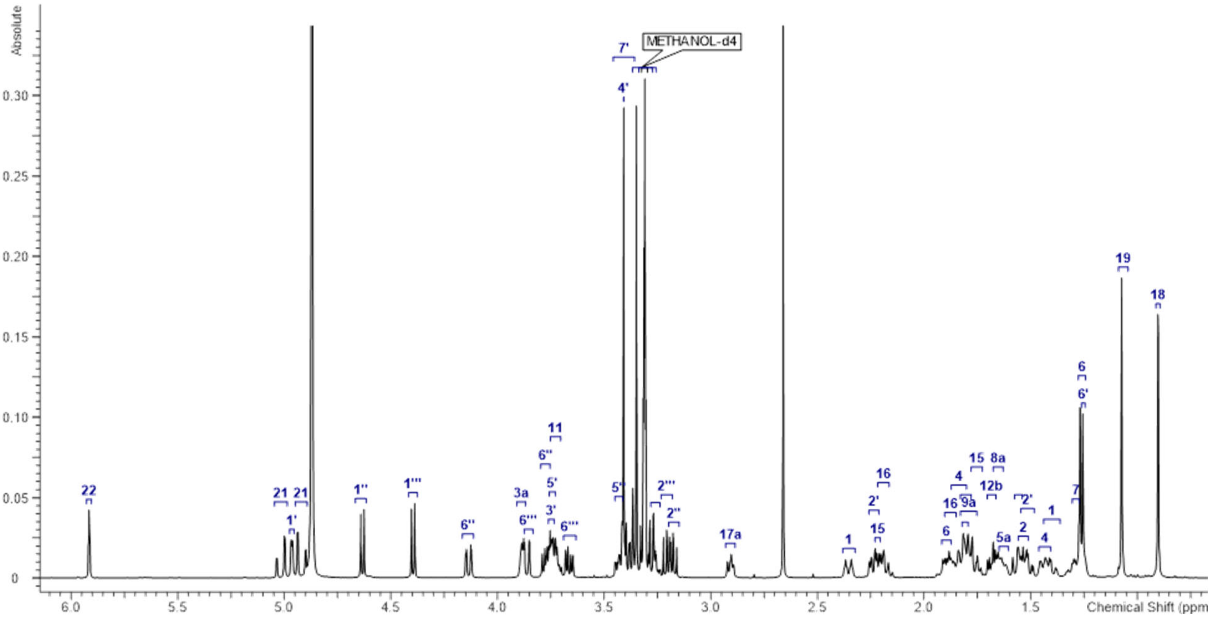

Figure S8. ^{1}H NMR spectrum of compound **2** (500 MHz, CD_3OD).

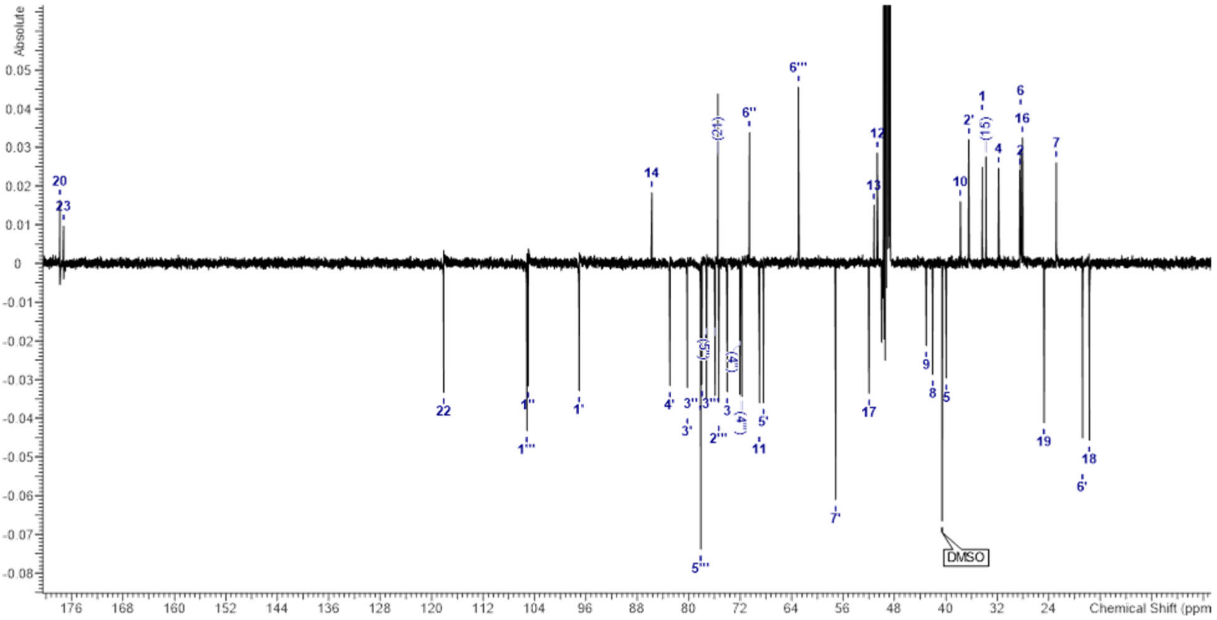

Figure S9. ^{13}C NMR spectrum of compound **2** (125 MHz, CD_3OD).

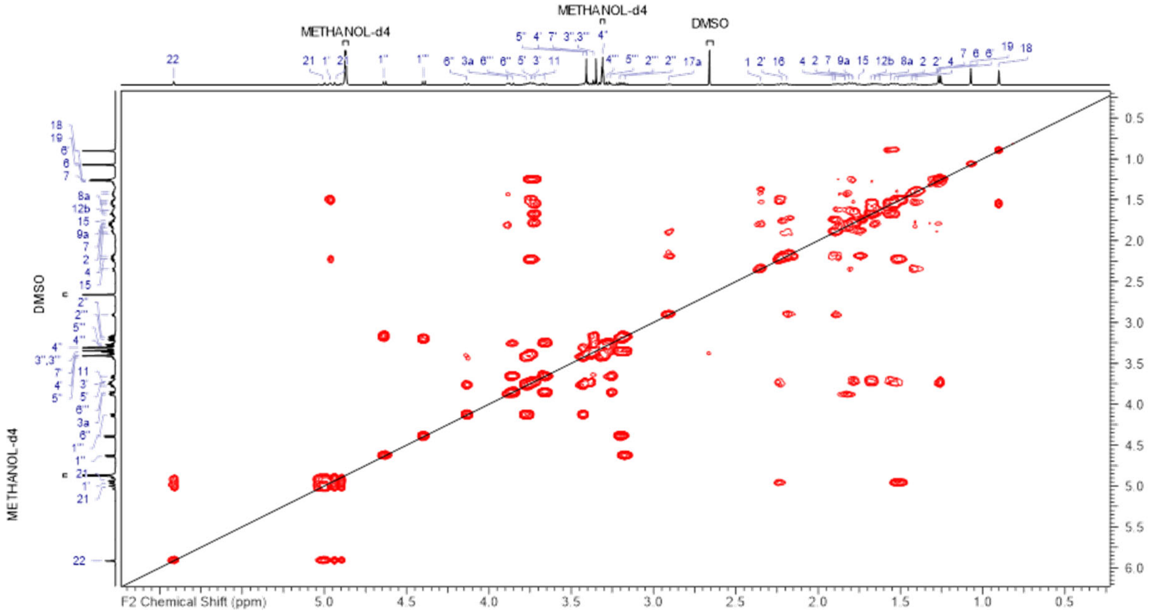

Figure S10. ^{1}H-^{1}H COSY spectrum of compound **2** (500 MHz, CD_3OD).

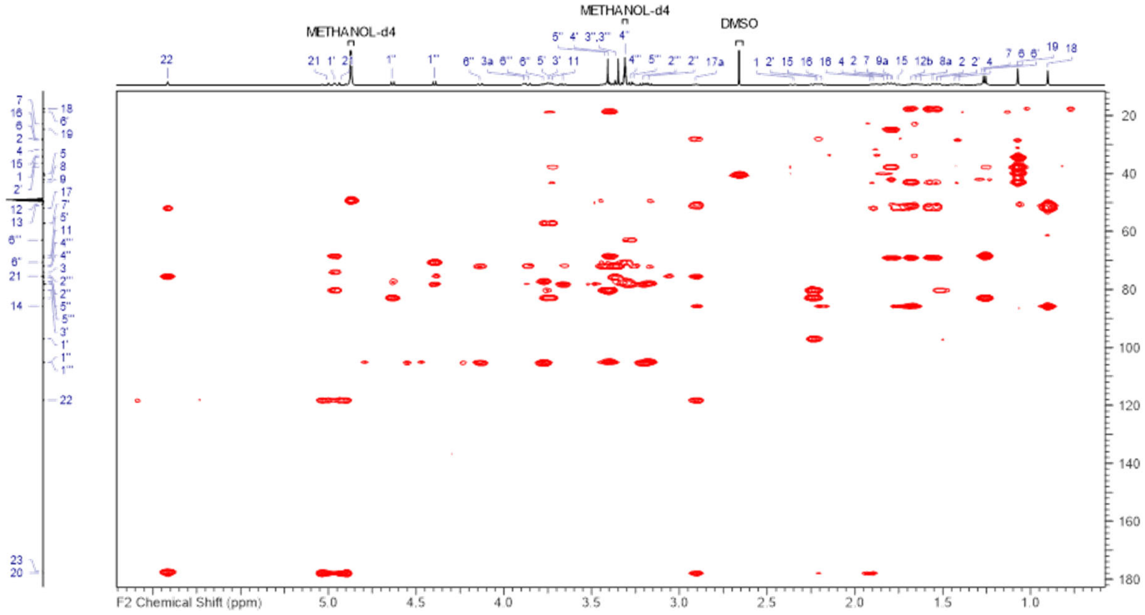

Figure S11. ^{1}H-^{13}C HMBC spectrum of compound **2** (500 MHz, CD_3OD).

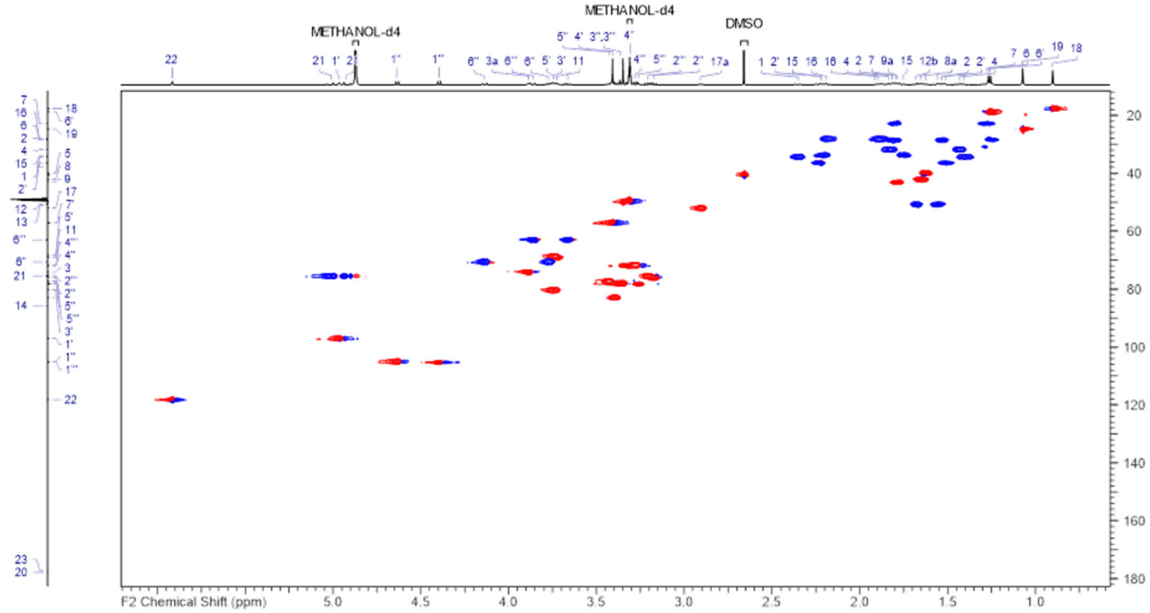

Figure S12. ^{1}H-^{13}C HSQC spectrum of compound **2** (500 MHz, CD_3OD).

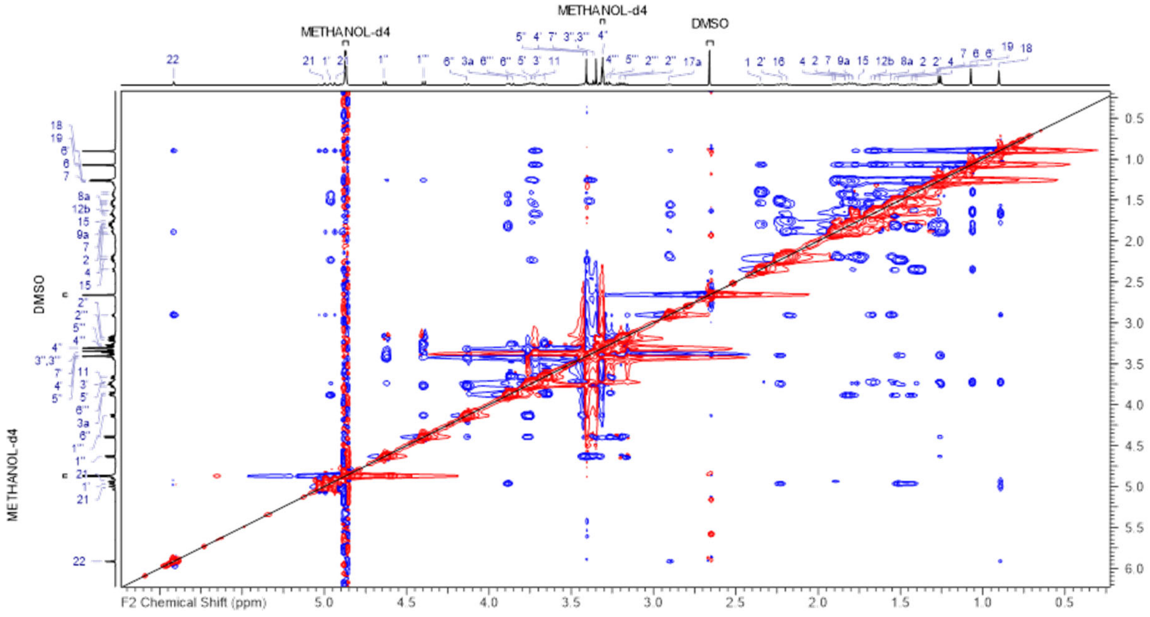

Figure S13. ^{1}H-^{1}H ROESY spectrum of compound **2** (500 MHz, CD_3OD).

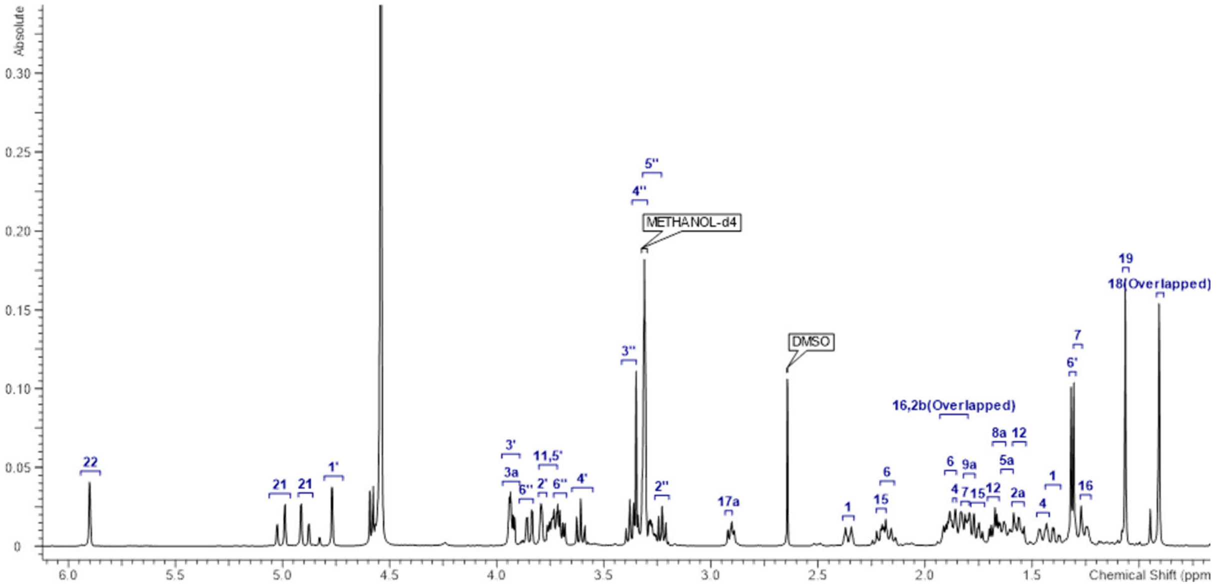

Figure S14. ^{1}H NMR spectrum of compound **3** (500 MHz, CD_3OD).

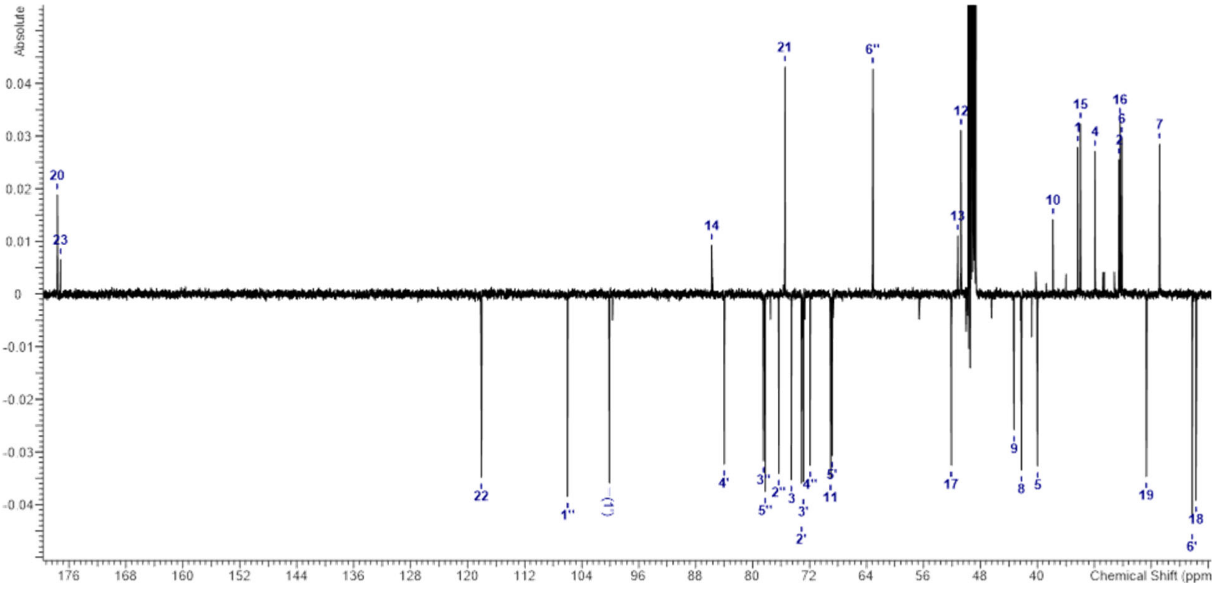

Figure S15. ^{13}C NMR spectrum of compound **3** (125 MHz, CD_3OD).

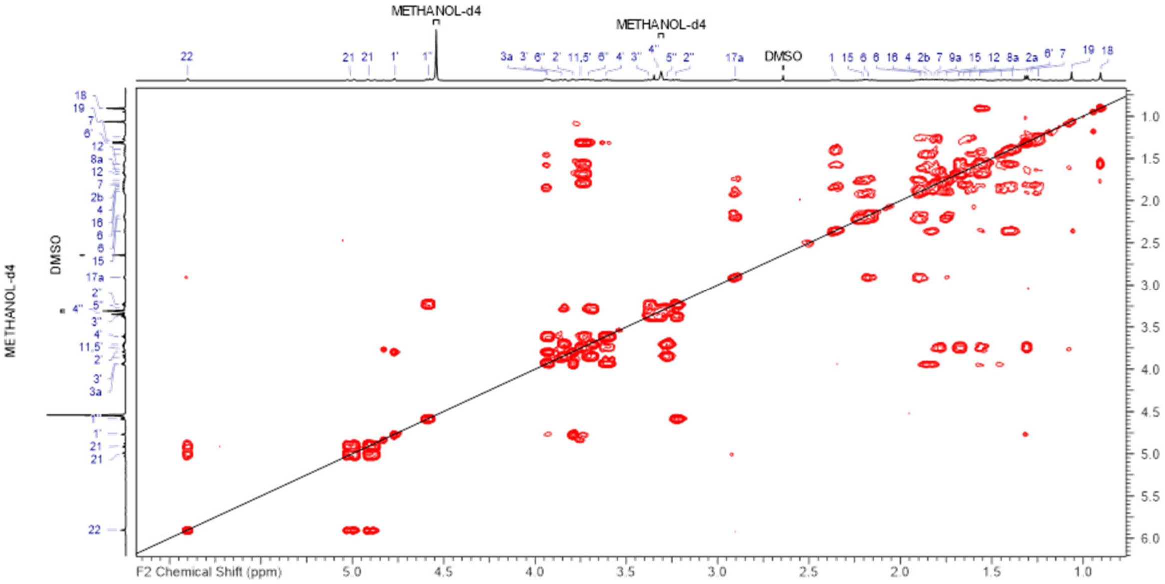

Figure S16. ^1H-^1H COSY spectrum of compound **3** (500 MHz, CD_3OD).

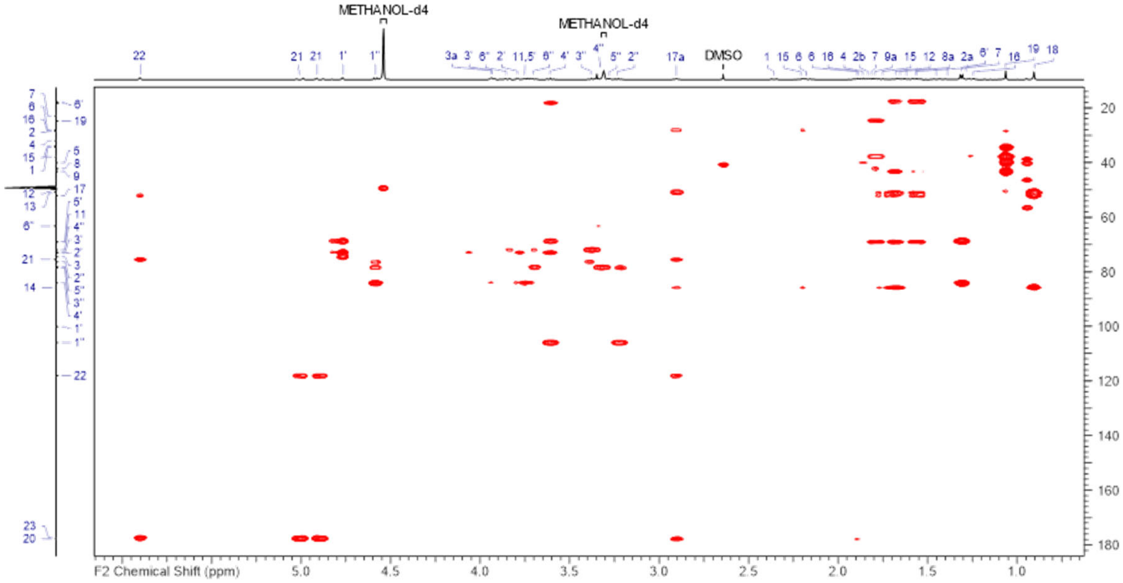

Figure S17. ^1H-^{13}C HMBC spectrum of compound **3** (500 MHz, CD_3OD).

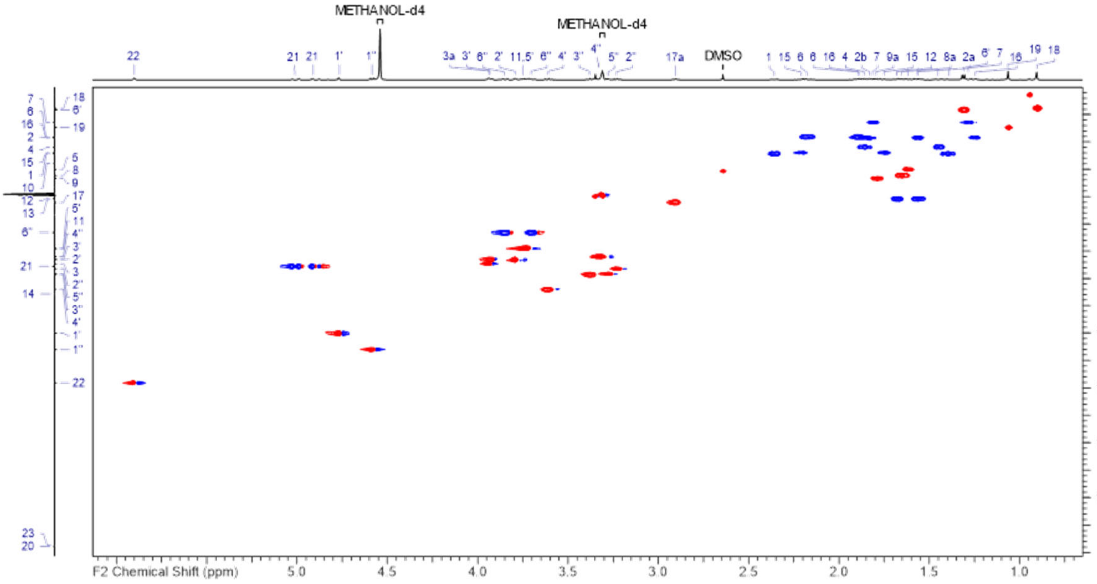

Figure S18. ^{1}H-^{13}C HSQC spectrum of compound **3** (500 MHz, CD_3OD).

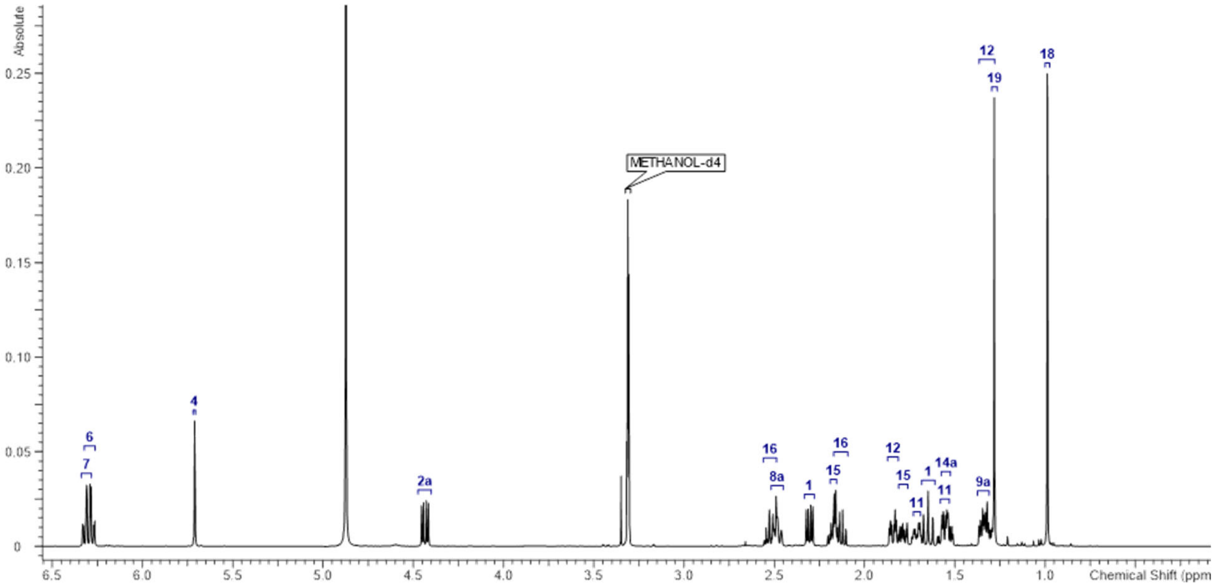

Figure S19. ^{1}H NMR spectrum of compound **4** (500 MHz, CD_3OD).

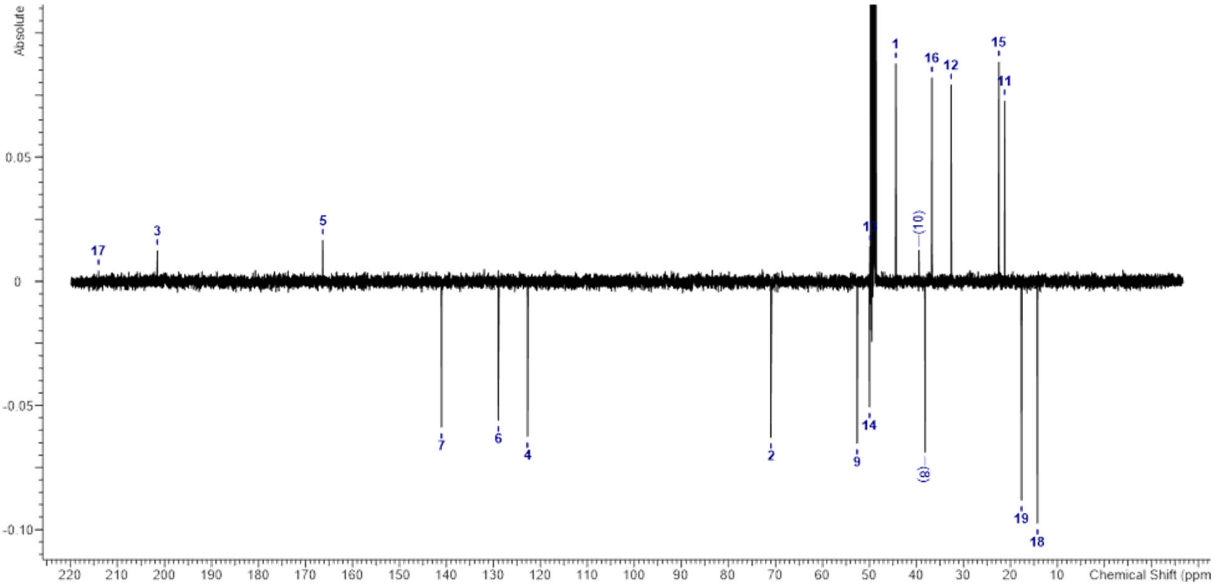

Figure S20. ^{13}C NMR spectrum of compound **4** (125 MHz, CD_3OD).

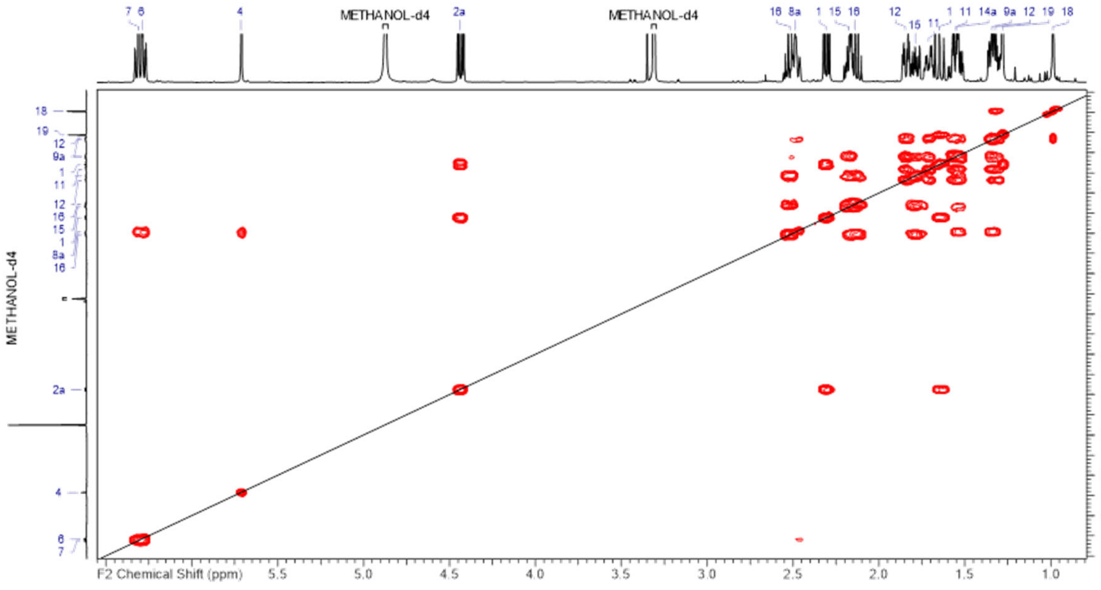

Figure S21. ^{1}H-^{1}H COSY spectrum of compound **4** (500 MHz, CD_3OD).

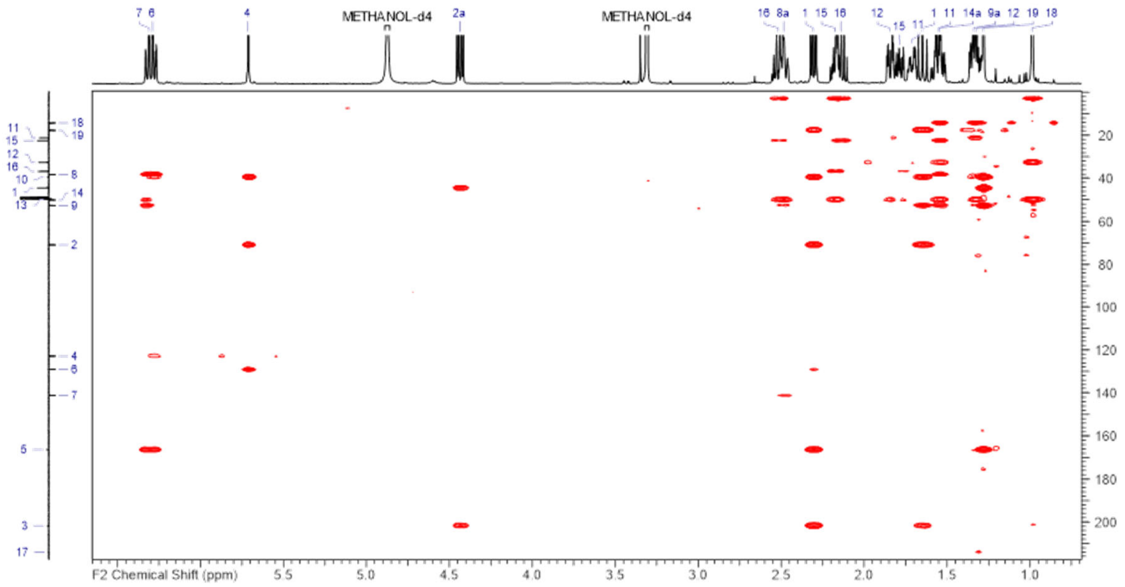

Figure S22. ^{1}H-^{13}C HMBC spectrum of compound **4** (500 MHz, CD$_3$OD).

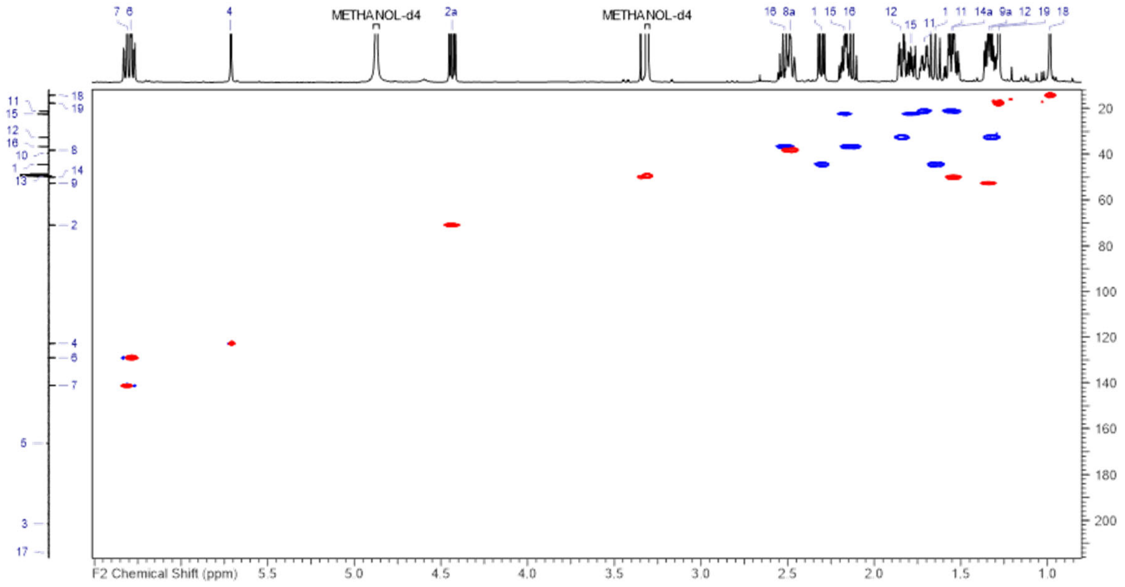

Figure S23. ^{1}H-^{13}C HSQC spectrum of compound **4** (500 MHz, CD$_3$OD).

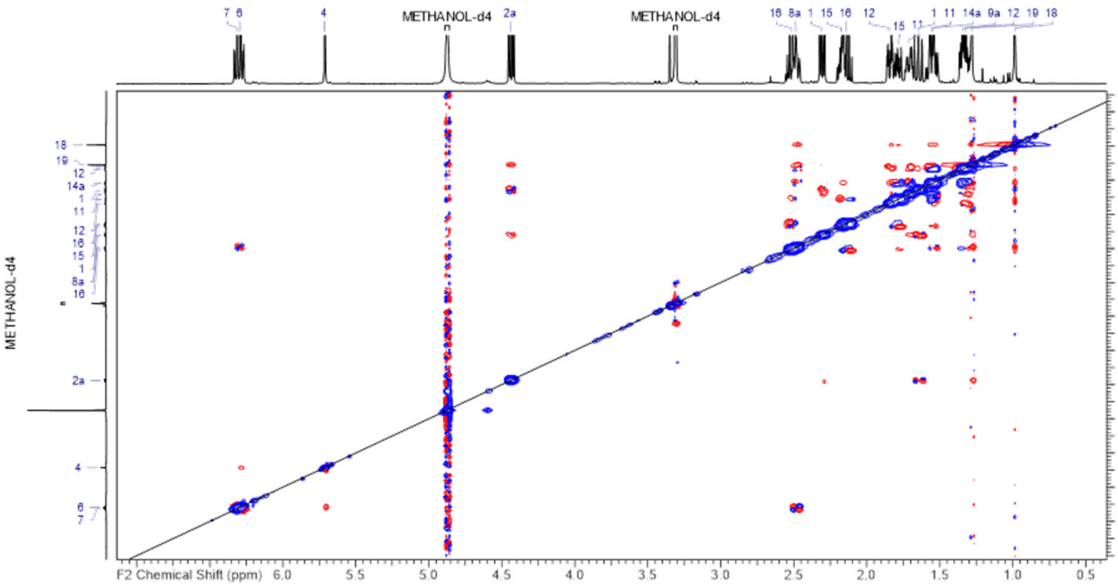

Figure S24. ^{1}H-^{1}H ROESY spectrum of compound **4** (500 MHz, CD$_3$OD).

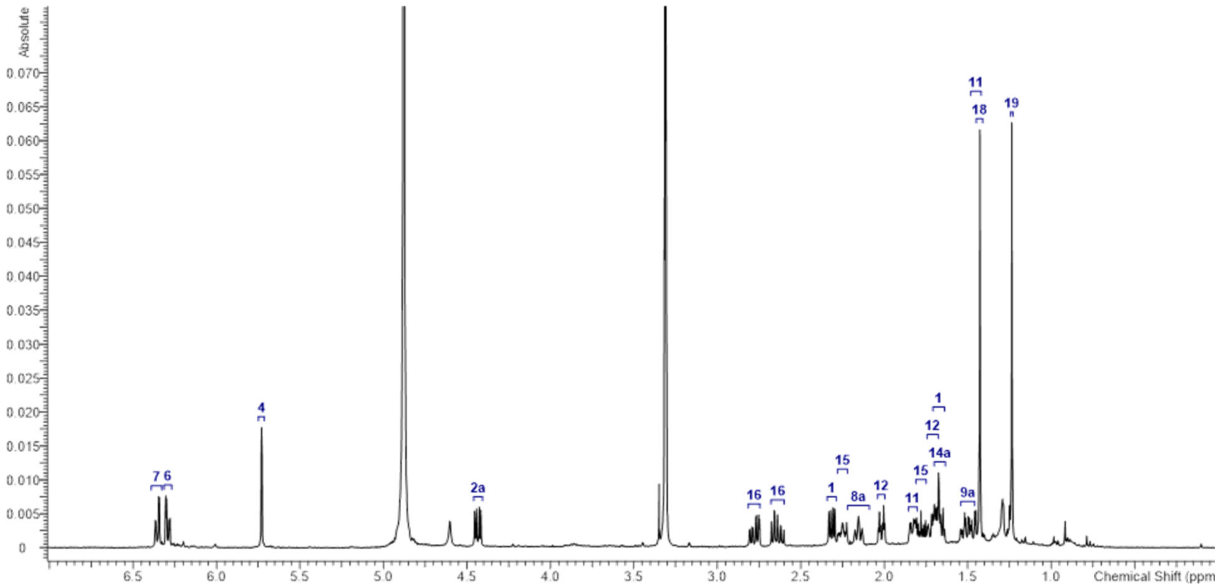

Figure S25. ^{1}H NMR spectrum of compound **5** (500 MHz, CD$_3$OD).

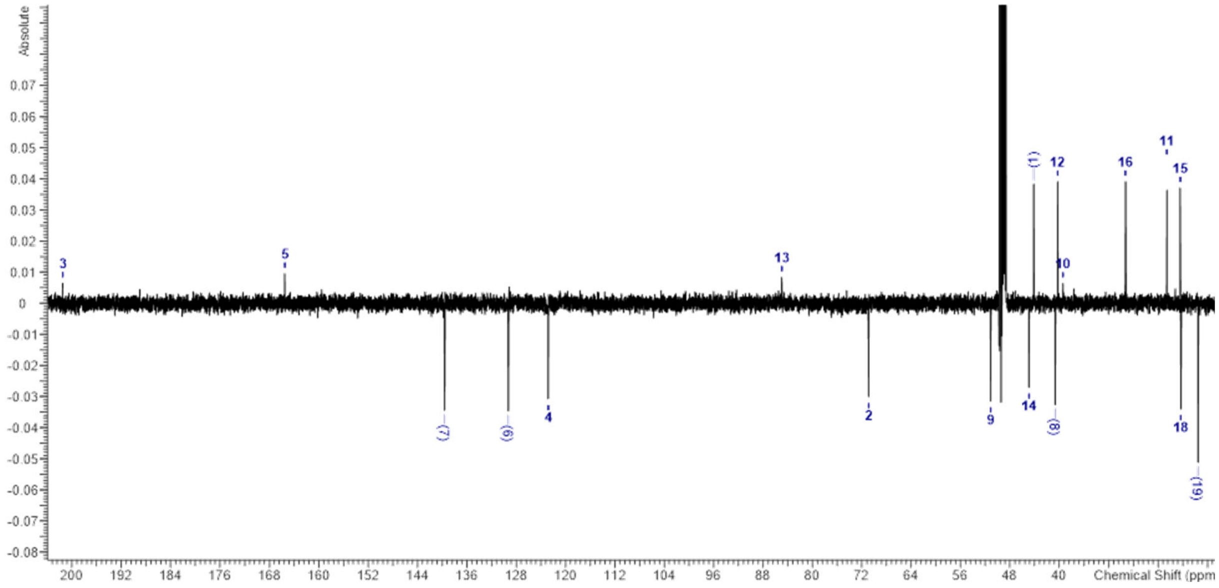

Figure S26. ^{13}C NMR spectrum of compound **5** (125 MHz, CD_3OD).

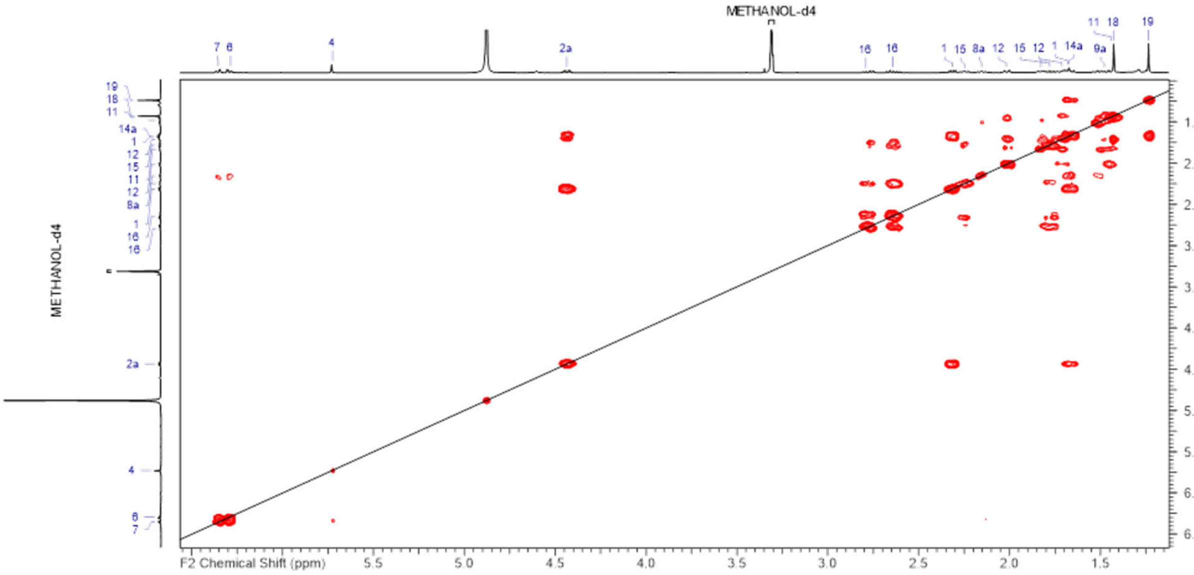

Figure S27. ^{1}H-^{1}H COSY spectrum of compound **5** (500 MHz, CD_3OD).

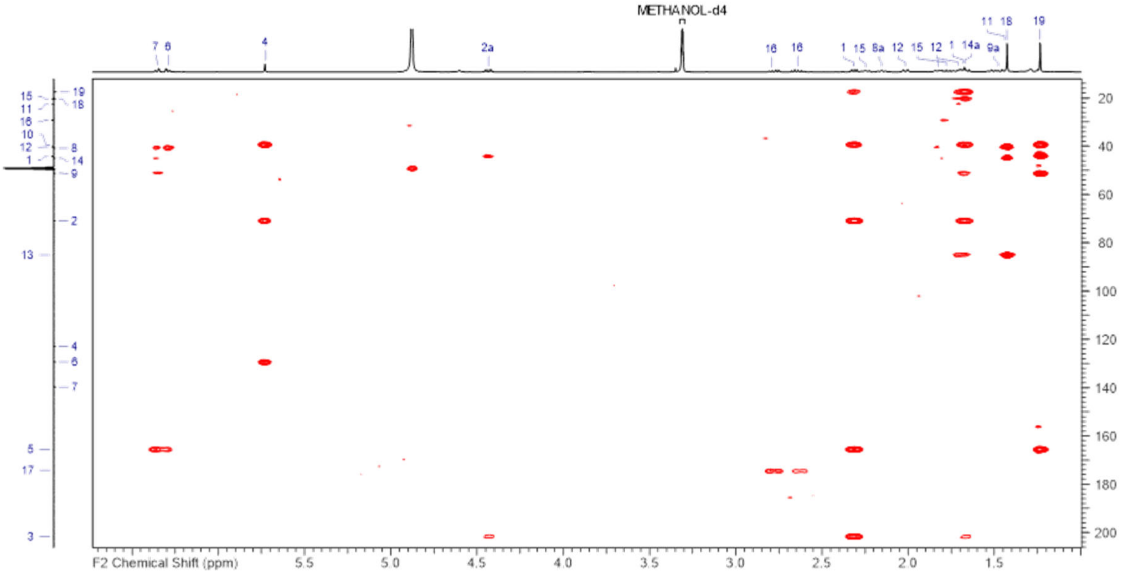

Figure S28. ^{1}H-^{13}C HMBC spectrum of compound **5** (500 MHz, CD_3OD).

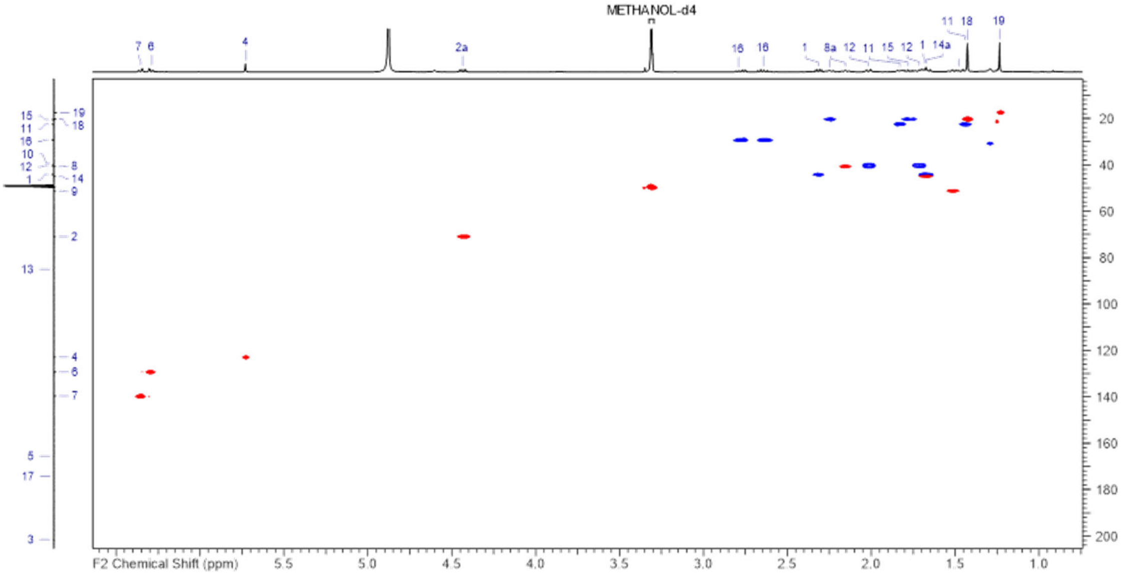

Figure S29. ^{1}H-^{13}C HSQC spectrum of compound **5** (500 MHz, CD_3OD).

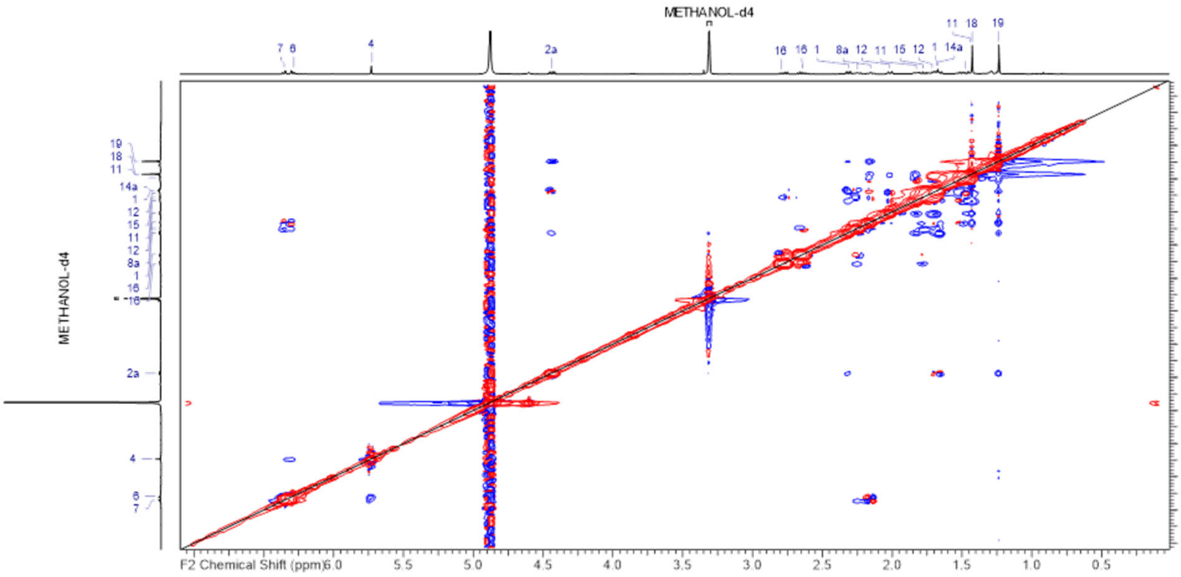

Figure S30. ^{1}H-^{1}H ROESY spectrum of compound **5** (500 MHz, CD_3OD).

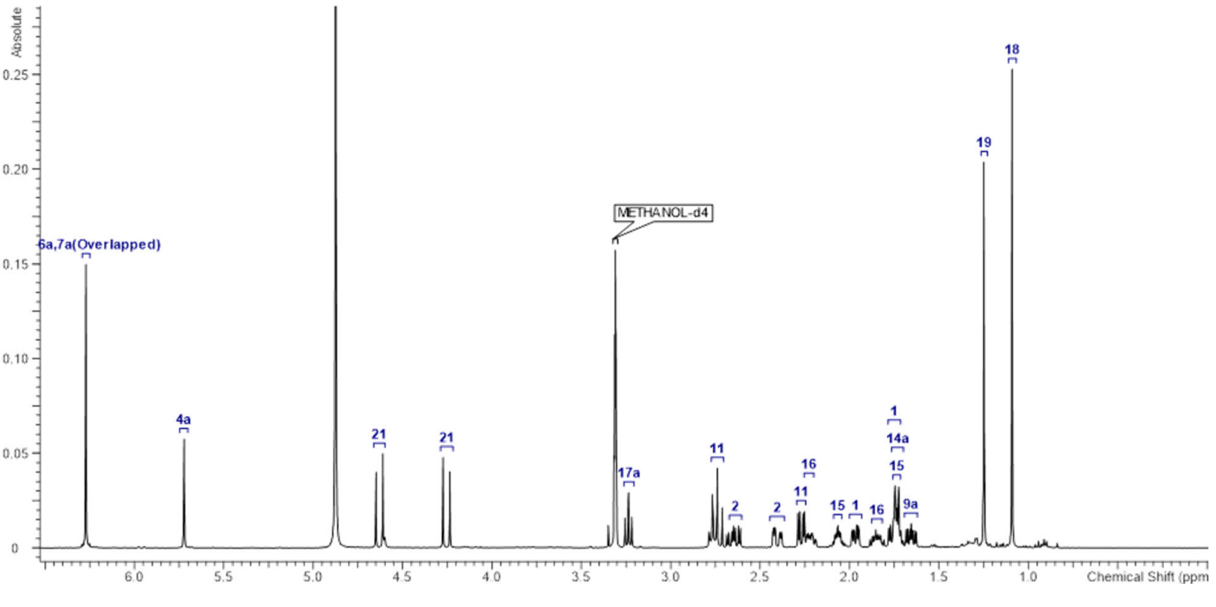

Figure S31. ^{1}H NMR spectrum of compound **6** (500 MHz, CD_3OD).

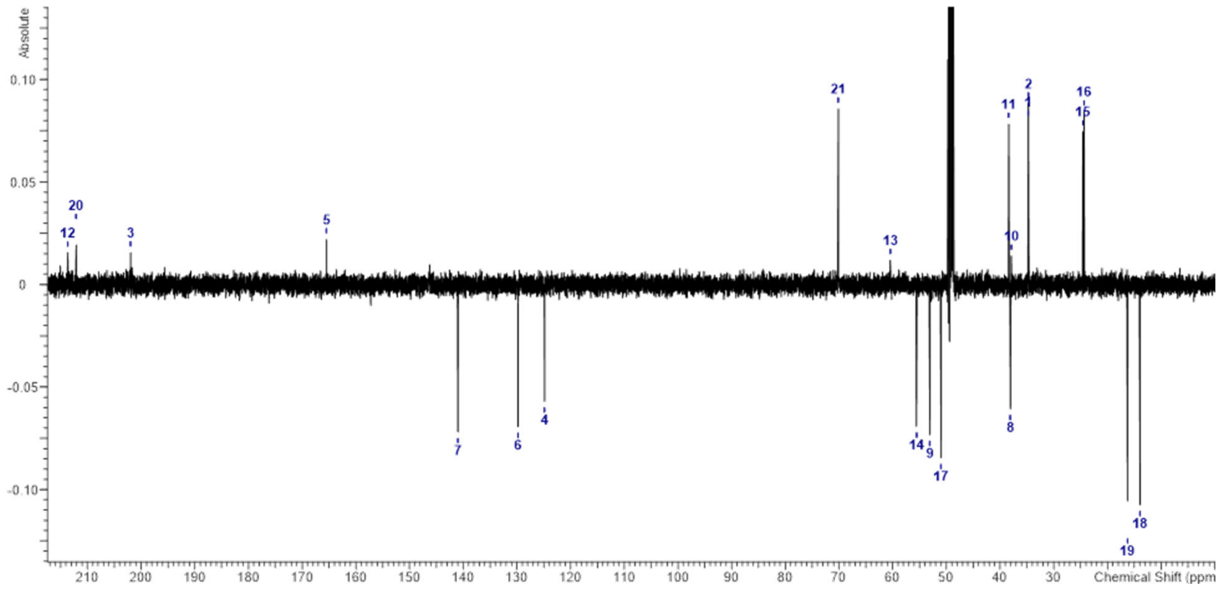

Figure S32. ^{13}C NMR spectrum of compound **6** (125 MHz, CD3OD).

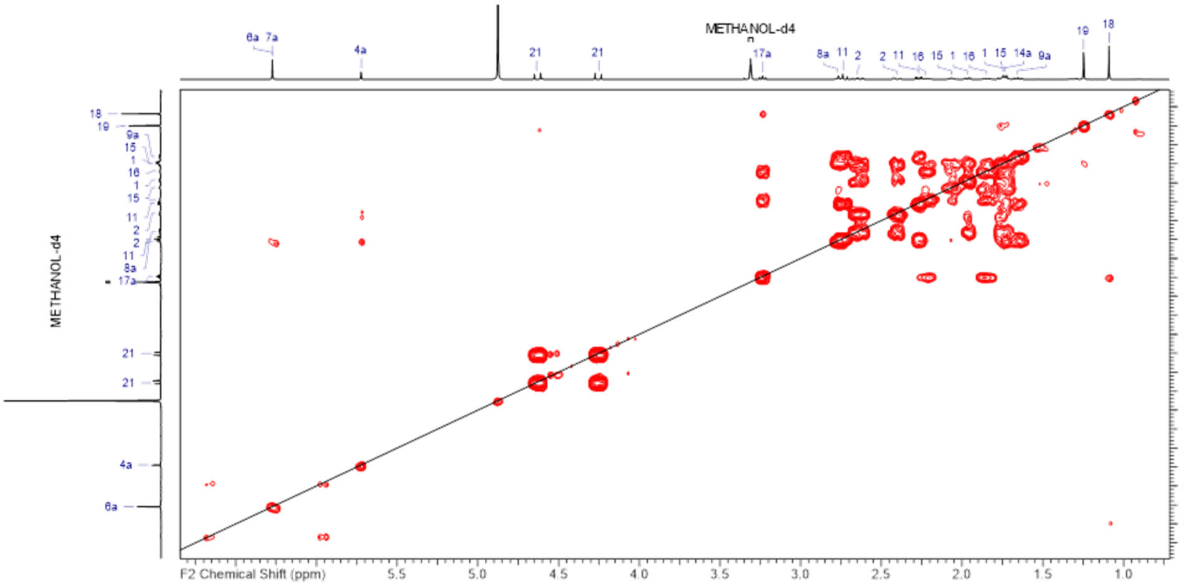

Figure S33. ^{1}H-^{1}H COSY spectrum of compound **6** (500 MHz, CD3OD).

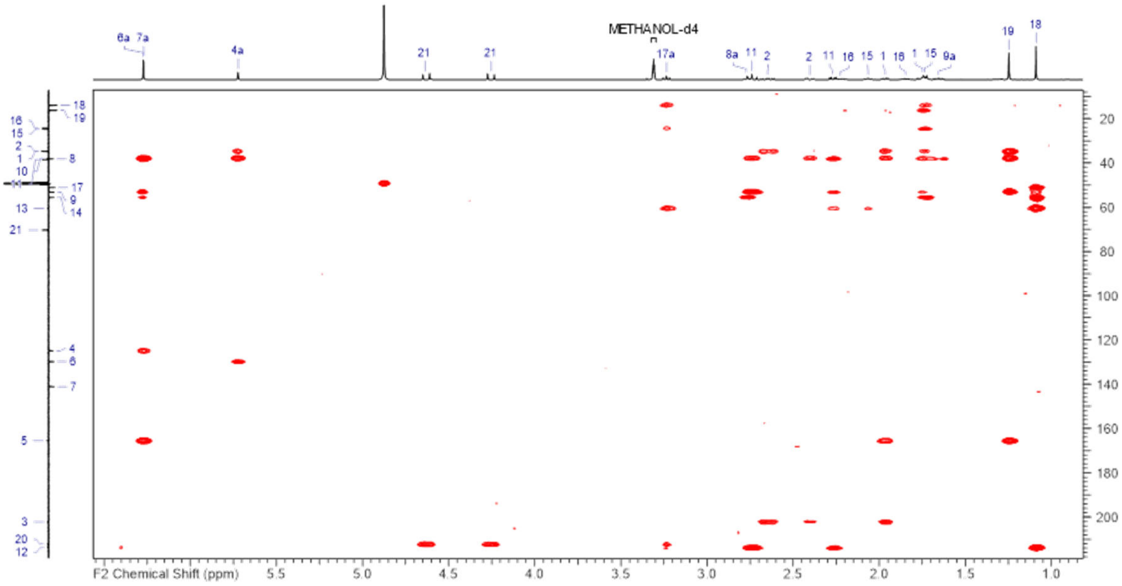

Figure S34. ^{1}H-^{13}C HMBC spectrum of compound **6** (500 MHz, CD_3OD).

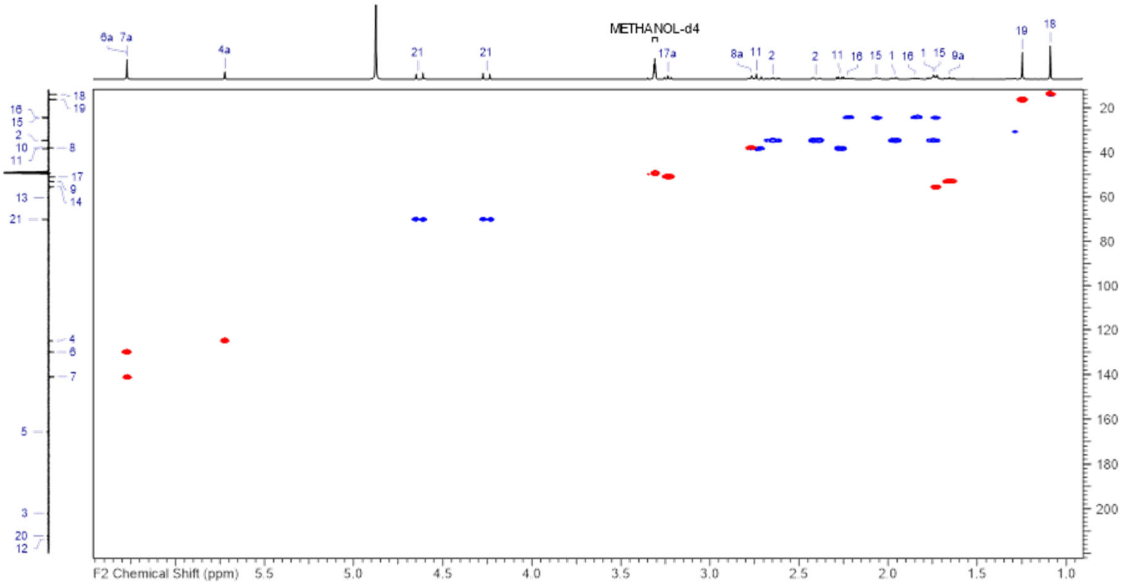

Figure S35. ^{1}H-^{13}C HSQC spectrum of compound **6** (500 MHz, CD_3OD).

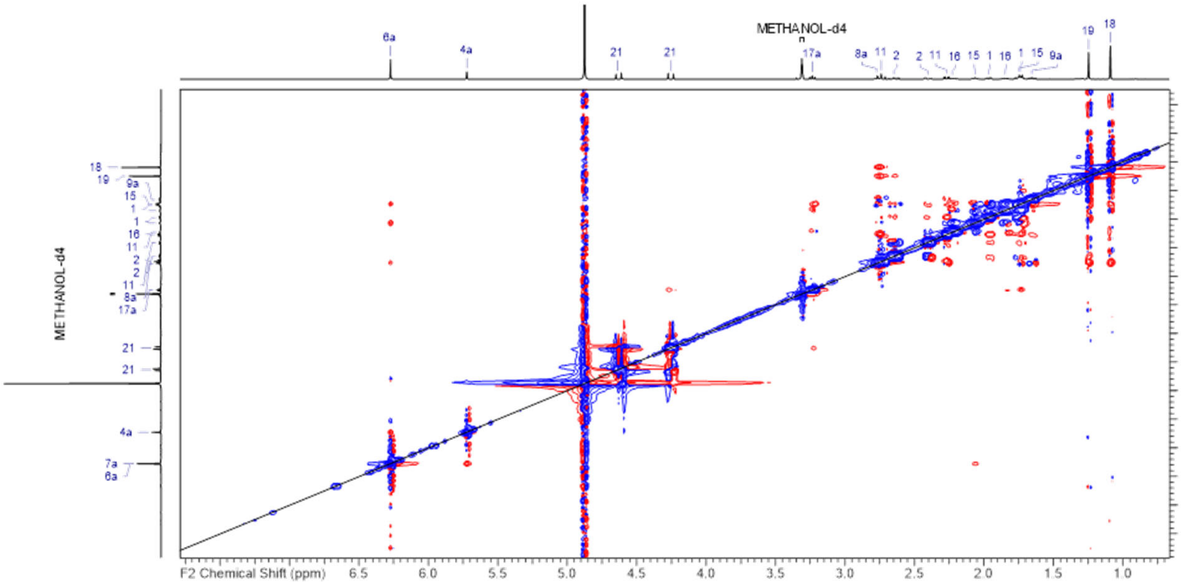

Figure S36. ^{1}H-^{1}H ROESY spectrum of compound **6** (500 MHz, CD_3OD).

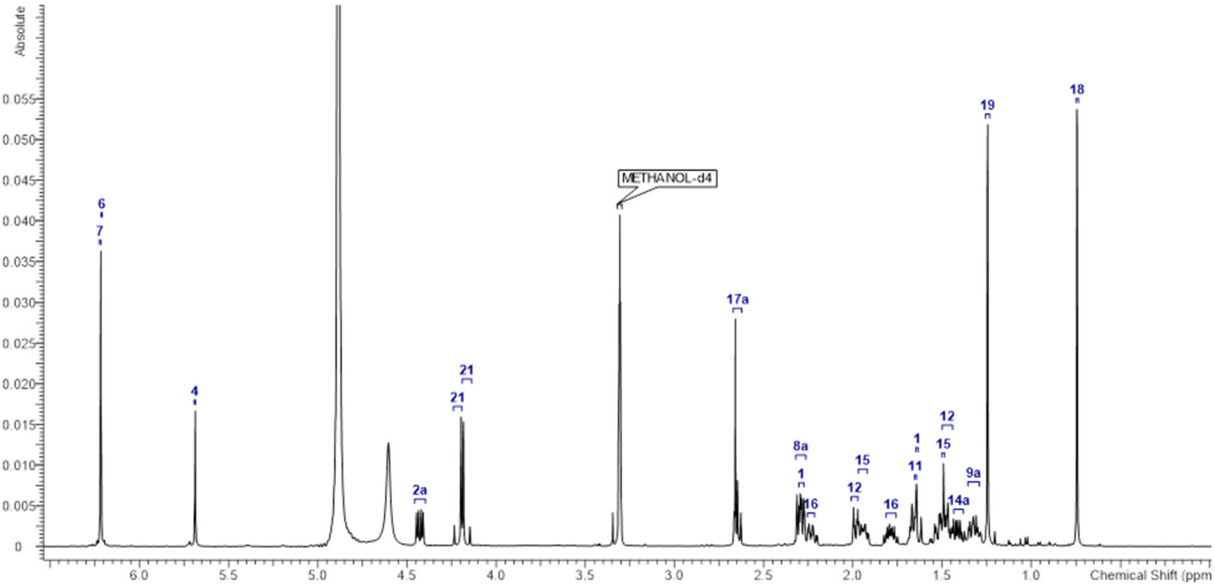

Figure S37. ^{1}H NMR spectrum of compound **7** (500 MHz, CD_3OD).

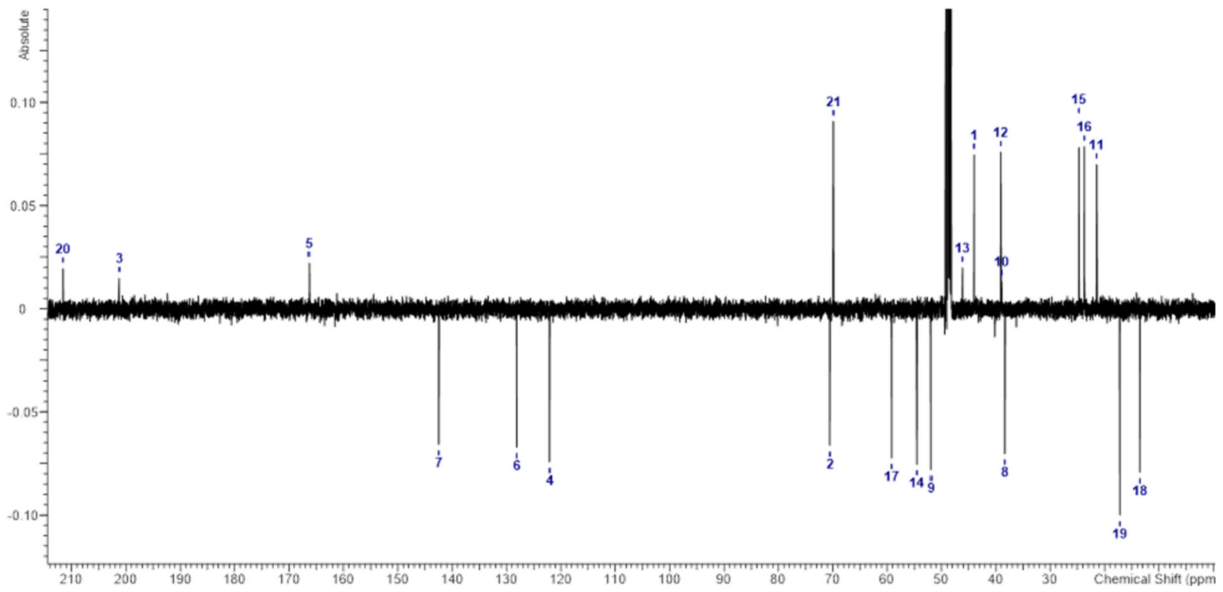

Figure S38. ^{13}C NMR spectrum of compound **7** (125 MHz, CD_3OD).

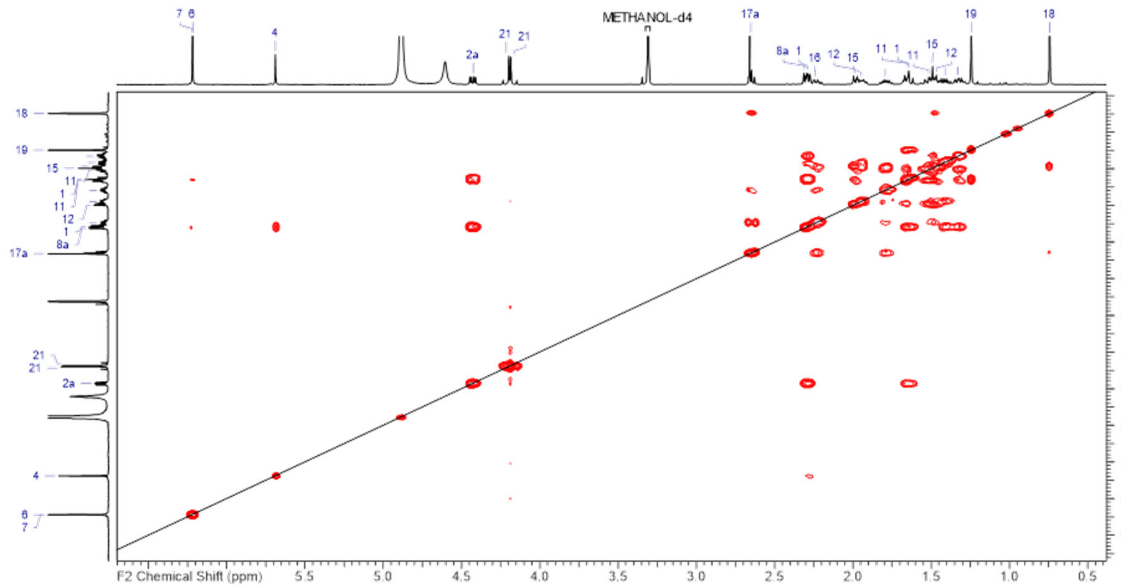

Figure S39. ^{1}H-^{1}H COSY spectrum of compound **7** (500 MHz, CD_3OD).

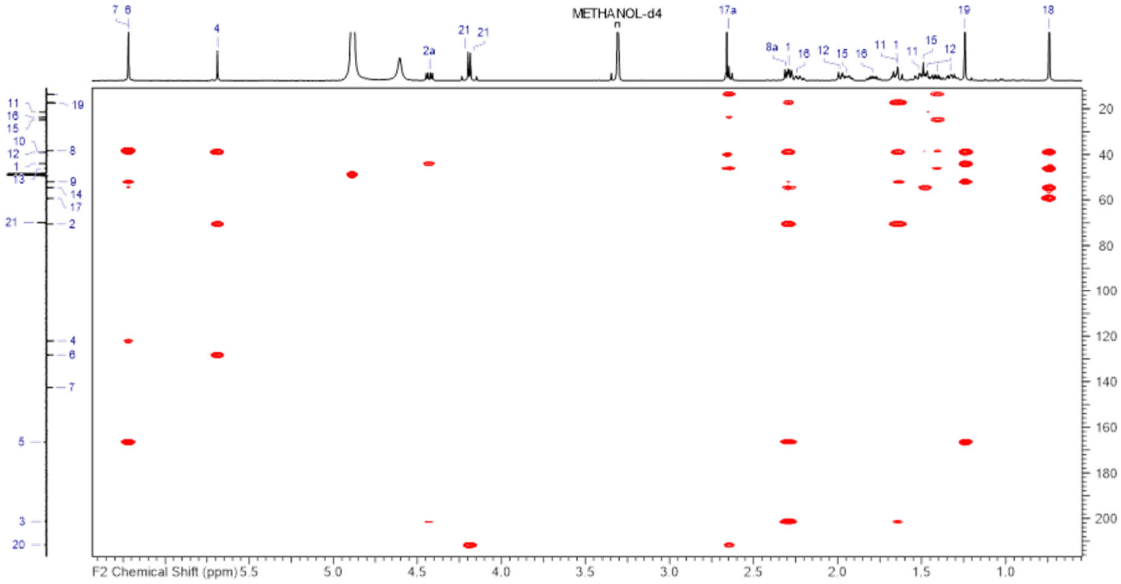

Figure S40. ^{1}H-^{13}C HMBC spectrum of compound **7** (500 MHz, CD_3OD).

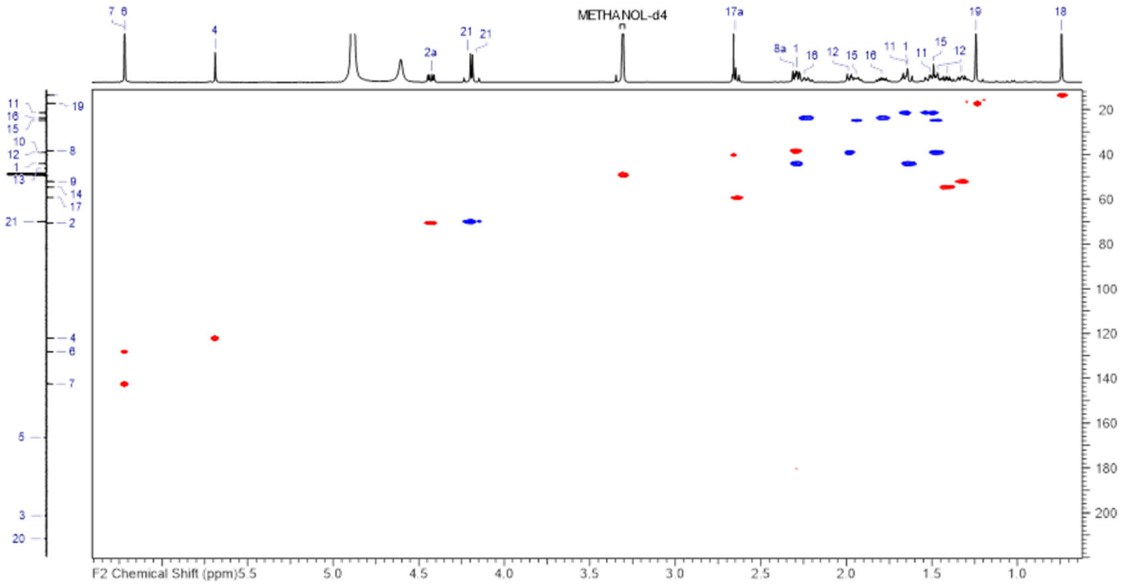

Figure S41. ^{1}H-^{13}C HSQC spectrum of compound **7** (500 MHz, CD_3OD).

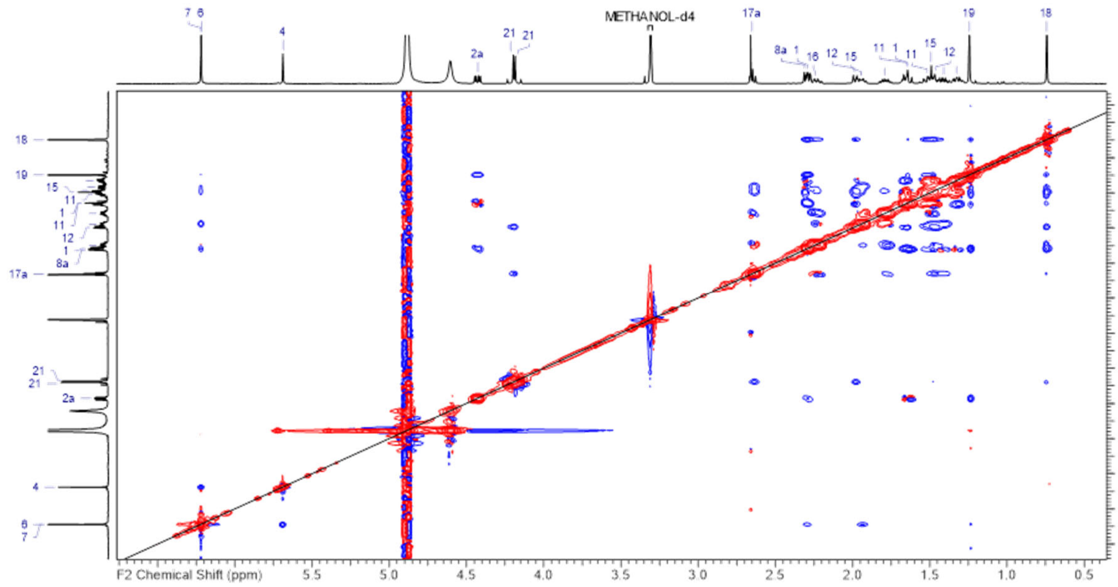

Figure S42. ^{1}H-^{1}H ROESY spectrum of compound **7** (500 MHz, CD_3OD).

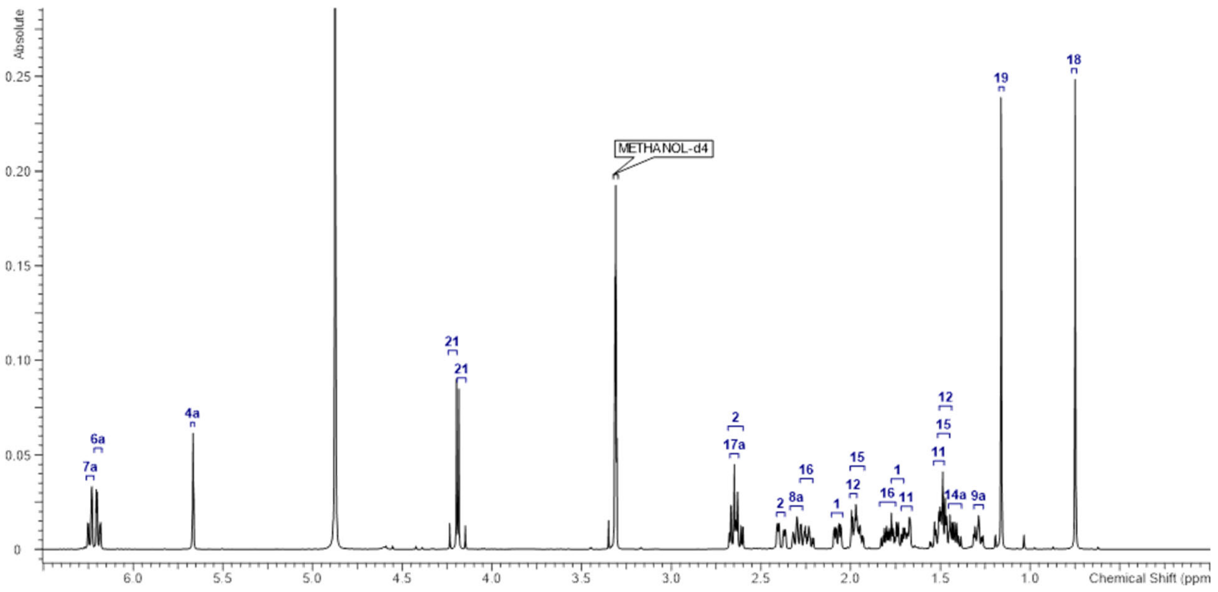

Figure S43. ^{1}H NMR spectrum of compound **8** (500 MHz, CD_3OD).

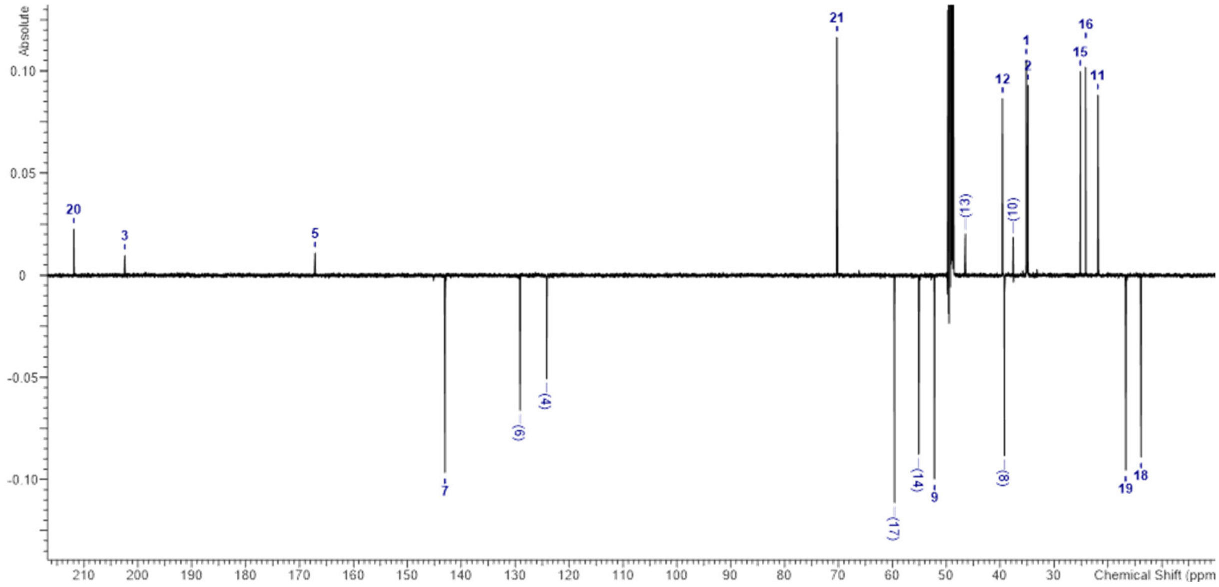

Figure S44. ^{13}C NMR spectrum of compound **8** (125 MHz, CD_3OD).

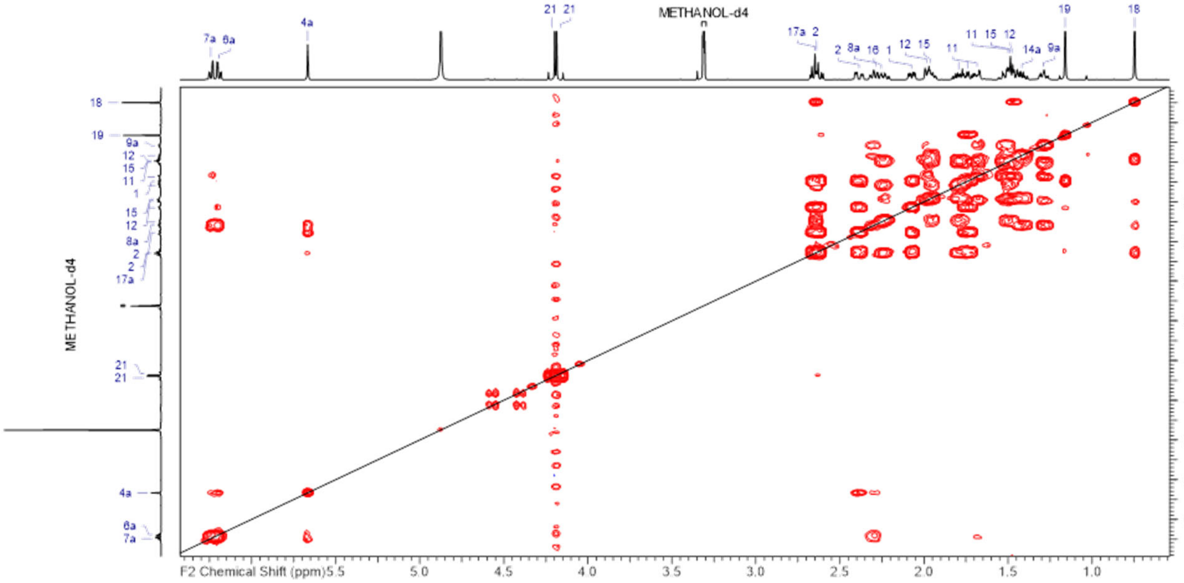

Figure S45. ^{1}H-^{1}H COSY spectrum of compound **8** (500 MHz, CD_3OD).

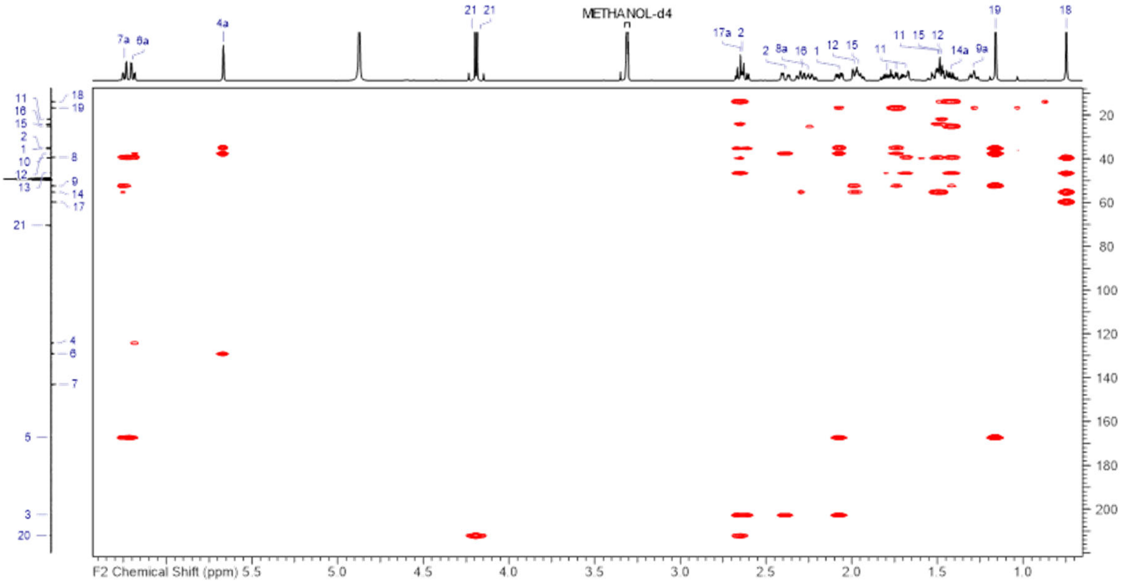

Figure S46. ^{1}H-^{13}C HMBC spectrum of compound **8** (500 MHz, CD_3OD).

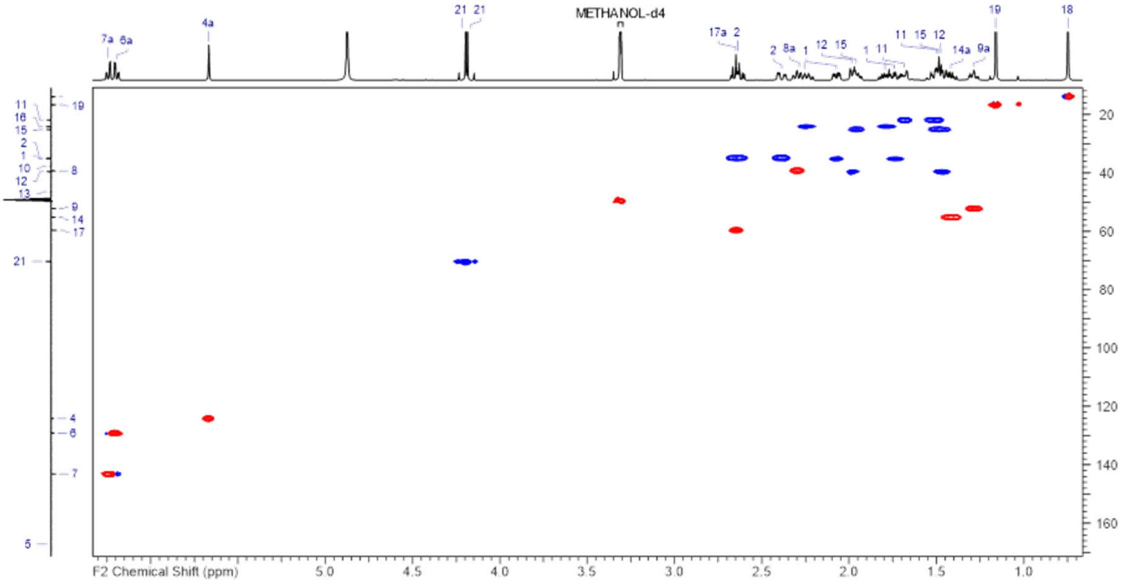

Figure S47. ^{1}H-^{13}C HSQC spectrum of compound **8** (500 MHz, CD_3OD).

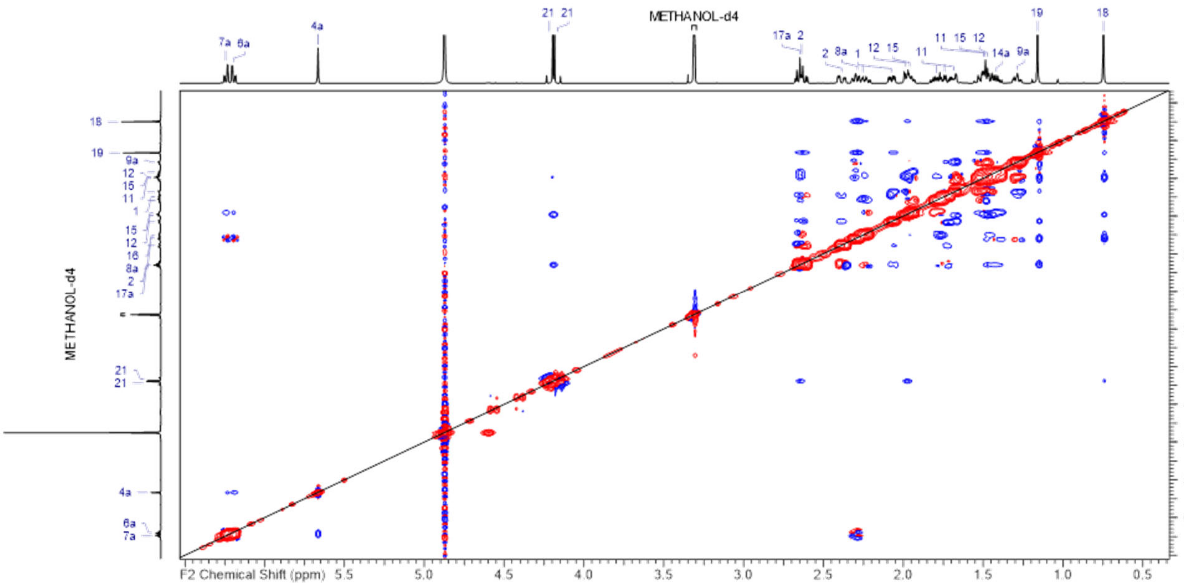

Figure S48. ^{1}H-^{1}H ROESY spectrum of compound **8** (500 MHz, CD_3OD).

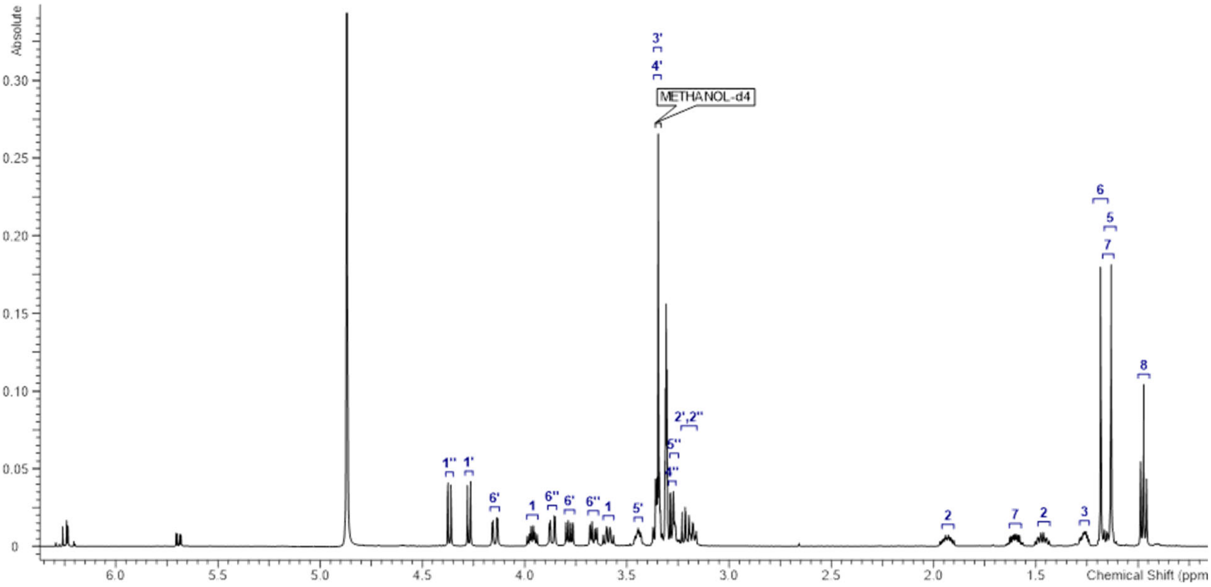

Figure S49. ^{1}H NMR spectrum of compound **9** (500 MHz, CD_3OD).

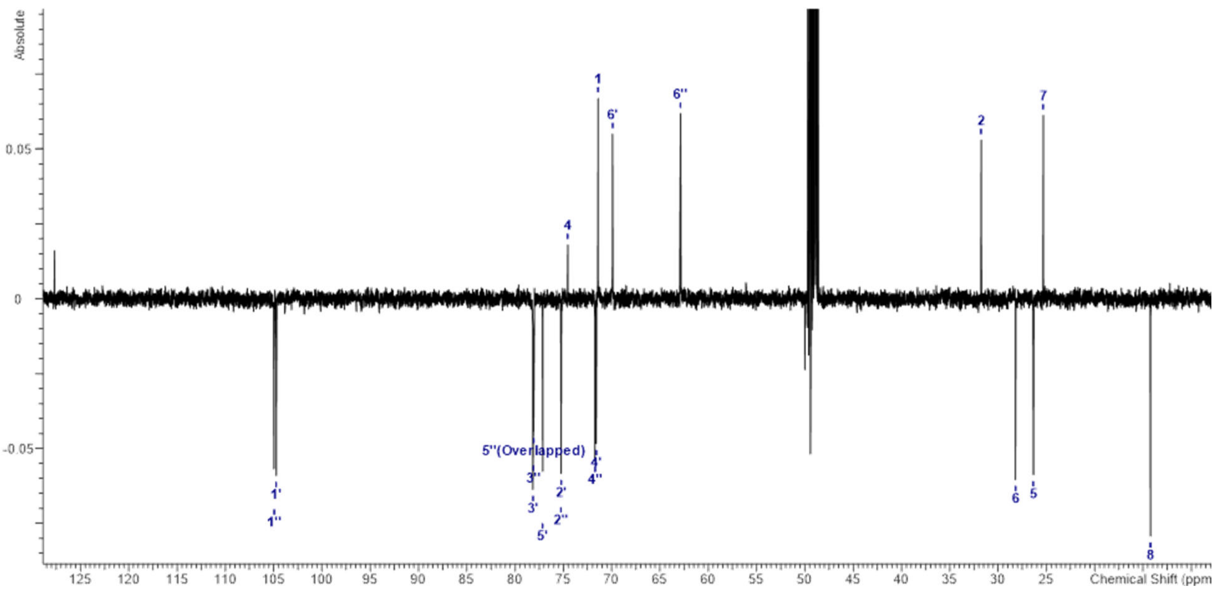

Figure S50. ^{13}C NMR spectrum of compound **9** (125 MHz, CD_3OD).

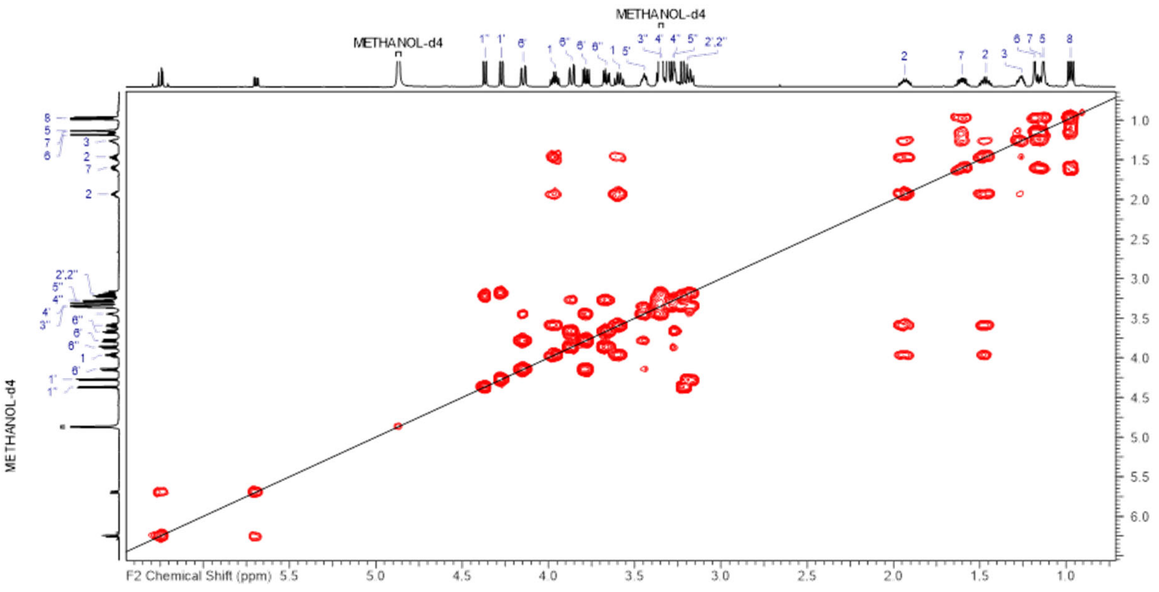

Figure S51. ^{1}H-^{1}H COSY spectrum of compound **9** (500 MHz, CD_3OD).

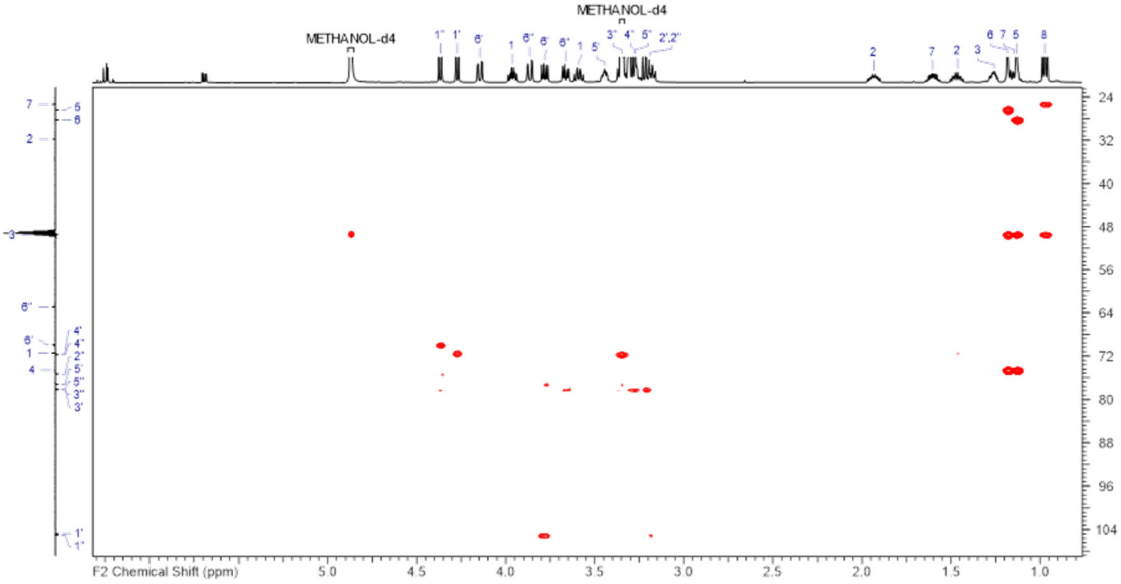

Figure S52. ^1H-^{13}C HMBC spectrum of compound **9** (500 MHz, CD_3OD).

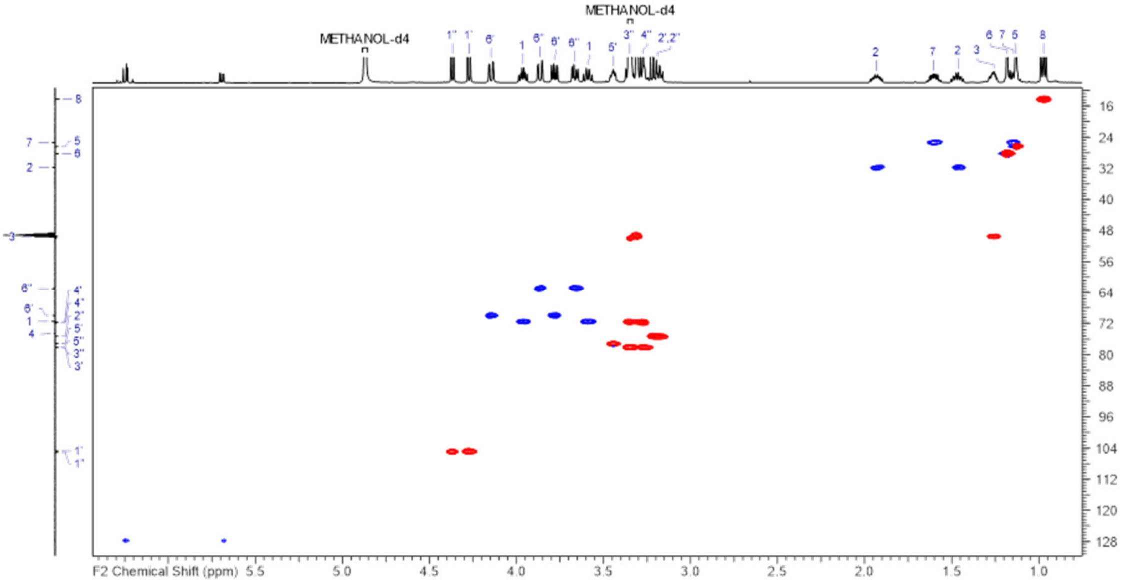

Figure S53. ^1H-^{13}C HSQC spectrum of compound **9** (500 MHz, CD_3OD).

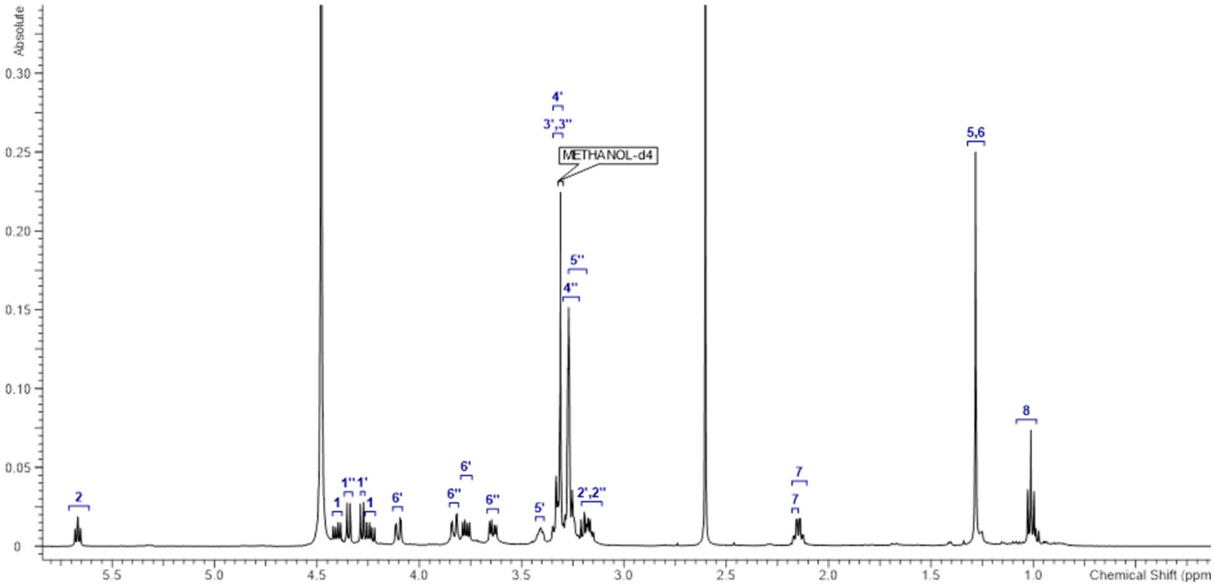

Figure S54. ^{1}H NMR spectrum of compound **10** (500 MHz, CD_3OD).

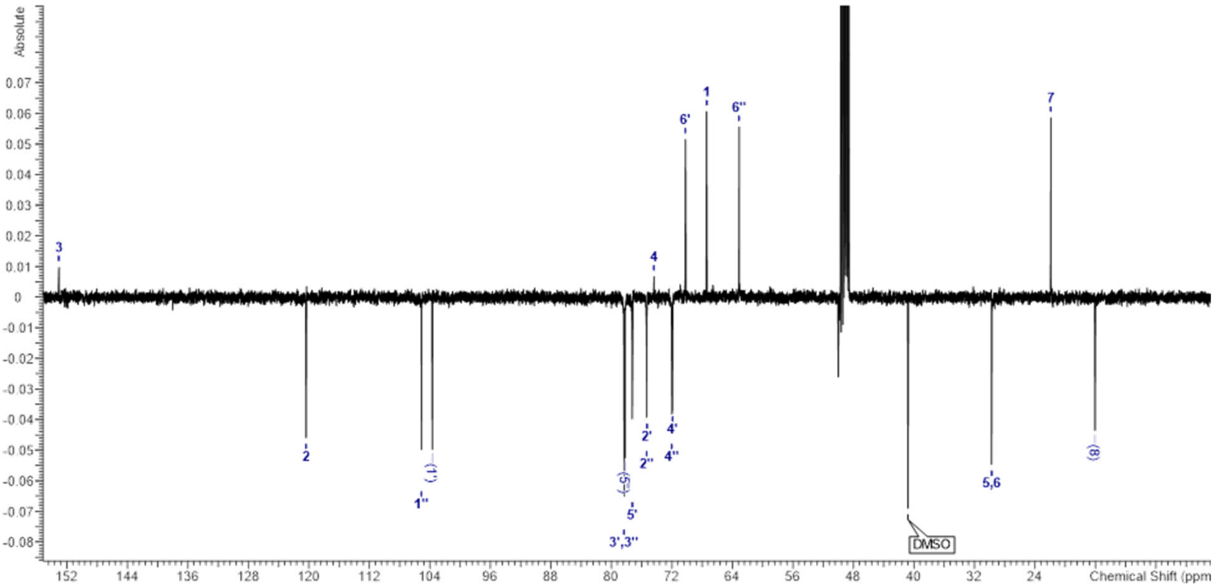

Figure S55. ^{13}C NMR spectrum of compound **10** (125 MHz, CD_3OD).

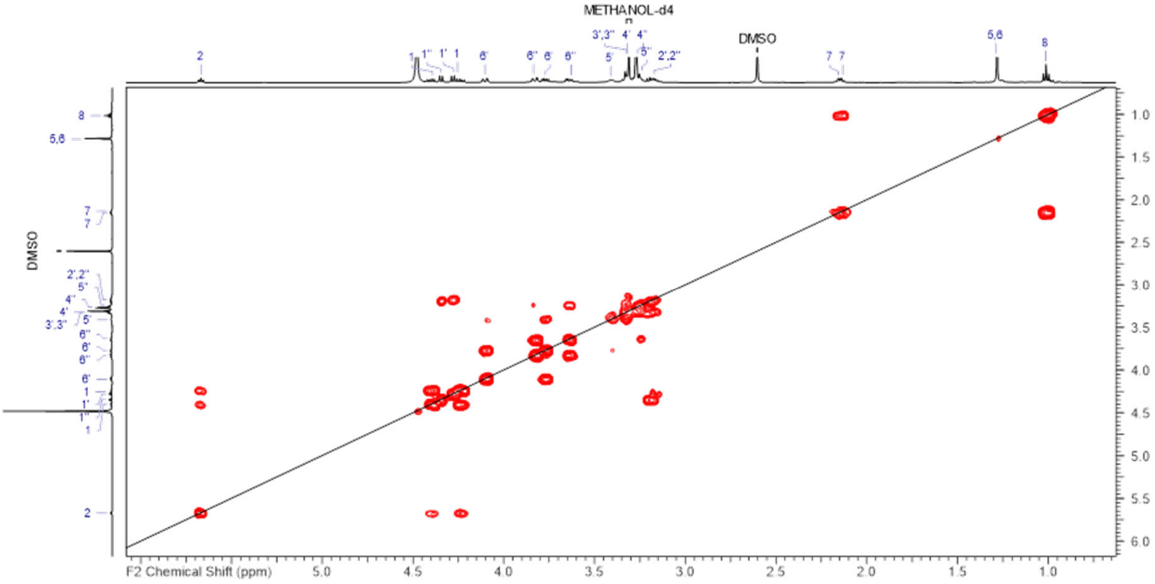

Figure S56. ^{1}H-^{1}H COSY spectrum of compound **10** (500 MHz, CD_3OD).

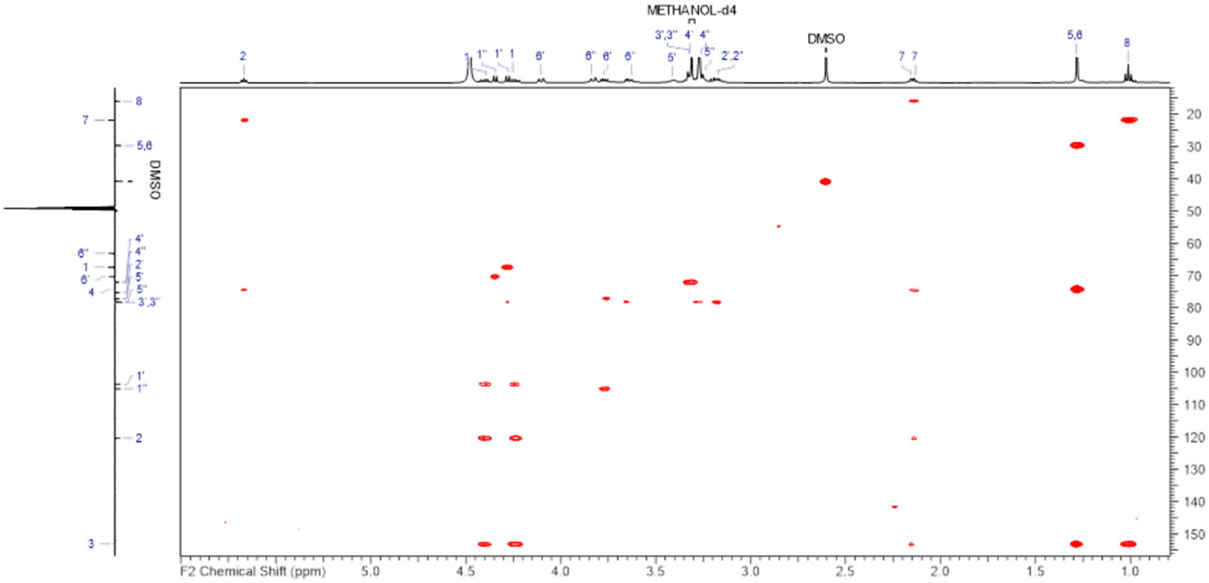

Figure S57. ^{1}H-^{13}C HMBC spectrum of compound **10** (500 MHz, CD_3OD).

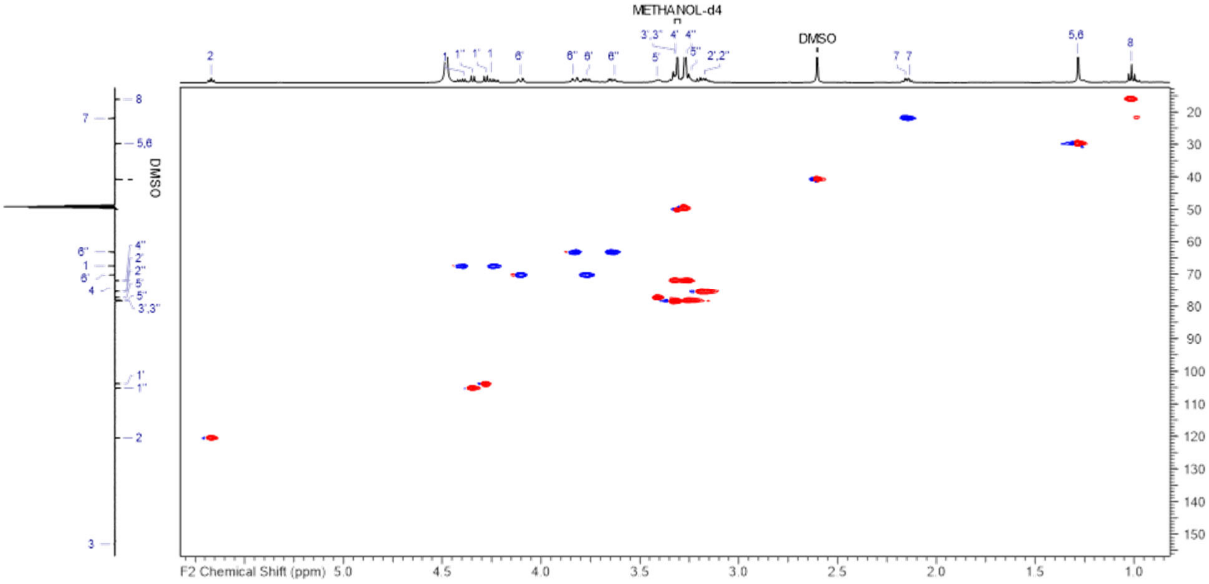

Figure S58. ^{1}H-^{13}C HSQC spectrum of compound **10** (500 MHz, CD_3OD).

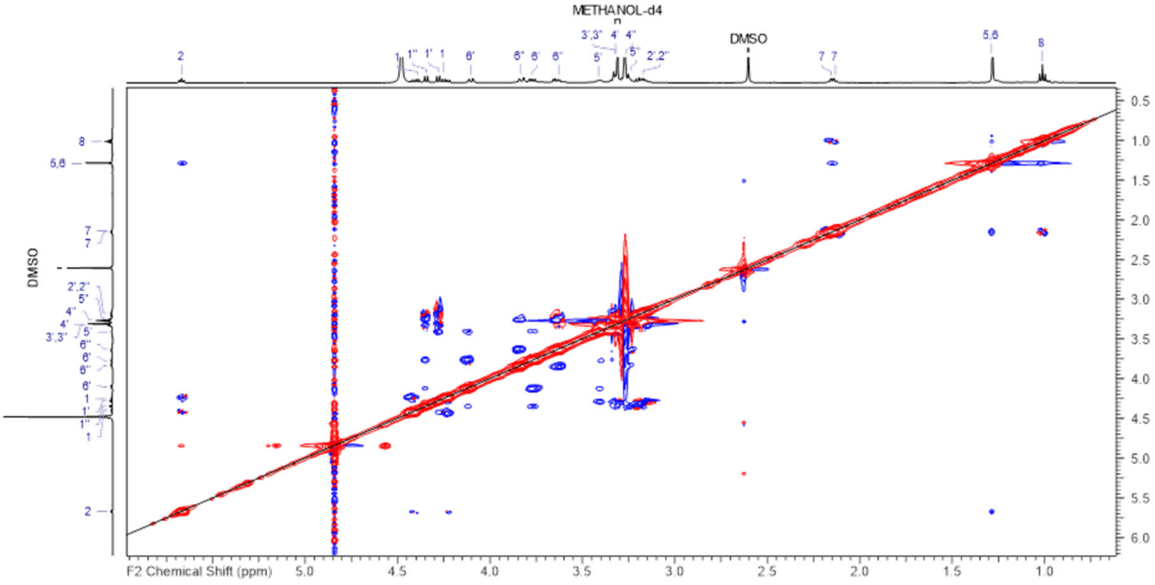

Figure S59. ^{1}H-^{1}H ROESY spectrum of compound **10** (500 MHz, CD_3OD).

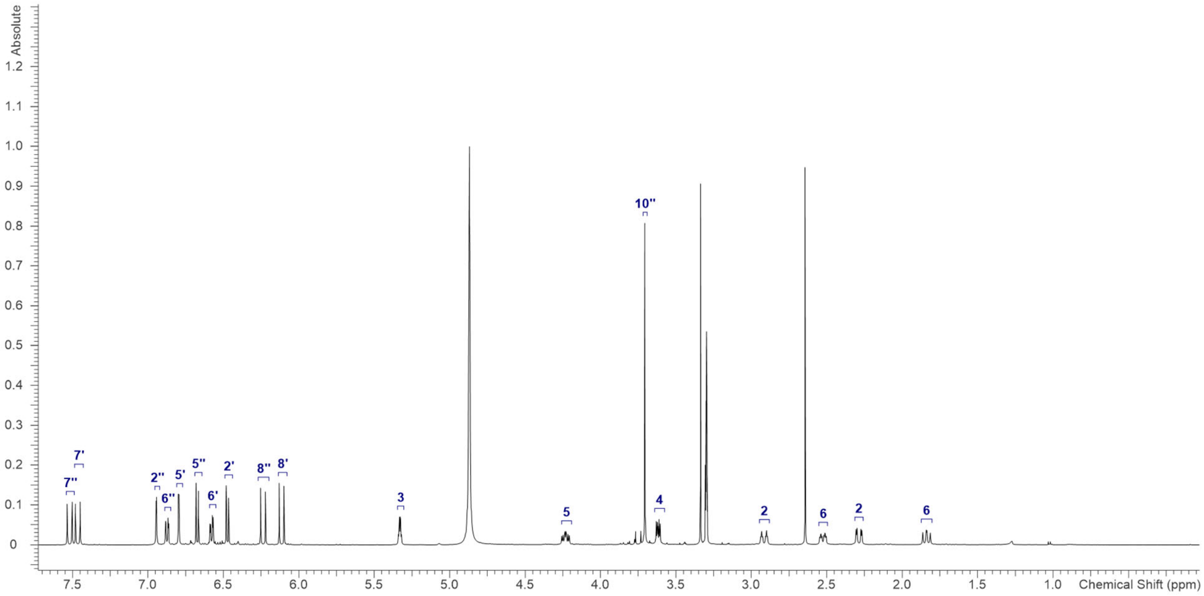

Figure S60. ^{1}H NMR spectrum of compound **11** (500 MHz, CD$_3$OD).

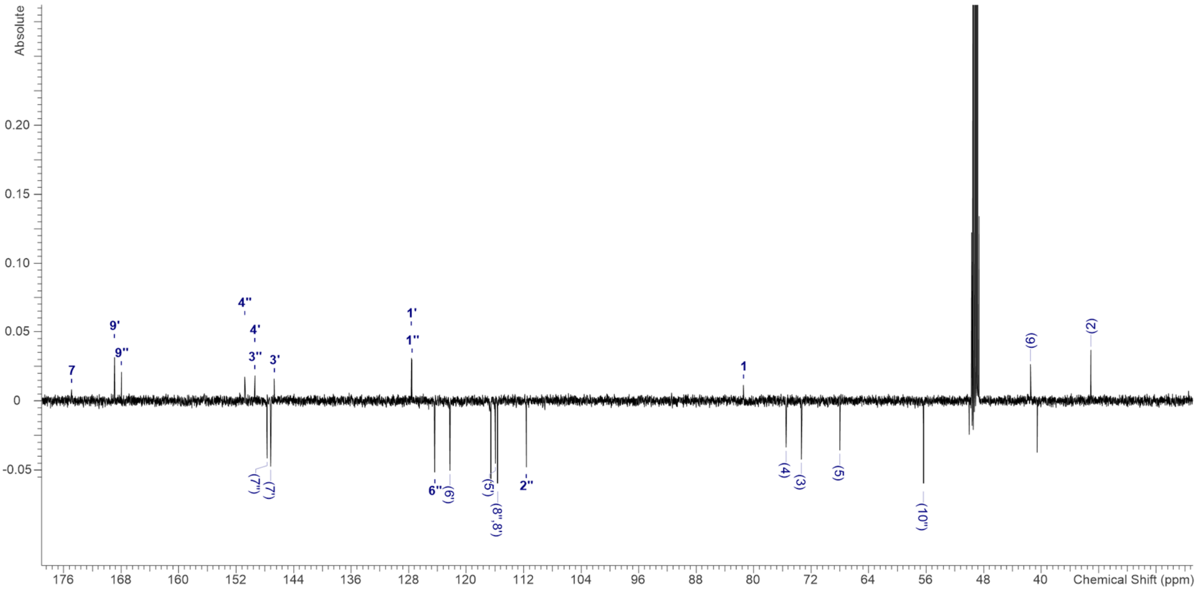

Figure S61. ^{13}C NMR spectrum of compound **11** (125 MHz, CD$_3$OD).

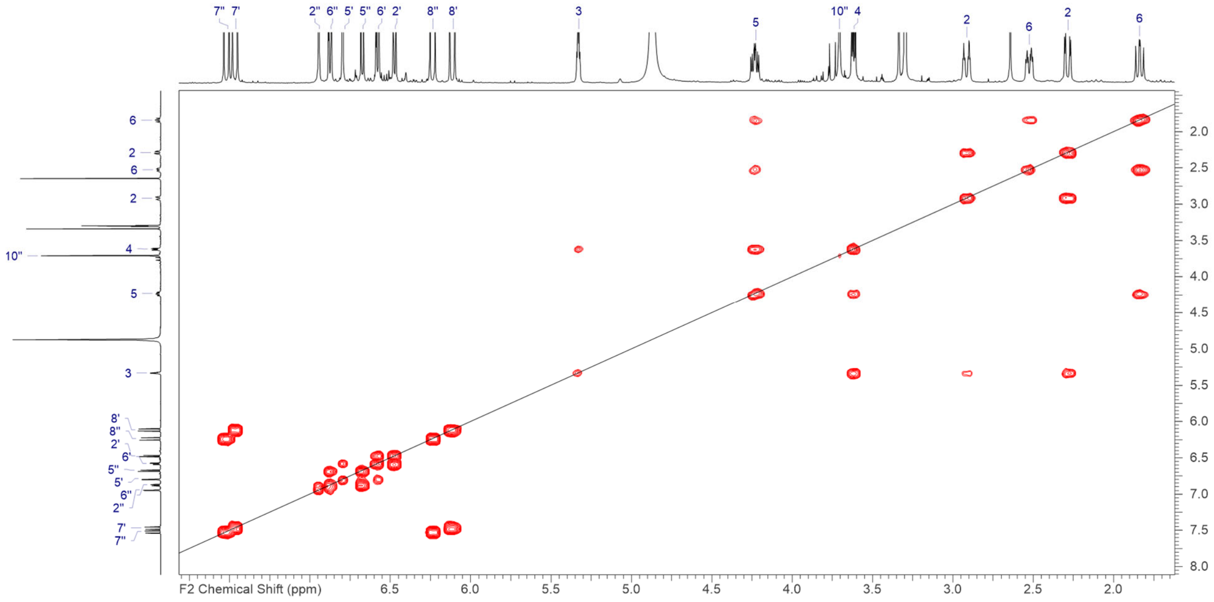

Figure S62. ^{1}H-^{1}H COSY spectrum of compound **11** (500 MHz, CD_3OD).

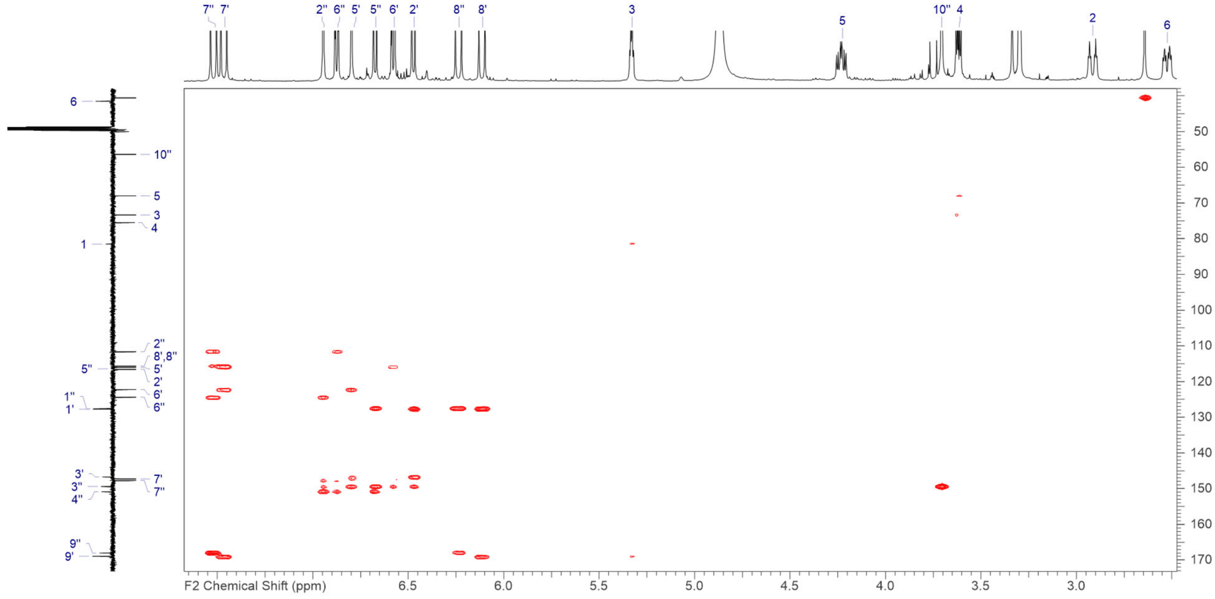

Figure S63. ^{1}H-^{13}C HMBC spectrum of compound **11** (500 MHz, CD_3OD).

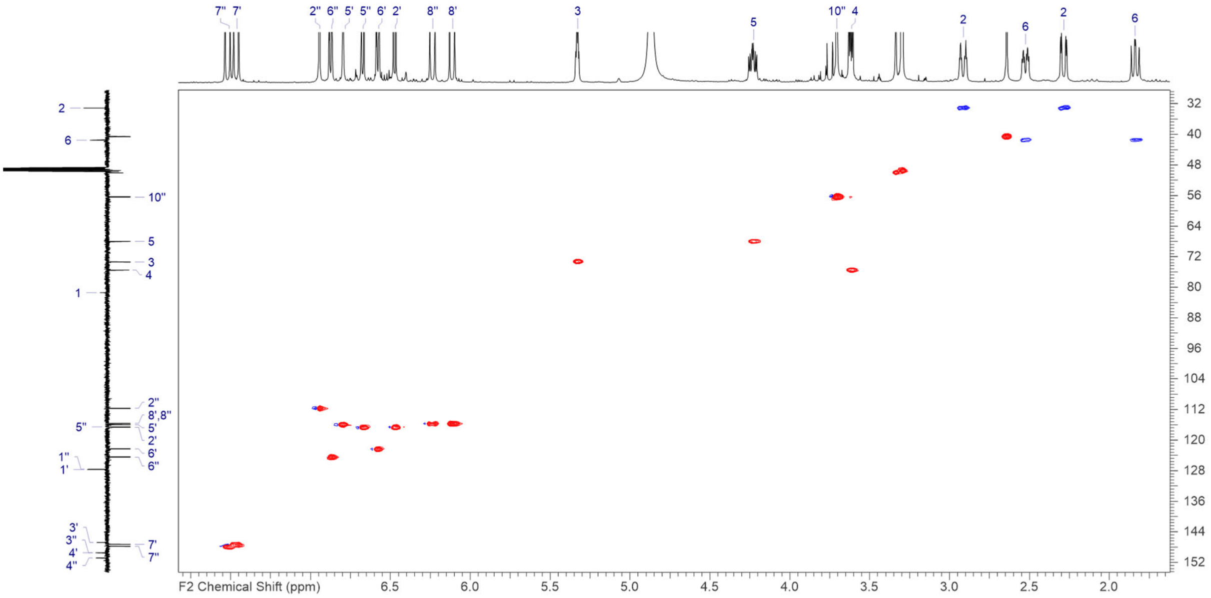

Figure S64. ^{1}H-^{13}C HSQC spectrum of compound **11** (500 MHz, CD_3OD).

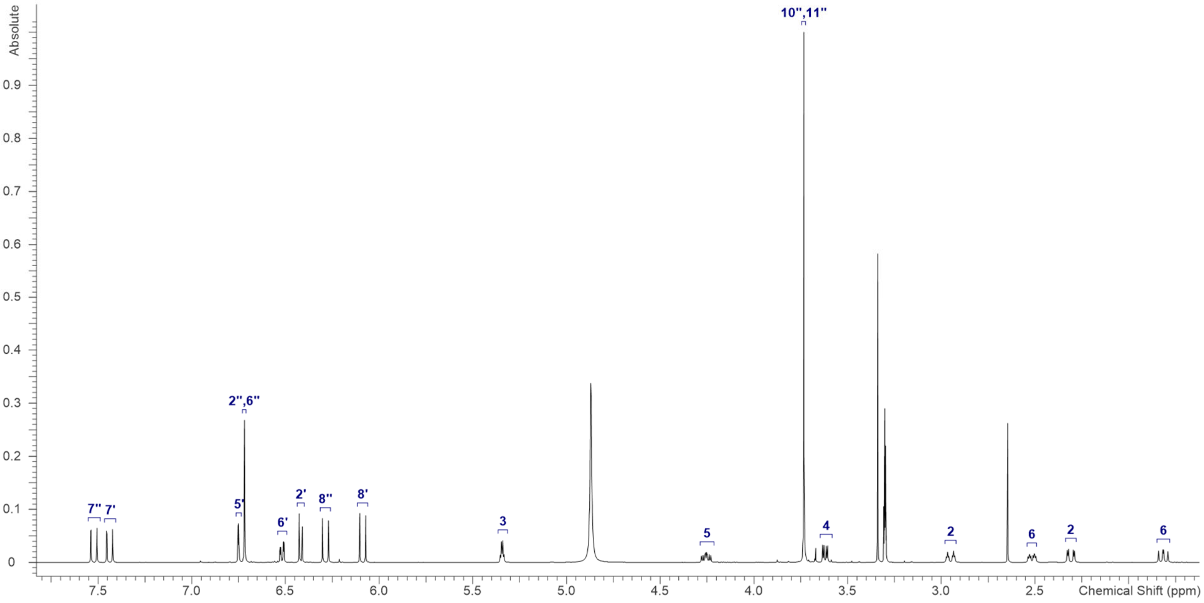

Figure S65. ^{1}H NMR spectrum of compound **12** (500 MHz, CD_3OD).

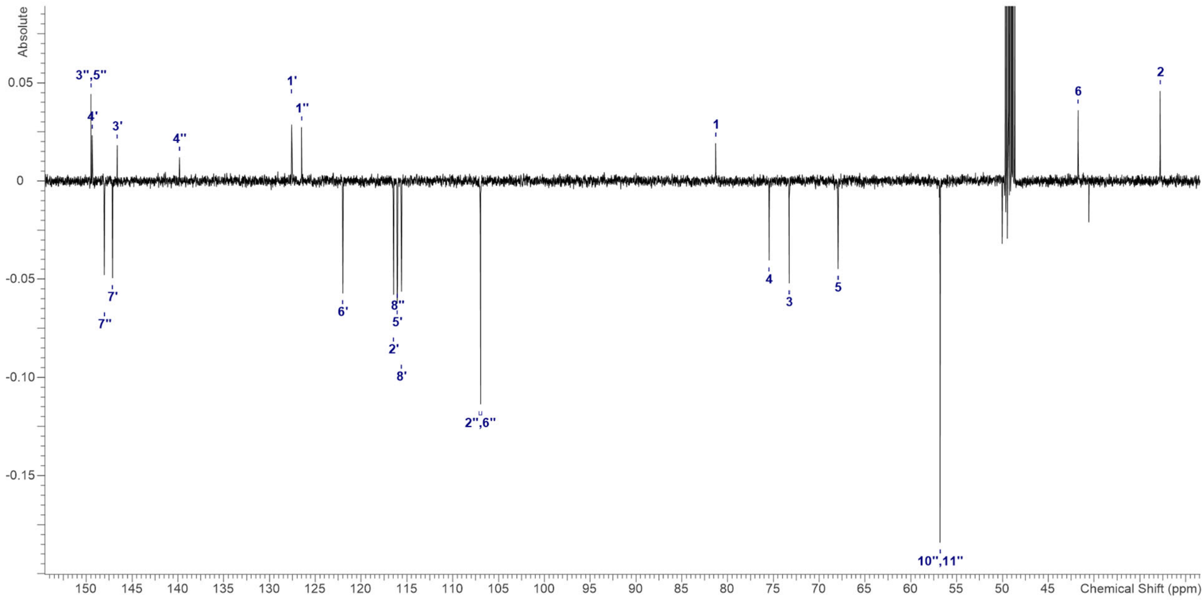

Figure S66. ^{13}C NMR spectrum of compound **12** (125 MHz, CD_3OD).

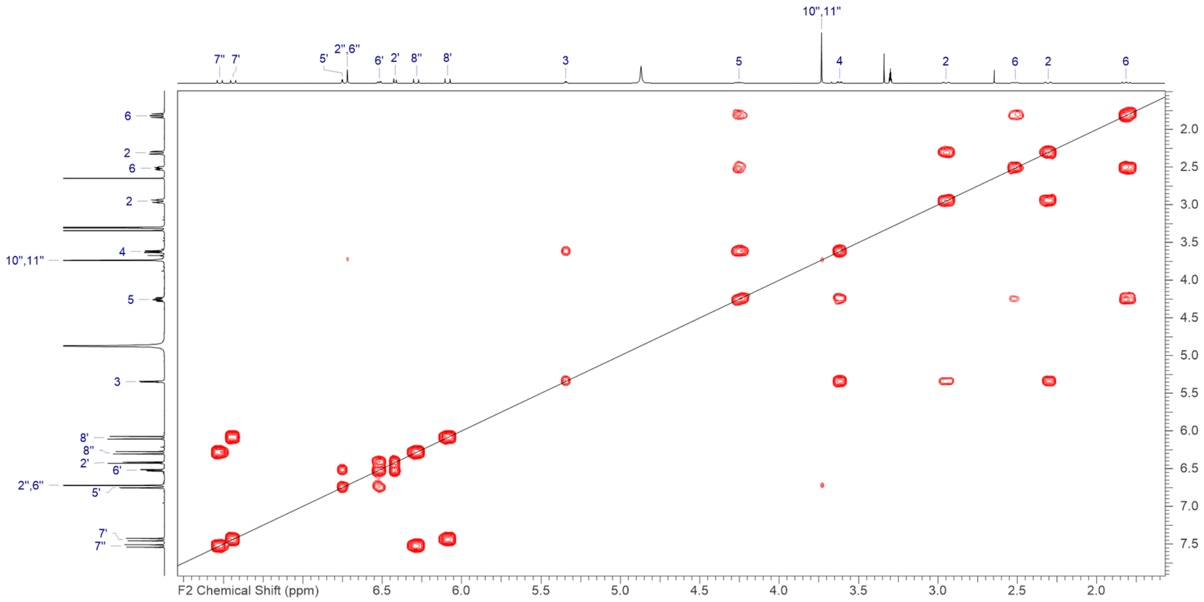

Figure S67. ^{1}H-^{1}H COSY spectrum of compound **12** (500 MHz, CD_3OD).

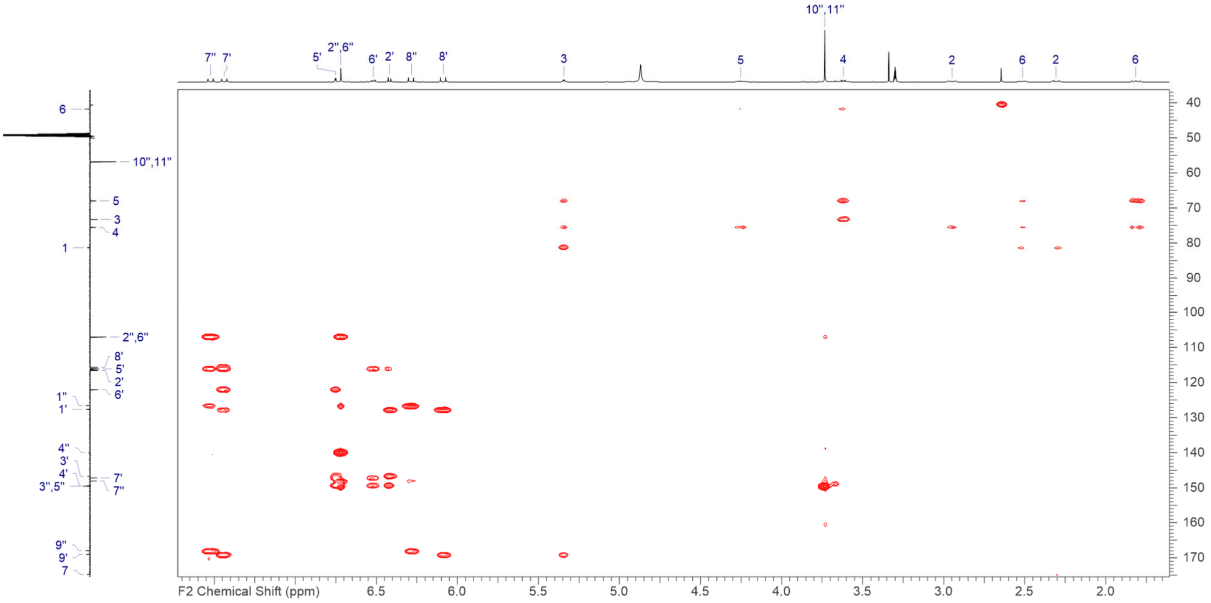

Figure S68. ^{1}H-^{13}C HMBC spectrum of compound **12** (500 MHz, CD_3OD).

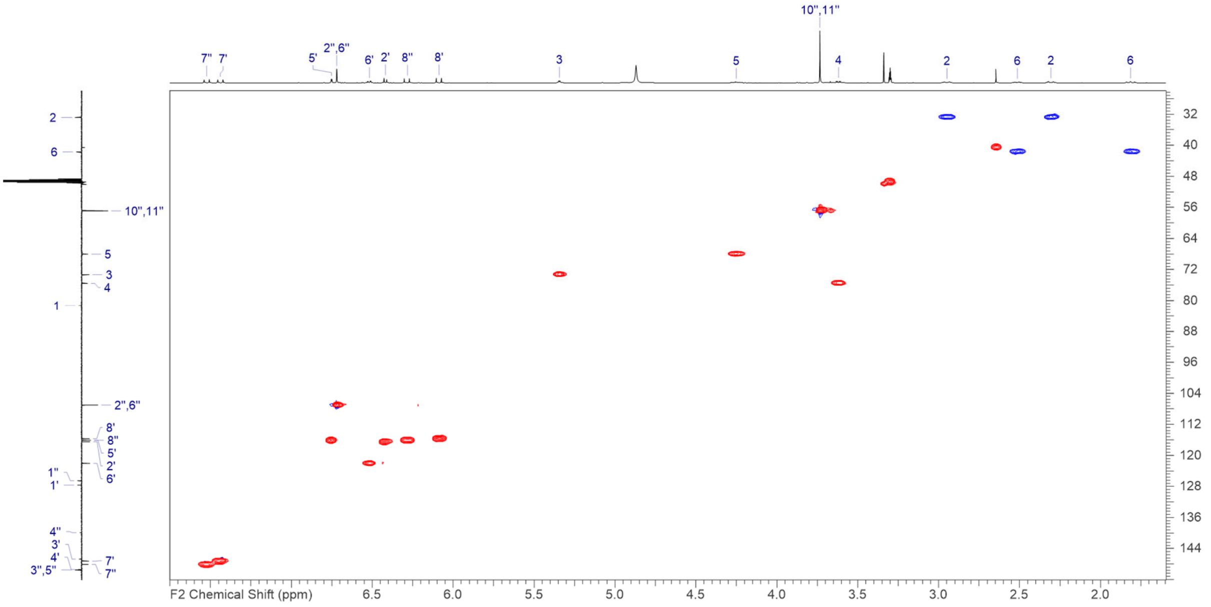

Figure S69. ^{1}H-^{13}C HSQC spectrum of compound **12** (500 MHz, CD_3OD).

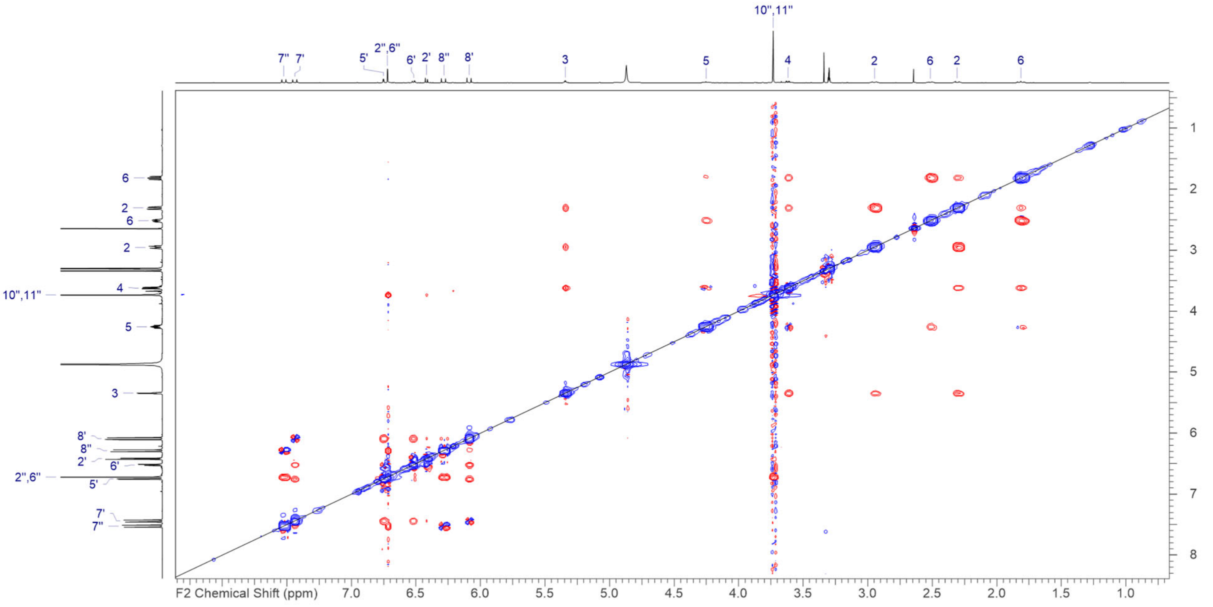

Figure S70. ¹H-¹H ROESY spectrum of compound **12** (500 MHz, CD_3OD).

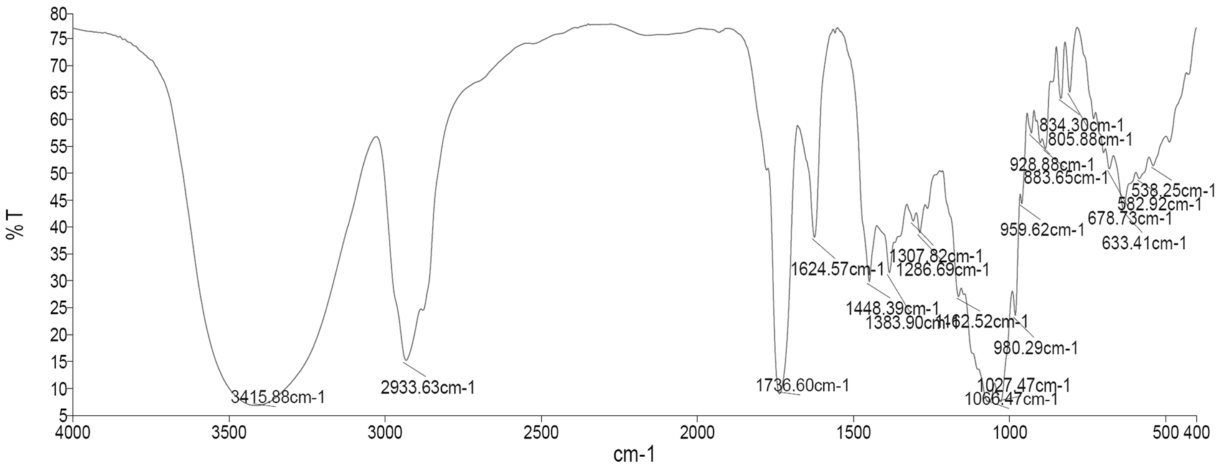

Figure S71. IR spectrum of compound **3**.

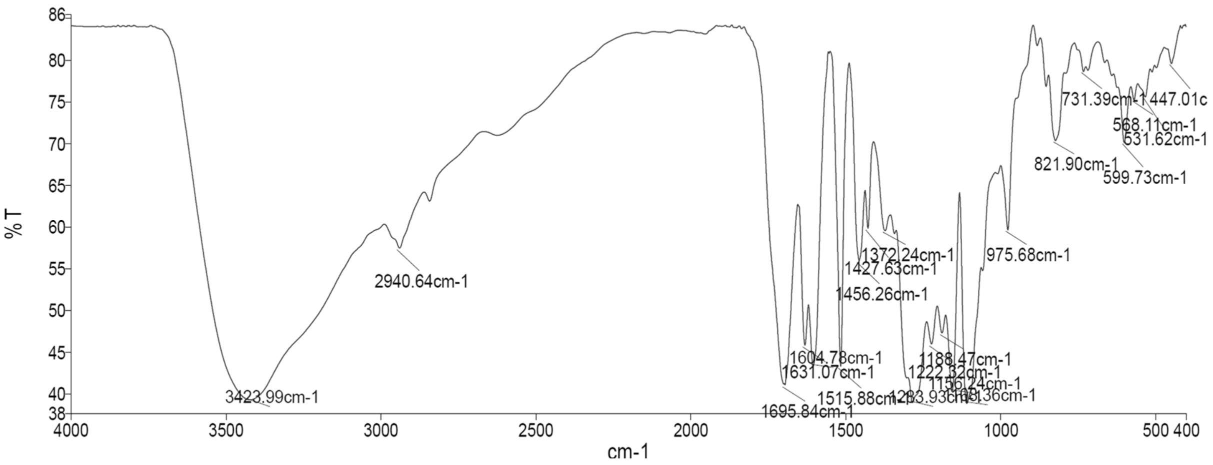

Figure S72. IR spectrum of compound **12**.

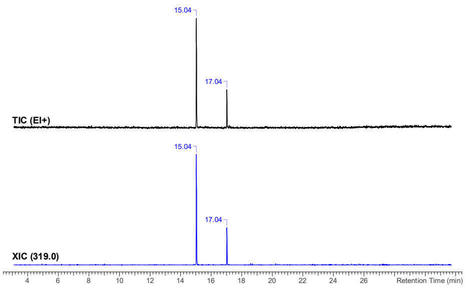

Figure S73. GC-MS Sugar analysis after hydrolysis of compound **1**.

2 peaks: 15.04 min, 17.04 min – *m/z* 319, 547 → D-Glucose

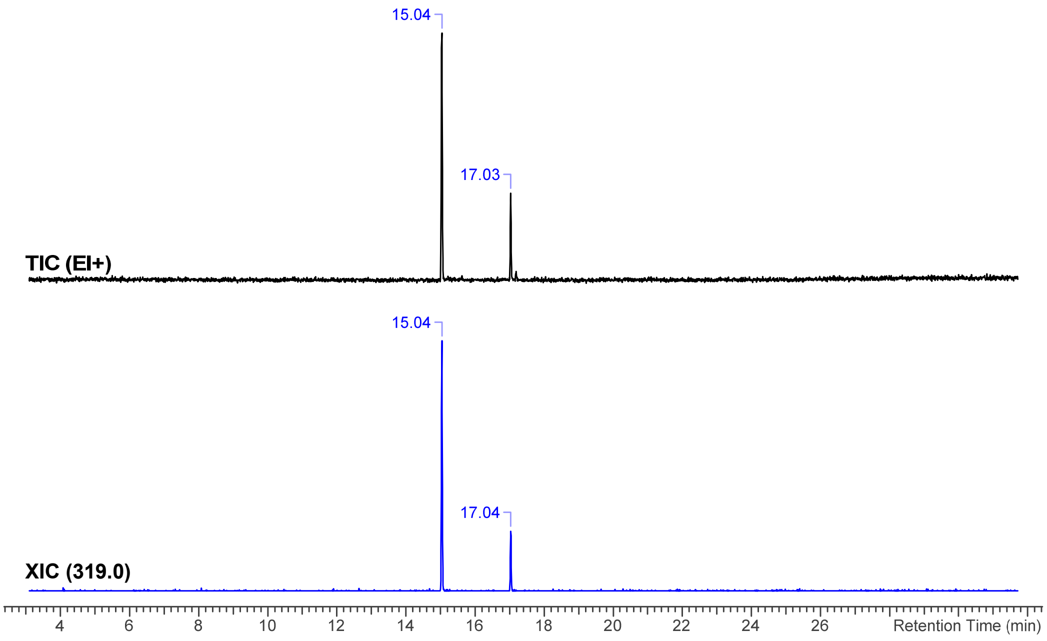

Figure S74. GC-MS Sugar analysis after hydrolysis of compound **2**.

2 peaks: 15.04 min, 17.04 min – *m/z* 319, 547 → D-Glucose

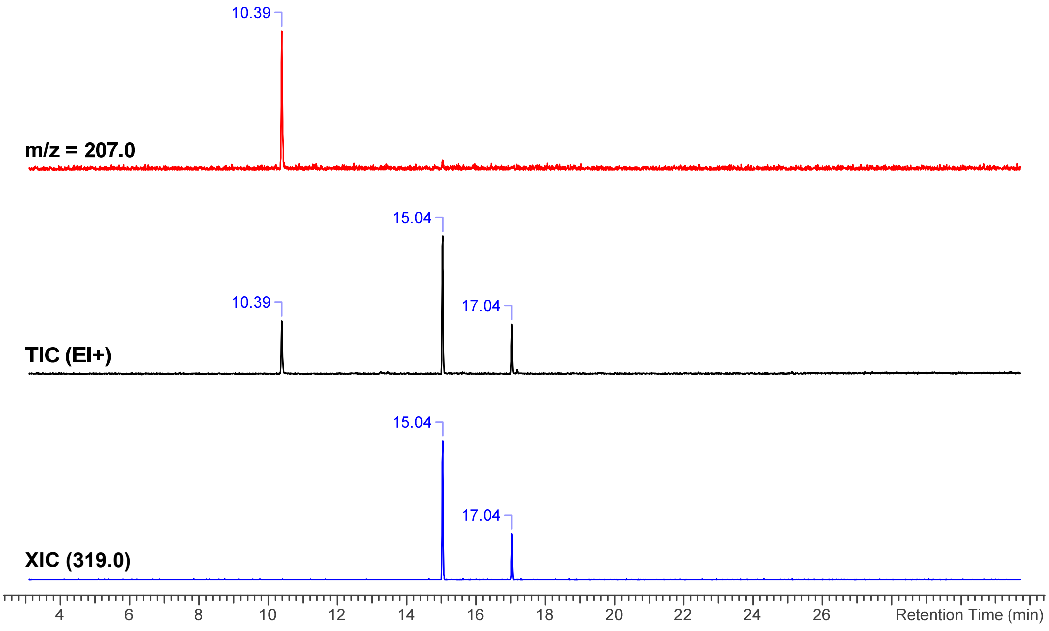

Figure S75. GC-MS Sugar analysis after hydrolysis of compound **3**.

2 peaks: 15.04 min, 17.04 min – *m/z* 319, 547 → D-Glucose
1 peak: 10.39 min – *m/z* 207, 292, 435 → L-Rhamnose

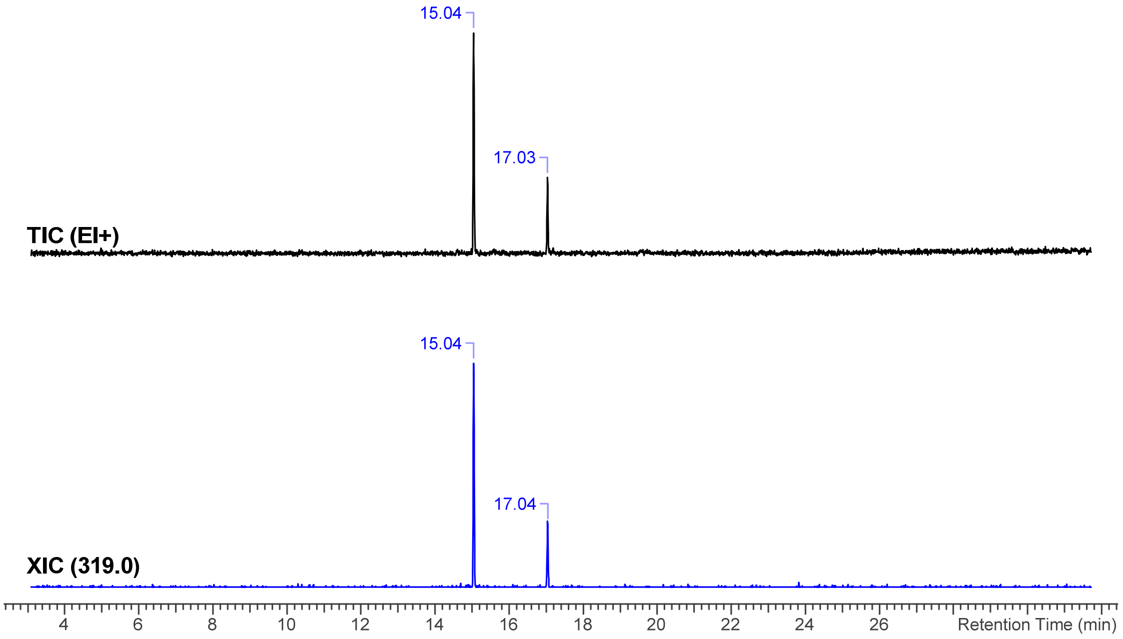

Figure S76. GC-MS Sugar analysis after hydrolysis of compound **9**.

2 peaks: 15.04 min, 17.04 min – *m/z* 319, 547 → D-Glucose

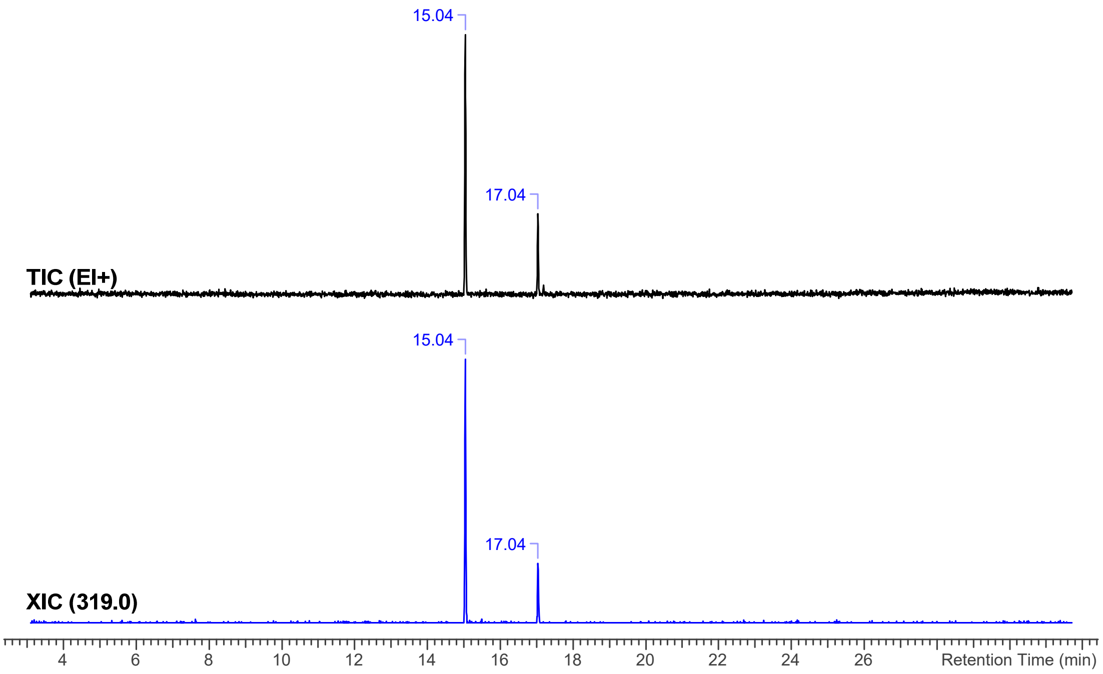

Figure S77. GC-MS Sugar analysis after hydrolysis of compound **10**.

2 peaks: 15.04 min, 17.04 min – *m/z* 319, 547 → D-Glucose
